# Data supporting phylogenetic reconstructions of the Neotropical clade Gymnotiformes

**DOI:** 10.1016/j.dib.2016.01.069

**Published:** 2016-02-06

**Authors:** Victor A. Tagliacollo, Maxwell J. Bernt, Jack M. Craig, Claudio Oliveira, James S. Albert

**Affiliations:** a Universidade Estadual Paulista – UNESP, Instituto de Biociências de Botucatu, Botucatu, SP 18618-970, Brazil; b University of Louisiana at Lafayette, Department of Biology, Lafayette, LA 70504-2451, USA

## Abstract

Data is presented in support of model-based total evidence (MBTE) phylogenetic reconstructions of the Neotropical clade of Gymnotiformes “Model-based total evidence phylogeny of Neotropical electric knifefishes (Teleostei, Gymnotiformes)” (Tagliacollo et al., 2016) [1]). The MBTE phylogenies were inferred using a comprehensive dataset comprised of six genes (5277 bp) and 223 morphological characters for an ingroup taxon sample of 120 of 218 valid species and 33 of the 34 extant genera. The data in this article include primer sequences for gene amplification and sequencing, voucher information and GenBank accession numbers, descriptions of morphological characters, morphological synapomorphies for the recognized clades of Gymnotiformes, a supermatrix comprised of concatenated molecular and morphological data, and computer scripts to replicate MBTE inferences. We also included here Maximum-likelihood and Bayesian topologies, which support two main gymnotiform clades: Gymnotidae and Sternopygoidei, the latter comprised of Rhamphichthyoidea (Rhamphichthyidae+Hypopomidae) and Sinusoidea (Sternopygidae+Apteronotidae).

**Specifications Table**

**Table:** 

Subject area	Biology, Genetics and Genomics
More specific subject area	Phylogenetics and Phylogenomics
Type of data	Primers, GenBank numbers, Morphological characters, Supermatrix, Scripts, Newick trees
How data was acquired	DNA extraction, Gene amplification, Sanger sequencing, morphological examinations.
Data format	Raw, filtered, and analyzed
Experimental factors	N/A
Experimental features	Sequencing of mitochondrial and nuclear genes and character coding of morphological traits
Data source location	South America
Data accessibility	Data is with this article and in the GenBank public repository at GenBank: 26616344

**Value of the data**

• Data summary for the most comprehensive phylogenetic study of Gymnotiformes to date, including an ingroup taxon sampling of 33 (94%) recognized genera and 120 (57%) of all valid species.
• New molecular sequences for 149 specimens and descriptions of morphological characters for 166 specimens.
• Supermatrix comprised of six genes (5277 bp) and 223 morphological characters used to reappraise relationships of Gymnotiformes

**Data**

The data provided below include supporting information to replicate phylogenetic analyses of Tagliacollo et al. [1]. The information is comprised of: (1) taxon sampling used in the phylogenetic analyses, (2) molecular and morphological datasets including GenBank accession numbers, description of morphological characters, and synapomorphies used to diagnose clades, and 3) detailed description of methodological procedures and parameters used in Tagliacollo et al. [1] to estimate phylogenetic relationships of the Neotropical clade Gymnotiformes. Furthermore, supplementary materials include the following: (1) computer scripts used in Tagliacollo et al. [1] to run MBTE analyses, (2) a matrix of morphological characters, (3) a supermatrix of combined molecular and morphological, (4) a list of analyzed material (specimens and lots), and (5) maximum-likelihood and bayesian phylogenetic trees.

**Experimental design, materials and methods**

## 1. Taxon sampling

Outgroups were chosen to cover a broad spectrum of ostariophysan diversity in terms of clade representation. Outgroups included nine major lineages of Otophysi represented by: *Carassius auratus*, *Erythrinus erythrinus*, *Serrasalmus rhombeus*, *Cyphocharax festivus*, *Charax tectifer*, *Pseudostegophilus nemurus*, *Brachyplatystoma juruense*, *Dianema longibarbis*, *Pterygoplichthys multiradiatus*. Ingroup taxon samples were chosen by using a clade-based approach to maximize the representation of phylogenetic diversity in Gymnotiformes.

Ingroup species are comprised of representatives of all major gymnotiform clades, including 33 of 35 (94%) recognized genera and 120 of 218 (55%) of all currently valid species. Voucher specimens for tissue samples were identified either directly by the authors, directly by curators and collection managers at contributing institutions, or by exchange of photographs. Species identifications of Genbank sequences were not reevaluated. Molecular vouchers and GenBank accession numbers are presented in Table 1.

## 2. Molecular dataset

### 2.1. DNA extraction, PCR amplification, and gene sequencing

Genomic DNA was extracted from tissues, fins or livers of specimens preserved in pure ethanol with the NucleoSpin^®^ 96 Tissue kit (Macherey-Nagel). Fragments of the mitochondrial genes 16S rRNA (16S-mit), Cytochrome Oxidase subunit I (COI-mit), Cytochrome B (CytB-mit), and the nuclear gene Zic family member 1 (ZIC-nuc) were amplified by one round of polymerase chain reaction (PCR), which was carried out in a volume of 25.0 μl consisting of: 2.5 μl of 10x Taq Buffer, 2.0 μl of dNTP mixture at 10 mM each, 1.5 μl of 50 mM MgCl_2_, 1.0 μl of each primer at 5 μM, 0.2 μl of Platinum^®^ Taq DNA Polymerase, 2.0 μl of template DNA (~50 ng), and 15.8 μl of double-distilled H_2_O. Fragments of the nuclear gene Recombination-Activating gene 2 (RAG2-nuc) and Recombination-Activating gene 1 (RAG1-nuc) were amplified by nested-PCRs. Each round of the two PCR was carried out in a volume of 25.0 μl consisting of: 2.5 μl of 10x Taq Buffer, 2.0 μl of dNTP mixture at 10 mM each, 2.0 μl of 50 mM MgCl_2_, 1.5 μl of each primer at 5 μM, 0.2 μl of Platinum^®^ Taq DNA Polymerase, 2.0 μl of template DNA (~50 ng), and 14.8 μl of double-distilled H_2_O. Cycles of PCR for the mitochondrial genes consisted of five steps: (1) 60s for enzyme activation at 94 °C, (2) 30s of denaturation at 94 °C, (3) 60s of annealing at 56 °C (16S-mit), 54–58 °C (COI-mit), or 50–52 °C (CytB-mit), (4) 80s of extension at 72 °C, and (5) 300 s of extension at 72 °C. The steps 2–4 were repeated 35 times. Cycles of PCR for the nuclear genes consisted of six steps: (1) 60s for enzyme activation at 94 °C, (2) 30s of denaturation at 94 °C, (3) two start cycles of 60s each at 56 °C, 50 °C, 52 °C, 54 °C (RAG2-nuc, RAG1-nuc) and 54 °C, 50 °C 52 °C, 56 °C (ZIC-nuc), (4) 60s of annealing at 50 °C (RAG2-nuc, RAG1-nuc) and 52 °C (ZIC-nuc) and (5) 80s of extension at 72 °C, and (6) 300s of extension at 72 °C. The steps 2, 4 and 5 were repeated 35 times. PCR products were visually identified on a 1% agarose gel. Sequencing was held at Beckman Coulter Genomics Facility. The list of primers is shown in Table 2.

### 2.2 Sequence alignments

Forward and reverse sequences were assembled in Geneious 5.5.6. The IUPAC ambiguity code of nucleotides was applied in cases where nucleotide identity was dubious. We combined newly generated data with available sequences from previous studies [2], [3], [4], [5], [6], [7]. Each gene was independently aligned using MAFFT 5.3 [8] under default parameters. To detect potential errors such as amplification of pseudogenes, paralogous copies or potential laboratory cross-contamination, each gene alignment was analyzed in PhyML 3.0 [9]. Sequences suspiciously misplaced in the resulting gene trees were then re-amplified.

## 3. Morphological dataset

### 3.1. Description of characters used in the morphological dataset

1. Body shape 1.0: body laterally compressed, body width at pectoral fin base less than 70% its depth. 1: Body cylindrical or subcylindrical, roughly circular in cross section, body depth at pectoral girdle approximately equal to its width.
2. Body shape 2.0: body laterally compressed. 1: Body dorsoventrally flattened. Newly coded herein.
3. Body shape profile. “Body Depth,” character 2 in Albert, 2001. 0: Body relatively deep in profile, depth at pectoral girdle more than 11% total length. 1: Body elongate, slender, depth less than 11% total length.
4. Snout length short. 0: preorbital length about one-third total head length in mature specimens. 1: Snout short, preorbital length less than one-third total head length ([10]-Fig. 13).
5. Snout long. 0: Length of the snout (preorbital length) about one-third total head length in mature specimens. 1: Snout elongate, frontal, vomer and anterior portion of parasphenoid elongate; preorbital length longer than one-third total head length or greater in mature specimens ([10]-Figs. 11–17).
6. Gape large. 0: Rictus of mouth extends ventral to nasal capsule, gape forming less than one-third total head length. 1: Rictus extends posterior to a vertical through eye, gape forming more than one-third total head length.
7. Gape short. 0: Rictus extends ventral to nasal capsule, gape more than three times eye diameter, oriented parallel with long axis of head. 1: Rictus extends to a vertical with mental symphysis, gape very small, less than twice diameter of eye, oriented oblique to long axis of head.
8. Oral opening in adults. 0: Upper and lower jaws of equal length, oral aperture terminal. 1: Lower jaw extends anterior to upper, oral aperture superior. 2: Upper jaw extends anterior to lower, oral aperture inferior.
9. Position of nasal capsule. 0: Nasal capsule, including olfactory epithelium and olfactory sensory neurons, positioned relatively posteriorly on snout; located closer to eye than to anterior tip of snout; posterior nares closer to anterior margin of eye than to anterior nares. 1: Anterior position of nasal capsule; located closer to tip of snout than to eye; posterior nares closer to anterior nares than to anterior margin of eye ([10]-Fig. 19).
10. Position of anterior nares. 0: Anterior nares situated on dorsal surface of snout, narial opening oriented dorsally. 1: Anterior nares located very close to or within gape, narial opening oriented anteroventrally ([11]-Figs. 69-B and 78-B).
11. Anterior narial pore. 0: Anterior narial opening situated at end of a short tube. 1: Anterior narial opening sessile, its rim flush with surrounding integument.
12. Posterior narial pore. 0: Posterior narial pore present. 1: Posterior narial pore absent [12].
13. Eye size. 0: Eye and optic tract large; about two eye diameters into postorbital head length. 1: Eye and optic tract small; more than five eye diameters into postorbital head length ([13]-character 17).
14. Position of eye. 0: Surface of eye not covered by epidermis in adults; free orbital margin. 1: Eye completely covered by epidermis in adults; orbital margin not free ([14]-character 17).
15. Anal fin membrane. 0: Evenly pigmented. 1: With pale posterior patch. 2: Anal fin membrane striped. ([15]).
16. Oblique pigment bands. 0: Body pigmentation evenly distributed along longitudinal axis. 1: Multiple (13–50) pale bands with straight margins of alternating high and low melanophore density along lateral surface of body, oriented at an oblique angle to longitudinal body axis ([11]-Figs. 70–76, 81–84; [16]-Fig. 1). Bands sometimes interrupted by patches of depigmented integument, resulting in a distribution of blotches arranged in oblique bands along the lateral surface of body ([16]). 2: Oblique pigment bands along longitudinal axis with wavy margins ([15]).
17. Vertical pigment lines. 0: Vertical pigment lines absent along longitudinal body axis. 1: Thin vertical pigment lines present along longitudinal body axis. Newly coded herein.
18. Vertical pigment bars. “Saddle-shaped bars”, character 5 in [10]). 0: Absent from dorsum. 1: 1–10 dark bars across mid-dorsal surface extending as vertical bands onto lateral surfaces.
19. Caudal Peduncle Spot. 0: Pale spot absent from base of caudal region. 1: Pale spot present at base of caudal region. Newly coded herein.
20. Longitudinal lines. 0: Absent. 1: 2–3 thin dark lines extending posteriorly along the lateral body surface ([17]). 2: A white narrow stripe extending parallel to the base of the anal-fin pterygiophores, and then posteriorly along the lateral midline ([18]).
21. Pigment contrast. 0: Body surface yellow or pale brown, lacking high contrast dark brown or black and white pigments. 1: High contrast dark brown or black and white pigments on body surface.
22. White posterior bars. 0: White or pale bars absent from caudal region. 1: White or pale bars present on caudal region as observed in members of the *Apteronotus albifrons* species group. Newly coded herein.
23. White mid-sagittal pigments. 0: All mid-sagittal surfaces brown. 1: Mid-sagittal region of dorsal and mental surfaces bright white.
24. Antorbital stripe. 0: Melanophores on snout distributed evenly. 1: Melanophores absent from narrow band passing lateral to nares ([11]-Fig. 90).
25. Pigment distribution. 0: Pigments distributed homogeneously over body surface. 1: Black and white pigments distributed unevenly over body surface, darker and paler areas grading into one another; integument with a marbled or mottled appearance.
26. Body translucence. 0: Body opaque in living and formalin-fixed specimens, lateral body surface covered with brown melanophores. 1: Body translucent in living specimens, yellow or pink hue in living specimens, yellow or hyaline in formalin-fixed specimens, melanophores sparse or absent on lateral body surface.
27. Branchial opening. 0: Branchial opening extends along entire posterior margin of opercle, from isthmus to pectoral fin insertion. 1: Vertical extent of branchial opening restricted to region around pectoral fin base; ventral portion reduced by a dorsolateral continuation of epidermis from isthmus ([14], [11]).
28. Pseudotympanum. 0: Sixth vertebra partially covered by superior oblique. 1: Sixth vertebra not covered by superior oblique.
29. Body squamation. 0: Scales present on body and head. 1: Body devoid of scales ([13]-character 124).
30. Scales on middorsum. 0: Scales present on middorsal surface of body. 1: Scales absent from head, anterior portion of dorsal midline, and area dorsal to pectoral fins. 2: Scales absent along entire middorsum (modified from [10]-character 15).
31. Scale shape. 0: Scales dorsal to lateral line ovoid at mid-body, their long axes oriented parallel with long axis of body. 1: Scales dorsal to lateral line rhomboid, their long axis oriented oblique to long axis of body, their dorsoventral axes longer than their longitudinal axes.
32. Lateral line. 0: Lateral line complete. 1: Lateral line incomplete ([19]).
33. Lateral line pores. 0: Posterior lateral line canal pores short; tube length less than three pore diameters. 1: Posterior lateral line pores tubular; tube length more than three times pore diameter.
34. Lateral line ventral rami. 0: No ventral rami of lateral line. 1: Numerous ventral rami extending parallel with lateral line.
35. N°. ventral rami. 0: Median 14 or less. 1: Median 15 or more.
36. Adult dentition. 0: Oral teeth present in juveniles and adults. 1: Oral teeth present in juveniles, lost and not replaced during development.
37. Tooth shape. 0: Teeth in both jaws conical, with a broad base tapering toward the cusp. 1: Teeth in both jaws villiform, each tooth a long cylindrical shaft with a narrow base ([14]- character 2). 2: Teeth with triangular, arrow-head shape [20].
38. Tooth tip shape. 0: Tips of teeth straight or directed posteriorly (decurved). 1: Tips of teeth directed anteriorly (recurved). [21].
39. Premaxilla size. 0: Large. Lateral margin of premaxilla longer than lateral margin of maxilla, premaxilla extends posterodorsal to articulation of maxilla with autopalatine; articular surface of maxilla with autopalatine oriented anterodorsally. 1: Small. The anterodorsal orientation of the articular surface of the maxilla with the autopalatine is a consequence of the large size of the premaxilla and the associated posterior position of the maxilla.
40. Premaxillary teeth. 0: Teeth present on premaxilla of adults. 1: Premaxillary dentition reduced or lost.
41. Maxillary dentition. 0: A single row of 6–10 conical teeth in along outer margin of maxilla. 1: No teeth on maxilla.
42. Maxilla size. “Orientation and Shape of Maxilla”-character 30 in [10]. 0: Maxilla robust, approximately as wide as deep at its midlength; descending blade at least twice as broad near posterior end as near articular surface with autopalatine; main axis straight in vertical plane, ventral margin straight in lateral view; articular surface with autopalatine facing dorsomedially. 1: Maxilla thin, more than twice as deep as wide at its midlength; descending blade relatively narrow, tapering evenly towards its distal tip; main axis curved in vertical plane, sickle-shaped in lateral view; articular surface with autopalatine facing dorsoposteriorly.
43. Anterior maxillary process. 0: Anterior process of maxilla absent. 1: Anterior process of maxilla extends anterior to articulation of maxilla and autopalatine, forming a tapered process, its ventral margin continuous with descending blade of maxilla; maxilla forked in lateral view. 2: Anterior process of maxilla cartilaginous; ventral margin of descending blade extends to articulation of maxilla with autopalatine, forming anterior border of maxilla; maxilla crescent shaped in lateral view ([10]- Figs. 6–7).
44. Maxillary articulation with palatine. 0: Articular surface of maxilla with autopalatine sessile, continuous with dorsal margin of maxilla; ethmopalatine cartilage forming a discrete quadrangular block bridging articulation of autopalatine and maxilla. 1: Articular surface of maxilla on a stalk, articulation with autopalatine at end of a bony process; ethmopalatine cartilage a small block attached firmly to articular head of maxilla ([10]-Figs. 6–7; [13]-Fig. 3).
45. Anterior maxillary shelf. 0: Anterior process of maxilla extending as a shelf of bone less than one-third the length of the descending blade. 1: Anterior process of maxilla large and broad, extending more than one half the length of the descending blade in mature specimens.
46. Maxilla descending blade. 0: Descending blade of maxilla broad and curved; maxilla sickle-shaped in lateral view. 1: Descending blade of maxilla broad, connective tissue membrane along its anteroventral margin ossified to form a thin shelf; anterior portion of maxilla rhomboid in lateral view.
47. Maxilla descending blade. 0: Ventral margin of descending blade curves evenly towards its distal tip. 1: Ventral margin of descending blade with a sharp angle about two-thirds distance to its tip; ventral margin posterior to this angle relatively straight. 2: Anteroventral margin of descending blade not ossified; distal half of blade extending as a narrow process with a sharp point at its distal tip
48. Maxillary-anguloarticular ligament. 0: Ligament extends between maxilla, adjacent to its articulation with autopalatine, and a part of *Adductor mandibulae* muscle. 1: Ligament extends between maxilla and dorsal tip of anguloarticular at coronoid process of mandible ([13]-character 45).
49. Rows of dentary teeth. 0: A single row of teeth on dentary. 1: Teeth on dentary arranged in two to three rows at its midlength.
50. Dentary gracile. 0: Dentary robust, posterodorsal process rounded, ventral margin straight or slightly convex in lateral view. 1: Dentary gracile, posterodorsal process tapering to a point (except in *Adontosternarchus sachsi*), ventral margin concave.
51. Dentary dorsal margin. 0: Dorsal margin of dentary straight or convex. 1: Dorsal margin of dentary concave. New character. See Figs. in [11], [22].
52. Dentary dorsal process. 0: No dorsal process on dentary. 1: Dorsal process on posterior region of dentary. New character. See Figs. in [11], [22].
53. Dentary filamentous. 0: Dentary quadrangular, less than twice as long as deep. 1: Dentary elongate and filamentous, more than four times as long as deep.
54. Dentary hook. 0: Ventral margin of dentary straight to its contact with anguloarticular. 1: Ventral margin of anterior portion of dentary bearing a posteriorly oriented process (“dentary hook”), a ventral extension of the medial surface of dentary where it covers the anterior portion of Meckel׳s cartilage ([11]-Fig. 66).
55. Dentary teeth size. 0: Teeth on posterior half of dentary roughly equal in size to anterior teeth. 1: Teeth on posterior half of dentary twice the size of anterior teeth. Newly coded herein, see de [23]-Fig. 2.
56. *M. Adductor mandibulae*. 0: Belly of *Adductor mandibulae* muscle composed of muscle fibers and tendons. 1: Belly of *Adductor mandibulae* muscle with ossified intermuscular bones, oriented parallel to main axis of muscle fibers ([24]).
57. *M. Adductor mandibulae*. 0: Ventrolateral branch of *m. Adductor mandibulae* (ostariophysan A_1_) inserts exclusively on maxilla; two discrete muscle bundles insert on oral jaws; A_1_ inserts exclusively on maxilla, and A_2_ on dentary. 1: Additional insertion of A_1_ on first infraorbital ([25]-Fig. 1; see [13]-character 127).
58. Anterior limb anguloarticular. 0: Anterior limb of anguloarticular longer than posterior limb, measured as distance from dorsal-most point of contact with dentary to anterior and posterior margins. 1: Anterior limb of anguloarticular shorter than posterior limb.
59. Posterior limb anguloarticular. 0: Posterior limb of anguloarticular small; its contribution to ventral margin of mandible shorter than that of retroarticular. 1: Posterior limb of anguloarticular large; its ventral margin longer than that of retroarticular (modified from [11], 1994).
60. Mesethmoid neck. Character 52 in [10]. 0: Entire length of mesethmoid broad in dorsal view, width of anterior tip approximately equal to width of region between nasal capsules. 1: Mesethmoid narrow near anterior end, forked in dorsal view, narrower between nasal capsules than in more posterior portions ([13], character 5-Fig. 3).
61. Mesethmoid length. 0: Length of mesethmoid less than length of antorbital region of frontal. 1: Mesethmoid elongate, its length greater than antorbital region of frontal.
62. Mesethmoid, tip size. 0: Anterior tip of mesethmoid robust, anterodorsal surface (anterior to ventral ethmoid) broad and concave, with a medial groove located between two large anterolateral processes (forming articulation with premaxillae). 1: Anterior tip of mesethmoid small, anterodorsal surface narrow, with a median knob-shaped process directed anteriorly between two small lateral processes ([13]-Fig. 3E and F).
63. Mesethmoid, tip shape. 0: Portion of mesethmoid anterior to ventral ethmoid horizontal; its dorsal surface anterior and posterior to ventral ethmoid approximately parallel; its ventral surface parallel with dorsal surface. 1: Portion of mesethmoid anterior to ventral ethmoid flexed ventrally in mature specimens; its dorsal surface anterior and posterior to ventral ethmoid at an oblique angle; its ventral surface oblique to dorsal surface ([26]-Fig. 4).
64. Mesethmoid tip groove. 0: Anterior surface of mesethmoid flat or convex. 1: Anterior surface of mesethmoid concave.
65. Ventral ethmoid size. 0: Ventral ethmoid gracile. 1: Ventral ethmoid robust. ([10]- Figs. 13–14)
66. Ventral ethmoid lateral process. 0: Lateral process of ventral ethmoid extends as a blunt posterolateral process articulating with lateral ethmoid cartilage. 1: No lateral process of ventral ethmoid; ventral ethmoid not contacting lateral ethmoid cartilage.
67. Ventral ethmoid lateral process shape. 0: Lateral process of ventral ethmoid narrow, flattened horizontally, posterior surface articulating with lateral ethmoid cartilage. 1: Lateral process of ventral ethmoid robust, posterior surface forming articulation with lateral ethmoid cartilage broad and rounded, covered by a cartilage cap. 2: Lateral process of ventral ethmoid large and fan-shaped (new character state).
68. Median septum of ventral ethmoid. 0: Portion of ventral ethmoid ossified within medial nasal septum approximately as long as deep; posterior margins of median septum and lateral process of ventral ethmoid approximately equal. 1: Ossified median septum of ventral ethmoid elongate in mature specimens, longer than deep, extending posterior to posterior margin of lateral process.
69. Ventral ethmoid-Vomer. 0: Ventral ethmoid fused with vomer during growth. 1: Ventral ethmoid and vomer not fused in adults ([24]).
70. Dermal vomer. 0: Dermal vomer extends from posterior margin of ventral ethmoid to parasphenoid. 1: Dermal vomer not ossified.
71. Ethmoid cartilage. 0: Ethmoid cartilage anterior to lateral ethmoid longer than deep; antorbital region of snout longer than deep. 1: Ethmoid cartilage deeper than long; antorbital region of snout about as deep as long ([26]).
72. Lateral ethmoid size. 0: Lateral ethmoid a large endochondral ossification in the antorbital region, arching laterally over Profundus (V1) nerve, with four margins; anterolateral process contacting ventral ethmoid, posteromedial process contacting parasphenoid, dorsomedial margin contacting frontal, and anteromedial margin contacting mesethmoid ([13]-Fig. 2; [18]-character 15, Fig. 4). 1: Lateral ethmoid reduced in size; four peripheral margins not contacting other bony surfaces ([27]-character 6a, Fig. 3).
73. Lateral ethmoid. 0: Lateral ethmoid ossified. 1: Lateral ethmoid not ossified ([26], [27]-character 7a).
74. Base lateral ethmoid. 0: Lateral ethmoid broad; length of its ventral margin more than one half length of its anterior margin. 1: Lateral ethmoid narrow or tubular; length of its base less than one-third length of its anterior margin ([27]-character 7b, Fig. 9).
75. Nasal. 0: Nasal narrow. 1: Nasal broad.
76. Sclerotic bones. 0: Eyes circumscribed by a series of sclerotic bones; 1: Sclerotic bones absent ([13]-character 17).
77. Antorbital process frontals. 0: Lateral margin of frontal smooth in region anterior and dorsal to orbit. 1: Lateroventral process of frontals anterior to orbit ([18]-character 20, Fig. 4).
78. Dorsal margin of frontals. 0: Dorsal margin of frontal straight or slightly convex in lateral profile. 1: Portion of frontal anterior to orbit concave in lateral profile.
79. Cranial fontanels. 0: Paired frontals separated by two gaps along their medial borders; two large midsaggital openings present on dorsal surface of neurocranium. 1: Frontals in contact with each other along the entire extent of their medial margins in mature specimens ([28]-plate 17, Fig. 1).
80. Sphenoid region. 0: Sphenoid region of neurocranium less than one-third total head length in mature specimens, combined axial length of orbitosphenoid and pterosphenoid about equal to length of preorbital region. 1: Sphenoid region of neurocranium more than one-third total head length, combined axial length of the orbitosphenoid and pterosphenoid bones greater than preorbital region. 2: Sphenoid region of neurocranium more than one-third total head length, combined axial length of the orbitosphenoid and pterosphenoid bones greater than preorbital region, orbit positioned at anterior third of head.
81. Orbitosphenoid shape. 0: Orbitosphenoid well ossified in median nasal septum, orbitosphenoid broad, its ventral margin longer than its dorsal margin. 1: Anterior margin of orbitosphenoid not ossified, orbitosphenoid narrow, its ventral margin about as long or shorter than its dorsal margin.
82. Orbitosphenoid margin. “Sphenoid fenestra” (Character 74) in [10]. 0: Posterior margin of orbitosphenoid broadly contacting pterosphenoid, separated by means of a narrow block of cartilage in mature specimens. 1: Posterior margin of orbitosphenoid not contacting pterosphenoid, except between dorsal portion of their common margin; presence of an unmineralized fenestra between orbitosphenoid and pterosphenoid ([28]-Plate 17, Fig. 2).
83. Sphenotic process. 0: Dorsolateral margin of sphenotic straight, anterior margin underlies frontal. 1: Dorsolateral margin of sphenotic bearing a transversely oriented crest or process exposed on dorsolateral edge, anterior margin not underlying frontal ([14]-character 3, Figs. 3–4).
84. Parasphenoid lateral process. 0: Lateral margins of parasphenoid extending as broad dorsolateral processes anterior to prootic, extending to a horizontal with trigeminal foramen. 1: Lateral margins of parasphenoid not extending to a horizontal with trigeminal foramen.
85. Parasphenoid ventral margin. 0: Ventral margin of parasphenoid straight in lateral profile in mature specimens; without a pronounced flexure at conjunction between otic and sphenoid regions of neurocranium. 1: Ventral margin of parasphenoid flexed sharply on either side of the basicranial region; ventral margin of sphenoid region oblique relative to long axis of neurocranium.
86. Parasphenoid dorsal margin. 0: Dorsal margin broad. 1: Dorsal margin narrow ([29], [19]).
87. Parasphenoid process. 0: Anteroventral margin of parasphenoid smooth. 1: Parasphenoid with anteroventral process.
88. Posttemporal fossa. 0: Epioccipital, pterotic and parietal bones contact one another along their mutual margins, forming a bony surface in posttemporal region of neurocranium; lateral surface of otic capsule enclosed. 1: Epioccipital, pterotic, and parietal bones not contacting one another along their mutual margins, forming a fossa in posttemporal region; lateral surface of otic capsule exposed [14], [30].
89. Supraoccipital crest. 0: Dorsal margin of supraoccipital crest even with dorsal margin of parietals. 1: dorsal margin of supraoccipital crest exceed dorsal margin of parietals [31].
90. Cranial skeleton texture. 0: Surface of endochondral and dermal ossifications of cranial skeleton composed of lamellar or cancellous bone. 1: Surface of many cranial bones pitted and/or reticular in appearance, excavated during ontogeny to form small pits and vesicles.
91. Nasal loop. 0: Commissure connecting infraorbital and supraorbital laterosensory canals oriented vertically, embedded in integument immediately anterior to eye; antorbital and first infraorbital bones situated near posterior nares. 1: Commissure between infraorbital and supraorbital canals extended anteriorly, forming a loop ventrolateral to nasal capsule; antorbital and first infraorbital bones situated near anterior nares ([11]).
92. Infraorbital subnasal extension. 0: Anterior portion of infraorbital canal extending anterior from first infraorbital ventral to nasal capsule; anterior canal pore of infraorbital canal situated anterior to first infraorbital. 1: Anterior extension of infraorbital canal shorter than width of canal pore; anterior canal pore of infraorbital canal situated near first infraorbital.
93. Infraorbital–supraorbital prenasal commissure. 0: Infraorbital–supraorbital prenasal commissure absent. 1: Infraorbital–supraorbital prenasal commissure present ([10]-Fig. 19).
94. Antorbital. 0: Infraorbital canal not extending onto antorbital. 1: Infraorbital canal extending onto antorbital ([30]).
95. Antorbital size. 0: Antorbital small, positioned directly anterior to orbit; its posteroventral process smaller than maxilla; does not contact autopalatine. 1: Antorbital large; its ventral portion larger than maxilla; expanded dorsal portion contacts autopalatine ([27]-character 18).
96. Antorbital shape. 0: Antorbital not crescent-shaped. 1: Antorbital crescent-shaped ([24]).
97. Infraorbital canal plates. 0: Canal bearing portion of infraorbital bones slender and tubular. 1: Antorbital and infraorbitals 1−4 large, partial cylinders with slender osseous arches ([14]- character 1, Fig. 2; [18]-character 17).
98. Infraorbital canal tube. 0: Canal bearing infraorbital bones present as six separate ossifications. 1: Infraorbital canal a single, lightly ossified continuous tube.
99. First infraorbital. 0: Anterior bone of infraorbital laterosensory canal present as a dermal ossification anterior to first infraorbital and dorsal to maxilla. 1: First infraorbital not present as a separate ossification.
100. Mandibular canal. 0: Canal bearing bones of preopercular–mandibular laterosensory canal long and slender ossifications embedded in dermis; diameter of canal slender. 1: Preopercular–mandibular laterosensory canal bones large and fused with mandible; diameter of canal wide ([30]).
101. Mandibular canal ossicles. 0: Canal bearing bones of mandibular laterosensory canal long and slender tubes. 1: Canal bearing bones of mandibular laterosensory canal ossified as short, broad, dumbbell-shaped ossicles.
102. Supratemporal lateralis canal. 0: Supratemporal laterosensory canal straight, extending dorsally onto posterior portion of parietal; terminal pore oriented dorsoposteriorly; epidermis overlying supratemporal canal indistinguishable from general epidermis. 1: Supratemporal laterosensory canal curved at a sharp angle on surface of parietal, extending posterior onto epaxial surface of body; terminal canal pore oriented posteriorly; epidermis overlying supratemporal canal depigmented, forming a pale inverted L shaped patch ([11]).
103. Intercalar. 0: Intercalar present on surface of endochondral cranium in region where pterotic, epioccipital, and exoccipitals meet. 1: Intercalar absent ([13], character 13).
104. Olfactory bulb. 0: Olfactory bulb sessile, positioned directly ventral to anterior pole of forebrain; olfactory tract shorter length of olfactory lobe. 1: Olfactory bulb remote from rest of forebrain; olfactory tract longer than length of olfactory lobe ([13]-character 123).
105. Eyeball extrinsic muscles. 0: Extrinsic eyeball muscles and fibers of nervous innervation robust, their diameters greater than that of *in situ* collagen fibers. 1: Extrinsic eyeball muscles and innervating nerves small or absent, their diameters about the same as collagen fibers ([17]).
106. Accessory optic system. 0: Accessory optic tract large, easily visible in histological sections; neurons of tract organized into a distinct tegmental cell cluster (i.e., accessory optic nucleus). 1: Accessory optic tract reduced or absent; discrete accessory optic nucleus not visible in sections ([32], [33]).
107. Integumental taste buds. 0: Taste buds present on head in characiforms, and over entire integumental surface in siluriforms; diameters of nerves V and VII equal to or larger than that of other cranial nerves in isthmal region; primary facial and vagal sensory nuclei larger than medial octaval nucleus. 1: Taste buds entirely absent from extra-oral integument; nerves V and VII smaller than other cranial nerves of isthmal region; primary facial and vagal sensory nuclei smaller than medial octaval nucleus.
108. Schreckstoff/club cells. 0: Schreckstoff (alarm substance), club cells, and fright response present in Ostariophysi. 1: Schreckstoff, club cells, and fright response absent ([13]-character 117).
109. Passive electroreception. 0: No ability to detect weak ambient electric fields. 1: Structures and behavioral capacity to detect weak low frequency ambient electric fields, used in predation; associated neural structures in peripheral (e.g., ampullary electroreceptor organs) and central (e.g., electrosensory lateral line lobe, nucleus electrosensorius) nervous systems [34], [35].
110. Ampullary organ rosettes. 0: Ampullary organs distributed individually in integument. 1: Ampullary organs clustered in rosettes [36].
111. Active electroreception. 0: Passive, low frequency electroreception, used in predation; neural apparatus for detecting low frequency electric currents. 1: Electrogeneration and high frequency electroreception, used in communication and navigation (in addition to predation); neural apparatus for producing and detecting high frequency electric currents [37], [38].
112. Tuberous electroreceptors. 0: One class of tuberous electroreceptor organs. 1: Two classes of morphologically distinct tuberous electroreceptor organs [39].
113. Preotic lateralis ganglia. 0: All preotic lateral line nerve ganglia form from separate placodes, their axonal bundles entering brain separately. 1: Anterodorsal, anteroventral, and preopercular–mandibular lateral line nerve ganglia fused during ontogeny, their axons entering brain in a single bundle [17].
114. Posterior lateral line nerve. 0: Posterior lateral line nerve with no accessory rami. 1: Posterior lateral line nerve with dorsal ramus [24].
115. Lateral line afferents. 0: Lateral line afferents from electrosensory periphery intermingled as they course into the electrosensory lateral line lobe (ELL); fibers from different lateral line nerves not segregated. 1: Lateral line afferents fasciculated into discrete bundles; fibers from each lateral line nerve segregated from those of other lateral line nerves [40].
116. Anterior extent of eminentia granularis. 0: Eminentia granularis (EG) of dorsal medulla well developed, extending to posterior pole of optic tectum. 1: EG small, its anterior margin not extending to contact optic tectum [17].
117. Posterior EG. 0: Posterior margin of EG not extending to posterior margin of ELL. 1: Posterior lobe of EG well developed, wrapped around caudal lobe of cerebellum, its posterior margin extending to a vertical with posterior margin of ELL [17].
118. Anterior corpus cerebellum. 0: Anterior lobe of corpus cerebellum large, extending anterior to midlength of optic tectum; cerebellum overlying commissure of optic tectum. 1: Anterior lobe of corpus cerebellum extending to midlength of optic tectum; commissure of optic tectum exposed on dorsal surface.
119. Pacemaker nucleus. 0: Pacemaker nucleus of medulla oblongata small, positioned on midline of neuraxis, adjacent to medial longitudinal fasciculus; its ventral margin not contacting ventral aspect of medulla. 1: Pacemaker nucleus large, visible as a median, ovoid eminence on ventral surface of medulla; its ventral margin extending to medullary surface [41].
120. Palatines. 0: Autopalatine totally or partially ossified, straight. 1: Autopalatine unossified, arched.
121. Ectopterygoid. 0: Ectopterygoid ossified as a dentigerous element in membrane overlying ventral portion of endopterygoid. 1: Ectopterygoid and associated teeth absent ([13]-character 26).
122. Endopterygoid ascending process. 0: Lateral surface of endopterygoid smooth; no ascending process ossified in pterygocranial ligament (connecting endopterygoid with neurocranium). 1: Ascending process on lateral surface of endopterygoid; pterygocranial ligament ossified; base of ascending process situated approximately dorsal to articulation of quadrate with anguloarticular [26].
123. Endopterygoid ascending process. 0: Ascending process of endopterygoid developed in juvenile stages of growth and retained into adult. 1: Small ascending process of endopterygoid in juveniles obliterated by growth along dorsal margin of bone; no endopterygoid process in adults.
124. Endopterygoid anterior process. 0: Dorsal portion of pterygocranial ligament not ossified; base of ascending process of endopterygoid broader than its tip. 1: Entire extent of ligament ossified, forming a bony strut anterior to orbit; process equally as wide along most of its length
125. Mesopterygoid dentition. 0: Numerous small teeth distributed in an irregular field on anterior portion of ventral surface of endopterygoid. 1: Few or no teeth on endopterygoid ([26], [18]-character 22).
126. Metapterygoid posterior margin. 0: Posterior border of metapterygoid separated from hyomandibula by an unossified gap or with a cartilaginous margin. 1: Posterior margin of metapterygoid directly abutting hyomandibula ([13]-character 31).
127. Metapterygoid shape. 0: Metapterygoid shaped like head of a double-headed ax; dorsal and ventral margins concave. 1: Metapterygoid triangular in lateral view ([13]-Figs. 8–12).
128. Metapterygoid posterior wing. 0: Metapterygoid broad, its width at midlength greater than its total length. 1: Metapterygoid elongate and narrow, longer than wide at its midlength ([26], [27]
129. Size of symplectic. 0: Length of symplectic less than hyomandibula. 1: Length of symplectic greater than hyomandibula [26].
130. Orientation of hyomandibula. 0: Main axis of hyomandibula oblique to main axis of neurocranium. 1: Main axis of hyomandibula oriented horizontally, parallel to main axis of neurocranium ([26]- Fig. 31).
131. Hyomandibular articulation. 0: Proximal portion of hyomandibula broad; articulating surface facing anterodorsally. 1: Proximal portion of hyomandibula narrow; articulating surface facing dorsally ([26]-Fig. 31).
132. Dorsal margin of quadrate. 0: Dorsal margin of quadrate convex. 1: Dorsal margin of quadrate concave [24].
133. Mandibular canal size. 0: Mandibular canal ossicles long slender tubes. 1: Mandibular canal ossicles dumbbell-shaped [42].
134. Preopercular orientation 0: Long axis of preopercle oriented at an oblique angle to main axis of neurocranium. 1: Long axis of preopercle horizontal, roughly parallel with main axis of neurocranium [26], [27].
135. Preopercular pores. 0: One pore at dorsoposterior corner of preoperculum.1: Two pores at dorsoposterior corner of preopercle [20].
136. Anterior limb preoperculum. 0: Preopercle broad, crescent-shaped; ventral margin of anterior limb of preopercle curving smoothly to anterior tip. 1: Preopercle narrow, curved; ventral margin of anterior limb not ossified [13], [26].
137. Shape of opercle. 0: Outline of opercle approximately rectangular; dorsal margin shorter than posterior margin, and interrupted by a pronounced angle. 1: Opercle approximately triangular; dorsal margin about as long as posterior margin, and either slightly curved or straight ([13]-character 36).
138. Opercular dorsal margin. 0: Dorsal margin of opercle convex. 1: Dorsal margin of opercle straight. 2: Dorsal margin of opercle concave ([13], [43].
139. Branchiostegal rays. 0: 3–4 rays. 1: 5–6 rays. 2: more than 7 rays.
140. Branchiostegal ray morphology. 0: Anterior 1–2 rays broad. 1: Anterior rays narrow.
141. Gill raker configuration. 0: Gill rakers directly attached to gill arches. 1: Base of gill rakers not mineralized, rakers (when present) not attached to gill arches (Mago-Leccia, 1978 [30,14]-character 24).
142. Gill raker tips. 0: Gill rakers ossified to distal tips. 1: Distal tips of gill rakers cartilaginous ([30], [14]-character 24).
143. Anterior pharyngobranchial. 0: Anterior pharyngobranchial (associated with gill arch two) ossified, articulating with parasphenoid ([13]-Fig. 13). 1: Anterior pharyngobranchial unossified ([27]-character 40).
144. Pharyngobranchials. 0: Pharyngobranchials of third and fourth arches cartilaginous. 1: Pharyngobranchials of third and fourth arches ossified.
145. Pharyngobranchial plates. 0: Four dentigerous plates present on posterior gill arches. 1: One dentigerous plate present on posterior gill arch ([13]-character 51).
146. Epibranchial 4. 0: Posterior margin of fourth epibranchial flat. 1: Fourth epibranchial with short posterior process.
147. Epibranchial 3. 0: Third epibranchial straight. 1: Third epibranchial sinuous.
148. Shape of 4th epibranchial. 0: Fourth epibranchial with short ascending process. 1: Fourth epibranchial with an elongate ascending process.
149. Epibranchial 5. 0: Posterior surface of fifth epibranchial flat. 1: Fifth epibranchial with posterior process.
150. Epibranchial 5 post-med. process 0: Posterior surface of seventh epibranchial with a dorsomedially oriented process. 1: Posterior surface of seventh epibranchial with a dorsoventrally oriented process ([27]-character 42b, Figs. 18–20).
151. Ceratobranchial 2. “Posterior process of fourth ceratobranchial” (Character 156) of Albert, 2001. 0: Posterior surface of second ceratobranchial smooth. 1: Posterior surface of second ceratobranchial with a medially oriented process ([27]-character 39, Figs. 17–19).
152. Ceratobranchial 4. “Lateral process of sixth ceratobranchial” (Character 157) in Albert, 2001. 0: Lateral surface of fourth ceratobranchial smooth. 1: Lateral surface of fourth ceratobranchial with an anterolaterally oriented process ([27]-character 38, Figs. 17–19).
153. Hypobranchial 1. 0: First hypobranchial rectangular in dorsal view; anterior margin straight. 1: First hypobranchial triangular in dorsal view. 2: First hypobranchial rounded or pentagonal in dorsal view; anterior margin interrupted by a sharp angle ([27]-character 33b).
154. Hypobranchial 2. 0: Medial surface of second hypobranchial flat; anterior tip symmetrically conical or flat. 1: Anterior tip of second hypobranchial with a large medially oriented process, contacting contralateral third hypobranchial across midline by means of a cartilaginous bridge ([27]-character 34a).
155. Hypobranchial teeth. 0: Eight or more teeth present on sixth hypobranchial. 1: Seven or fewer teeth present on sixth hypobranchial [31].
156. Basihyal dorsal ridge. 0: Dorsal surface of basihyal flat or rounded. 1: Dorsal surface of basihyal convex along its long axis, forming a ridge ([27]-character 29).
157. Basihyal dorsal groove. 0: Dorsal surface of basihyal flat or convex. 1: Dorsal surface of basihyal concave along its long axis, forming a shallow trough ([27]-character 30a).
158. Basibranchials. 0: All five basibranchial elements (including basihyal) ossified. 1: All five elements of basibranchial series (including basihyal) unossified.
159. Basibranchial one. 0: First (anterior) basibranchial elongate, width at midlength about same as at anterior and posterior ends. 1: First basibranchial foreshortened and broad, hourglass shaped, breadth at midlength narrower than at either end
160. Urohyal head. 0: Anterior head of urohyal narrow, lateral surfaces flat. 1: Anterior head of urohyal large, with lateral ridges [44].
161. Urohyal blade. 0: Posterior blade of urohyal ossified, extending posterior to fourth basibranchial. 1: Posterior blade of urohyal unossified, anterior head of urohyal positioned ventral to second basibranchial.
162. Urohyal blade hyperossified. 0: Urohyal blade short, ossified to level of third basibranchial. 1: Urohyal blade long, ossified to level of fourth basibranchial.
163. Posttemporal. 0: Posttemporal independent from supracleithrum in mature specimens. 1: Posttemporal fused with supracleithrum in mature specimens ( [14]-character 10).
164. Scapular foramen. 0: Unossified area along medial margin in scapulocoracoid cartilage separating coracoid and scapular ossifications. 1: Unossified region of scapulocoracoid cartilage included entirely within the scapula, forming a large foramen ([14]-character 9).
165. Mesocoracoid. 0: Mesocoracoid ossified within scapulocoracoid cartilage, forming a bridge between medial surface of coracoid and cleithrum. 1: Mesocoracoid not ossified [16].
166. Anterior coracoid process. 0: Anterior coracoid process extending anterior towards cleithral symphysis, paralleling ventral margin of cleithrum. 1: Anterior coracoid process not extending to a vertical with contact of dorsomedial limb of coracoid with cleithrum.
167. Proximal pectoral radials. 0: Proximal radials three and four separate. 1: Proximal radials three and four co-ossified in adult specimens ([14]- character 15, Fig. 7).
168. Pectoral fin. 0: Pectoral fin large, more than 43% head length; 1: Pectoral fin, less than 43% head length ([18]-character 29).
169. Pelvic girdle and fin. 0: Pelvic girdles and fins present. 1: No pelvic girdles or fins ([13]-character 103).
170. Claustrum. 0: Dissociated dorsomedial portion of first neural arch modified to form claustrum. 1: Claustrum absent as an ossified element ([13]-character 67).
171. *Os suspensorium*. 0: Anterior ramus long, reaching third vertebra. 1: Anterior ramus short, reaching second vertebra.
172. Anterior vertebrae. 0: Close proximity between parapophyses of second vertebrae and *os suspensorium*. 1: Parapophyses of second vertebrae separated by distinct gap from the *os suspensorium* (modified from [14]-character 8; [13]-characters 74 and 92; [18]-character 39).
173. Position of neural spines. 0: Neural spines inserting on middle of caudal vertebral centra. 1: Neural spine inserting on posterior margin of caudal vertebral centra.
174. Vertebral fenestrae. 0: Lateral walls of neural arches completely ossified; dorsal margin straight. 1: Lateral walls of neural arches with several small fenestrae; dorsal margin uneven, with several evaginations [45], [46].
175. Shape anterior intermuscular bones. 0: Intermusculars simple with little branching. 1: Intermusculars highly branched [47].
176. Caudal intermusculars. 0: Inability to regenerate intermuscular bones. 1: Capacity to regenerate ossified intermuscular bones ([45], [46]- character 32).
177. Displaced hemal spines (DHS). 0: All hemal spines medial, fused with hemal arches in adult specimens; one to one correspondence between caudal vertebrae and associated hemal spines. 1: Three additional hemal spines positioned in hypaxial musculature posterior to body cavity, often lateral to unmodified hemal spines, rarely fused with hemal arches or parapophyses; irregular association with posterior thoracic and anterior caudal vertebrae.
178. DHS anterior series. 0: Three DHSs in hypaxial musculature immediately posterior to body cavity. 1: Anterior series of 8–14 DHSs in hypaxial musculature lateral to body cavity.
179. DHS 1. 0: Anterior DHS approximately as straight and as wide as other hemal spines. 1: Anterior DHS large, two to three times as broad as other hemal spines, often exhibiting additional distal tips. In the derived state the anterior DHS is curved and scythe shaped.
180. DHS 1 proximal surface. 0: Proximal surface of first DHS narrower than descending blade. 1: Proximal surface of first DHS broad as blade.
181. DHS 2 shape. 0: Second posterior DHS straight. 1: Second posterior DHS curved [14].
182. Number posterior DHS. 0: Two or three DHSs posterior to large anterior spine. 1: A single DHS posterior to large anterior spine.
183. Dorsal organ. 0: Posterodorsal margin of body without a longitudinal fleshy organ. 1: Posterodorsal margin of body with a median flap or bar of fleshy tissue, extending parallel to the dorsal margin of epaxial musculature [48].
184. Dorsal organ length. 0: Dorsal organ extending along dorsal margin posterior to midlength of body. 1: Dorsal organ extends along entire dorsal margin of body, from nape to caudal peduncle ([48], Albert, unpubl. obs.).
185. Dorsal fin. 0: Dorsal fin present. 1: Dorsal fin absent [13].
186. Adipose fin. 0: Adipose fin present. 1: Adipose fin absent [13].
187. Anal fin origin. 0: Anal-fin origin posterior to cleithrum of pectoral girdle. 1: Anal-fin origin ventral to posterior margin of cleithrum. 2: Anal fin origin near branchial isthmus.
188. Number anal-fin rays. 0: Anal fin short, extending less than 0.2 times total length of body; fewer than 20 rays. 1: Anal fin long, extending along majority of ventral body margin; 100–159 rays. 2: 160–199 rays. 3: 200–299 rays. 4: 300 or more rays. Taxa coded by modal number of anal-fin rays.
189. Anal-fin rays unbranched. 0: 10–15 anal-fin rays branched into two rami about half distance to their tips. 1: Anterior 15–25 rays anal-fin rays unbranched to their tips (modified from [18]-character 49). 2: 30–60 unbranched anal-fin rays. 3: all anal-fin rays unbranched.
190. Anal-fin pterygiophore (AFP) length. 0: Anal-fin pterygiophores shorter than hemal spines at midbody; less than one-third total body depth (more than 1.5 times into depth of axial musculature). 1: Anal-fin pterygiophores longer than hemal spines at midbody; more than one-third total body depth (less than 1.5 times into depth of axial musculature).
191. Shape of AFP blades. 0: Descending blades of proximal anal-fin pterygiophores slender, approximately cylindrical in cross section. 1: Descending blades of anal-fin pterygiophores broad, anterior and posterior margins extending into ventral median septum in cross section.
192. Shape of AFP tips. 0: Anal-fin pterygiophores tapering smoothly to tips. 1: Tips of pterygiophores shaped like an arrow-head; axial series of pterygiophores providing the ventral margin of the anal-fin base a scalloped appearance [43].
193. Anal-fin ray articulation. 0: Anal-fin rays articulate with distal anal-fin pterygiophores. 1: Anal-fin rays articulate with proximal anal-fin pterygiophores ([13]-character 107).
194. Distal AFP. 0: Distal anal-fin pterygiophores present. 1: No distal anal-fin pterygiophores.
195. Free neural and hemal spines. 0: No capacity to regenerate axial structures. 1: Capacity to generate series of free neural and hemal spines associated with regenerated cartilaginous rod.
196. Body cavity long. 0: Body cavity associated with 16–19 precaudal vertebrae (including Weberian ossicles). 1: Body cavity associated with 23–29 precaudal vertebrae. 2: Body cavity associated with 30–39 precaudal vertebrae. 3: Body cavity associated with 40 or more precaudal vertebrae.
197. Body cavity short. 0: Body cavity associated with 16–19 vertebrae. 1: Body cavity short; associated with 12–15 precaudal vertebrae. 2: Body cavity very short; associated with 11 or fewer precaudal vertebrae ([11], [18]-character 41).
198. Hemal spines. 0: Hemal spines present; body cavity associated with 16–19 vertebrae lacking hemal spines, and 8–10 vertebrae with paired ribs; caudal (post-coelomic) vertebrae bearing hemal spines present. 1: Hemal spines absent, body cavity extending almost to tip of the tail; no caudal (post-coelomic) vertebrae.
199. Number of pleural ribs. 0: Eight or more pairs of pleural ribs. 1: Seven or fewer pairs of pleural ribs ([14]- characters 5 and 13).
200. Length of anterior ribs. 0: Anterior two or three ribs relatively short, their lengths less than 80% body depth at pectoral girdle. 1: Length of anterior two ribs greater than 80% body depth at pectoral girdle ([14]- character 6).
201. Size of anterior ribs. 0: Anterior pair of pleural ribs narrow; breadth approximately equal to width. 1: Anterior ribs broad, breadth two to three times width.
202. Posterior parapophyses. 0: Parapophyses of posterior precaudal vertebra small, their ventral margins oblique to long axis of body, not contacting one another along midline. 1: Parapophyses of posterior precaudal vertebra longer than wide, their ventral margins parallel with long axis of body, abutting at midline.
203. Shape last precaudal parapophyses. 0: Parapophyses of last precaudal vertebra broad and triangular, their tips rounded. 1: Parapophyses of last precaudal vertebra slender and sinuous, their tips pointed.
204. Post. chamber gas bladder. 0: Gas bladder divided into two unequal chambers; anterior chamber larger in diameter and shorter in length than posterior chamber. 1: Posterior chamber of gas bladder elongate, passing between hemal arches of postcoelomic axial skeleton and musculature ([18]- character 46). 2: Posterior chamber of gas bladder extending to tip of tail.
205. Gas bladder. 0: Anterior and posterior chambers of gas bladder thin and translucent. 1: Anterior chamber of gas bladder encapsulated in a thick, opaque layer of tissue.
206. Anal position. 0: Position of anus relatively fixed during post-larval development; anus located posterior to tip of pectoral fin. 1: Position of anus changing allometrically during ontogeny, starting near posterior end of coelomic cavity and growing anterior to pectoral girdle; anus located near isthmus.
207. Anal-fin base. 0: Anal-fin base naked. 1: Anal-fin base fleshy [24].
208. Urogenital papilla. 0: Urogenital pore sessile, opening flush with ventral margin of body wall in sexually mature specimens. 1: Urogenital pore elevated onto a papilla in sexually mature specimens [11].
209. Epidermal laterosensory canals. 0: Epidermal laterosensory canals absent on posterior body. 1: Epidermal laterosensory canals present on posterior body [24].
210. Tail length. 0: Length of tail posterior to anal-fin 17–45% total length. 1: Tail short, 0–16% total length. 2: Tail long, more than 45% total length.
211. Elongate caudal rod. 0: Caudal fin present with hypural plate and segmented rays. 1: cartilaginous bar or rod, regenerated in place of caudal vertebrae ([13]-character 109; [10]-character 222 in part)
212. Caudal appendage. 0: Caudal appendage of similar length in adult males and females. 1: Caudal appendage elongate in sexually mature males ([24]).
213. Caudal fin. 0: Caudal fin present. 1: Caudal fin absent (adapted from [10]-character 222).
214. Electric organs (EO). 0: All axial muscle fibers unmodified; no organs capable of generating rhythmic electric discharges. 1: Paired electrogenic organs developing in larval hypaxial musculature; electric organ composed of rows of modified elongate myofibrils (electrocytes; [49,13]- character 121).
215. Number of hypaxial EO. 0: Single hypaxial electric organ. 1: Three anatomically distinct hypaxial electric organs (i.e. Sachs׳, Hunter׳s, and Main electric organs).
216. Main EO electrocyte morphology. 0: Electrocytes cigar shaped, elongate; longitudinal axis parallel with neuraxis. 1: Electrocytes barrel shaped, cylindrical; long axis oriented vertically. 2: Electrocytes coin-shaped (new character state).
217. Hypaxial EO ontogeny. 0: Main electric organ of mature specimens developing from a medial portion of hypaxial musculature, extending along ventral margin of hypaxial musculature. 1: Hypaxial electric organ replaced during development, adult organ not derived from hypaxial musculature [50].
218. Mental accessory EO. 0: absent. 1: present [24].
219. Mental accessory EO configuration. 0: Mental accessory organ absent or short with few electrocytes. 1: Mental accessory EO long, threadlike with many electrocytes [24].
220. Humeral accessory EO. 0: No humeral electric organ. 1: Humeral electric organ extending dorsally from pectoral fin base, and then posteriorly along horizontal myoseptum a distance less than length of pectoral fin [51].
221. Neural EO. 0: Main electric organ of mature specimens ontogenetically derived from hypaxial musculature. 1: Main electric organ of mature specimens derived from electromotor neurons which innervate larval hypaxial organ [50], [52].
222. EOD form. 0: EOD of mature specimens produced as discrete non-overlapping pulses with alternating periods of current flow and no current flow; capacity for EOD frequency modulations present; cells of pacemaker nucleus organized into two separate clusters. 1: EOD produced as a continual series of discharges to form a quasi-sinusoidal pattern of current emission; no capacity for EOD frequency modulations; relay and pacemaker cells mingled in a single medullary nucleus [53].
223. EOD monophasic in adults. 0: EOD of mature specimens with two (sometimes three or four) phases; EOD characterized by both head-positive and head-negative depolarizations. 1: Monophasic EOD of juveniles retained into maturity; EOD characterized exclusively by head-positive depolarizations. 2: Monophasic hyperpolarization from negative baseline [49], [54].

## 4. Model-based total evidence (MBTE) analyses

### 4.1. Maximum-likelihood (ML)

MBTE-ML analyses datasets were conducted in [62] using a supermatrix of concatenated molecular and morphological data. Models of nucleotide evolution were estimated in PartitionFinder v.1.1.1 [63]. *Mkv* model [64] was used for the morphological dataset. MBTE-ML analyses consisted of two independent runs, each one starting from a BioNJ starting tree and using the Subtree Pruning and Regrafting (SPR) algorithm to search for tree improvement in terms of likelihood scores. All other parameters were set as default. To assess node support, 100 non-parametric bootstrap replications were performed for each independent tree search resulting in a total of 200 pseudo-replicates. A consensus tree with bootstraps was computed using the function SumTrees from DendroPy 3.7.0 [65]. Computer scripts to replicate analyses are shown in Supplementary 1.

### 4.2. Bayesian inference (BI)

MBTE-BI analyses were conducted in MrBayes 3.2 [66] using a supermatrix of concatenated molecular and morphological data. Models of nucleotide evolution were estimated in PartitionFinder v.1.1.1 [63]. *Mkv* model [64] was used for the morphological dataset. MBTE-BI analysis consisted of two runs (four chains each) of the Metropolis-Coupled Markov Chain Monte Carlo (MC^3^). Each run was comprised of 5.0×10^7^ generations with model parameter values and a single tree sampled every 5×10^3^ generation. All other parameters were set as default. To ensure adequate mixing of the MCMC, effective sample size values (ESS>200) were inspected for parameter estimates in Tracer 1.5. The two independent runs were summarized with “sump” and “sumt” commands in MrBayes 3.2 [66]. The initial 25% of sampled topologies were discarded as burn-in procedure. The remaining topologies were used to construct a 50% majority-rule consensus tree. Posterior probabilities were visualized in FigTree 1.4.0. Computer scripts to replicate analyses are shown in Supplementary 1.

## 5. Morphological synapomorphies

### 5.1. List of synapomorphies used to diagnose clades

Node 175: GYMNOTIFORMES

ch. 3-Body shape profile. “Body Depth,” character 2 in Albert, 2001. / 1: Body elongate, slender, depth less than 11% total length.

ch. 7-Gape short. / 1: Rictus extends to a vertical with mental symphysis, gape very small, less than twice diameter of eye, oriented oblique to long axis of head.

ch. 14-Position of eye. / 1: Eye completely covered by epidermis in adults; orbital margin not free.

ch. 72-Lateral ethmoid size. / 1: Lateral ethmoid reduced in size; four peripheral margins not contacting other bony surfaces.

ch. 84-Parasphenoid lateral process. / 1: Lateral margins of parasphenoid not extending to a horizontal with trigeminal foramen.

ch. 106-Accessory optic system. / 1: Accessory optic tract reduced or absent; discrete accessory optic nucleus not visible in sections.

ch. 107-Integumental taste buds. / 1: Taste buds entirely absent from extra-oral integument; nerves V and VII smaller than other cranial nerves of isthmal region; primary facial and vagal sensory nuclei smaller than medial octaval nucleus.

ch. 108-Schreckstoff/club cells. / 1: Schreckstoff, club cells, and fright response absent.

ch. 110-Ampullary organ rosettes. / 1: Ampullary organs clustered in rosettes.

ch. 111-Active electroreception. / 1: Electrogeneration and high frequency electroreception, used in communication and navigation (in addition to predation); neural apparatus for producing and detecting high frequency electric currents.

ch. 112-Tuberous electroreceptors. / 1: Two classes of morphologically distinct tuberous electroreceptor organs.

ch. 120-Palatines. / 1: Autopalatine unossified, arched.

ch. 121-Ectopterygoid. / 1: Ectopterygoid and associated teeth absent.

ch. 125-Mesopterygoid dentition. / 1: Few or no teeth on endopterygoid.

ch. 127-Metapterygoid shape. / 1: Metapterygoid triangular in lateral view.

ch. 141-Gill raker configuration. / 1: Base of gill rakers not mineralized, rakers (when present) not attached to gill arches.

ch. 148-Shape of 4th epibranchial. / 1: Fourth epibranchial with an elongate ascending process.

ch. 165-Mesocoracoid. / 1: Mesocoracoid not ossified.

ch. 169-Pelvic girdle and fin. / 1: No pelvic girdles or fins.

ch. 170-Claustrum. / 1: Claustrum absent as an ossified element.

ch. 185-Dorsal fin. / 1: Dorsal fin absent.

ch. 186-Adipose fin. / 1: Adipose fin absent.

ch. 188-Number anal-fin rays. / 2: 160–199 rays.

ch. 193-Anal-fin ray articulation. / 1: Anal-fin rays articulate with proximal anal-fin pterygiophores.

ch. 194-Distal AFP. / 1: No distal anal-fin pterygiophores.

ch. 213-Caudal fin. / 1: Caudal fin absent.

ch. 214-Electric organs (EO). / 1: Paired electrogenic organs developing in larval hypaxial musculature; electric organ composed of rows of modified elongate myofibrils.

ch. 215-Number of hypaxial EO. / 1: Three anatomically distinct hypaxial electric organs (i.e. Sachs׳, Hunter׳s, and Main electric organs).

Node 176: GYMNOTIDAE clade

ch. 8-Oral opening in adults. / 0: Upper and lower jaws of equal length, oral aperture terminal.

ch. 64-Mesethmoid, tip size. / 1: Portion of mesethmoid anterior to ventral ethmoid flexed ventrally in mature specimens; its dorsal surface anterior and posterior to ventral ethmoid at an oblique angle; its ventral surface oblique to dorsal surface. terminal.

ch. 74-Base lateral ethmoid. / 1: Lateral ethmoid narrow or tubular; length of its base less than one-third length of its anterior margin.

ch. 79-Cranial fontanels. / 1: Frontals in contact with each other along the entire extent of their medial margins in mature specimens.

ch. 140-Branchiostegal ray morphology. / 0: Anterior 1–2 rays broad.

ch. 163-Posttemporal. / 0: Posttemporal independent from supracleithrum in mature specimens.

ch. 196-Body cavity long. / 2: Body cavity associated with 30–39 precaudal vertebrae.

ch. 204-Post. chamber gas bladder. / 1: Posterior chamber of gas bladder elongate, passing between hemal arches of postcoelomic axial skeleton and musculature.

ch. 210-Tail length. / 1: Tail short, 0–16% total length.

Node 177: *Gymnotus* clade

ch. 8-Oral opening in adults. / 1: Lower jaw extends anterior to upper, oral aperture superior.

ch. 10-Position of anterior nares. / 1: Anterior nares located very close to or within gape, narial opening oriented anteroventrally.

ch. 11-Anterior narial pore. / 1: Anterior narial opening sessile, its rim flush with surrounding integument.

ch. 33-Lateral line pores. / 1: Posterior lateral line pores tubular; tube length more than three times pore diameter.

ch. 34-Lateral line ventral rami. / 1: Numerous ventral rami extending parallel with lateral line.

ch. 37-Tooth shape. / 1: Teeth in both jaws villiform, each tooth a long cylindrical shaft with a narrow base.

ch. 47-Maxilla descending blade. / 2: Anteroventral margin of descending blade not ossified; distal half of blade extending as a narrow process with a sharp point at its distal tip.

ch. 66-Ventral ethmoid lateral process. / 1: No lateral process of ventral ethmoid; ventral ethmoid not contacting lateral ethmoid cartilage.

ch. 67-Ventral ethmoid lateral process shape. / 1: Lateral process of ventral ethmoid robust, posterior surface forming articulation with lateral ethmoid cartilage broad and rounded, covered by a cartilage cap.

ch. 68-Median septum of ventral ethmoid. / 1: Ossified median septum of ventral ethmoid elongate in mature specimens, longer than deep, extending posterior to posterior margin of lateral process.

ch. 71-Ethmoid cartilage. / 1: Ethmoid cartilage deeper than long; antorbital region of snout about as deep as long.

ch. 87-Parasphenoid process. / 1: Parasphenoid with anteroventral process.

ch. 91-Nasal loop. / 1: Commissure between infraorbital and supraorbital canals extended anteriorly, forming a loop ventrolateral to nasal capsule; antorbital and first infraorbital bones situated near anterior nares.

ch. 92-Infraorbital subnasal extension. / 1: Anterior extension of infraorbital canal shorter than width of canal pore; anterior canal pore of infraorbital canal situated near first infraorbital.

ch. 122-Endopterygoid ascending process. / 1: Ascending process on lateral surface of endopterygoid; pterygocranial ligament ossified; base of ascending process situated approximately dorsal to articulation of quadrate with anguloarticular.

ch. 143-Anterior pharyngobranchial. / 1: Anterior pharyngobranchial unossified.

ch. 158-Basibranchials. / 1: All five elements of basibranchial series (including basihyal) unossified.

ch. 195-Free neural and hemal spines. / 1: Capacity to generate series of free neural and hemal spines associated with regenerated cartilaginous rod.

ch. 201-Size of anterior ribs. / 1: Anterior ribs broad, breadth two to three times width.

Node 178: *Gymnotus pantherinus* clade

No diagnostic character in matrix.

Node 180: *Gymnotus coatesi*+*G. anguillaris*+*G. tigre*+*G. cylindricus*+G. *carapo* clades

ch. 16-Oblique pigment bands. / 1: Multiple (13–50) pale bands with straight margins of alternating high and low melanophore density along lateral surface of body, oriented at an oblique angle to longitudinal body axis. Bands sometimes interrupted by patches of depigmented integument, resulting in a distribution of blotches arranged in oblique bands along the lateral surface of body.

ch. 35-No. ventral rami. / 1: Median 15 or more.

Node 181: *Gymnotus coatesi* clade

No diagnostic character in matrix. See Maxime (2014).

Node 187: *Gymnotus anguillaris*+*G. tigre*+*G. cylindricus*+*G. carapo* clade

ch. 188-Number anal-fin rays. / 3: 200–299 rays. Taxa coded by modal number of anal-fin rays.

ch. 196-Body cavity long. / 3: Body cavity associated with 40 or more precaudal vertebrae.

Node 188: *Gymnotus cataniapo* clade

No diagnostic character in matrix.

Node 190: *Gymnotus tigre*+*G. cylindricus species groups*+*G. carapo* clade

ch. 16-Oblique pigment bands. / 2: Oblique pigment bands along longitudinal axis with wavy margins.

ch. 37-Tooth shape. / 0: Teeth in both jaws conical, with a broad base tapering toward the cusp.

ch. 135-Preopercular pores. / 1: Two pores at dorsoposterior corner of preopercle.

ch. 165-Mesocoracoid. / 0: Mesocoracoid ossified within scapulocoracoid cartilage, forming a bridge between medial surface of coracoid and cleithrum.

ch. 190-Anal-fin pterygiophore (AFP) length. / 1: Anal-fin pterygiophores longer than hemal spines at midbody; more than one-third total body depth (less than 1.5 times into depth of axial musculature).

ch. 201-Size of anterior ribs. / 0: Anterior pair of pleural ribs narrow; breadth approximately equal to width.

Node 191: *Gymnotus tigre* clade

ch. 15-Anal fin membrane. / 2: Anal fin membrane striped.

ch. 38-Tooth tip shape. / 1: Tips of teeth directed anteriorly (recurved).

Node 192: *Gymnotus cylindricus*+*G. carapo* clade

ch. 196-Body cavity long. / ch.196-Body cavity long. / 2: Body cavity associated with 30–39 precaudal vertebrae.

Node 193: *Gymnotus cylindricus* clade

ch. 16-Oblique pigment bands. / 0: Body pigmentation evenly distributed along longitudinal axis.

ch. 35-No. ventral rami. / 0: Median 14 or less.

ch. 135-Preopercular pores. / 0: One pore at dorsoposterior corner of preoperculum.

ch. 188-Number anal-fin rays. / 2: 160–199 rays.

ch. 190-Anal-fin pterygiophore (AFP) length. / 0: Anal-fin pterygiophores shorter than hemal spines at midbody; less than one-third total body depth (more than 1.5 times into depth of axial musculature).

Node 196: *G. carapo* clade

ch. 15-Anal fin membrane. / 1: With pale posterior patch.

ch. 37-Tooth shape. / 2: Teeth with triangular, arrow-head shape.

Node 201: *Gymnotus carapo* species-complex clade

ch. 1-Body shape 1. / 0: Body laterally compressed, body width at pectoral fin base less than 70% its depth.

ch. 3-Body shape profile. / 0: Body relatively deep in profile, depth at pectoral girdle more than 11% total length.

ch. 157-Basihyal dorsal groove. / 1: Dorsal surface of basihyal concave along its long axis, forming a shallow trough.

ch. 166-Anterior coracoid process. / 1: Anterior coracoid process not extending to a vertical with contact of dorsomedial limb of coracoid with cleithrum.

Node 216: STERNOPYGOIDEI clade

ch. 1-Body shape 1. / 0: Body laterally compressed, body width at pectoral fin base less than 70% its depth.

ch. 27-Branchial opening. / 1: Vertical extent of branchial opening restricted to region around pectoral fin base; ventral portion reduced by a dorsolateral continuation of epidermis from isthmus.

ch. 28-Pseudotympanum. / 1: Sixth vertebra not covered by superior oblique.

ch. 39-Premaxilla size. / 1: Small. The anterodorsal orientation of the articular surface of the maxilla with the autopalatine is a consequence of the large size of the premaxilla and the associated posterior position of the maxilla.

ch. 62-Mesethmoid, tip size. / 1: Anterior tip of mesethmoid small, anterodorsal surface narrow, with a median knob-shaped process directed anteriorly between two small lateral processes.

ch. 63-Mesethmoid, tip shape. / 1: Portion of mesethmoid anterior to ventral ethmoid flexed ventrally in mature specimens; its dorsal surface anterior and posterior to ventral ethmoid at an oblique angle; its ventral surface oblique to dorsal surface.

ch. 115-Lateral line afferents. / 1: Lateral line afferents fasciculated into discrete bundles; fibers from each lateral line nerve segregated from those of other lateral line nerves.

ch. 139-Branchiostegal rays. / 1: 5–6 rays.

ch. 160-Urohyal head. / 1: Anterior head of urohyal large, with lateral ridges.

ch. 177-Displaced hemal spines (DHS). / 1: Three additional hemal spines positioned in hypaxial musculature posterior to body cavity, often lateral to unmodified hemal spines, rarely fused with hemal arches or parapophyses; irregular association with posterior thoracic and anterior caudal vertebrae.

ch. 187-Anal fin origin. / 1: Anal-fin origin ventral to posterior margin of cleithrum.

ch. 197-Body cavity short. / 1: Body cavity short; associated with 12–15 precaudal vertebrae.

ch. 206-Anal position. / 1: Position of anus changing allometrically during ontogeny, starting near posterior end of coelomic cavity and growing anterior to pectoral girdle; anus located near isthmus.

ch. 211-Elongate caudal rod. / 1: cartilaginous bar or rod, regenerated in place of caudal vertebrae.

Node 217: RHAMPHICHTHYOIDEA clade

ch. 36-Adult dentition. / 1: Oral teeth present in juveniles, lost and not replaced during development.

ch. 66-Ventral ethmoid lateral process. / 1: No lateral process of ventral ethmoid; ventral ethmoid not contacting lateral ethmoid cartilage.

ch. 81-Orbitosphenoid shape. / 1: Anterior margin of orbitosphenoid not ossified, orbitosphenoid narrow, its ventral margin about as long or shorter than its dorsal margin.

ch. 87-Parasphenoid process. / 1: Parasphenoid with anteroventral process.

ch. 95-Antorbital size. / 1: Antorbital large; its ventral portion larger than maxilla; expanded dorsal portion contacts autopalatine.

ch. 98-Infraorbital canal tube. / 1: Infraorbital canal a single, lightly ossified continuous tube.

ch. 99-First infraorbital. / 1: First infraorbital not present as a separate ossification.

ch. 118-Anterior corpus cerebellum. / 0: Anterior lobe of corpus cerebellum large, extending anterior to midlength of optic tectum; cerebellum overlying commissure of optic tectum.

ch. 128-Metapterygoid posterior wing. / 1: Metapterygoid elongate and narrow, longer than wide at its midlength.

ch. 134-Preopercular orientation. / 1: Long axis of preopercle horizontal, roughly parallel with main axis of neurocranium.

ch. 137-Shape of opercle. / 0: Outline of opercle approximately rectangular; dorsal margin shorter than posterior margin, and interrupted by a pronounced angle.

ch. 181-DHS 2 shape. / 1: Second posterior DHS curved.

ch. 208-Urogenital papilla. / 1: Urogenital pore elevated onto a papilla in sexually mature specimens.

Node 218: Hypopomidae (*Akawaio*, *Hypopomus*, Microsternarchini, *Brachyhypopomus*) clade

ch. 1-Body shape 1. / 1: Body cylindrical or subcylindrical, roughly circular in cross section, body depth at pectoral girdle approximately equal to its width.

ch. 70-Dermal vomer. / 1: Dermal vomer not ossified.

ch. 96-Antorbital shape. / 1: Antorbital crescent-shaped.

ch. 114-Posterior lateral line nerve. / 1: Posterior lateral line nerve with dorsal ramus.

ch. 157-Basihyal dorsal groove. / 1: Dorsal surface of basihyal concave along its long axis, forming a shallow trough.

ch. 163-Posttemporal. / 0: Posttemporal independent from supracleithrum in mature specimens.

ch. 166-Anterior coracoid process. / 1: Anterior coracoid process not extending to a vertical with contact of dorsomedial limb of coracoid with cleithrum.

ch. 187-Anal fin origin. / 0: Anal-fin origin posterior to cleithrum of pectoral girdle. ch. 207-Anal-fin base. / 1: Anal-fin base fleshy.

ch. 209-Epidermal laterosensory canals. / 1: Epidermal laterosensory canals present on posterior body.

Node 219: *Hypopomus*+Microsternarchini+*Brachyhypopomus* clade

ch. 122-Endopterygoid ascending process. / 1: Ascending process on lateral surface of endopterygoid; pterygocranial ligament ossified; base of ascending process situated approximately dorsal to articulation of quadrate with anguloarticular.

ch. 167-Proximal pectoral radials. / 1: Proximal radials three and four co-ossified in adult specimens.

Node 220: *Hypopomus*+Microsternarchini clade

ch. 46-Maxilla descending blade. / 1: Descending blade of maxilla broad, connective tissue membrane along its anteroventral margin ossified to form a thin shelf; anterior portion of maxilla rhomboid in lateral view.

Node 221: Microsternarchini (*Procerusternarchus*, *Racenisia*, *Microsternarchus*) clade

ch. 30-Scales on middorsum. / 1: Scales absent from head, anterior portion of dorsal midline, and area dorsal to pectoral fins.

ch. 43-Anterior maxillary process. / 2: Anterior process of maxilla cartilaginous; ventral margin of descending blade extends to articulation of maxilla with autopalatine, forming anterior border of maxilla; maxilla crescent shaped in lateral view.

ch. 52-Dentary dorsal process. / 1: Dorsal process on posterior region of dentary. New character.

ch. 73-Lateral ethmoid. / 1: Lateral ethmoid not ossified.

ch. 122-Endopterygoid ascending process. / 0: Lateral surface of endopterygoid smooth; no ascending process ossified in pterygocranial ligament (connecting endopterygoid with neurocranium)

ch. 140-Branchiostegal ray morphology. / 0: Anterior 1–2 rays broad.

ch. 158-Basibranchials. / 1: All five elements of basibranchial series (including basihyal) unossified.

Node 222: *Procerusternarchus*+*Microsternarchus*

ch. 139-Branchiostegal rays. / 0: 3–4 rays.

Node 224: *Brachyhypopomus* clade

ch. 8-Oral opening in adults. / 0: Upper and lower jaws of equal length, oral aperture terminal.

ch. 74-Base lateral ethmoid. / 1: Lateral ethmoid narrow or tubular; length of its base less than one-third length of its anterior margin.

ch. 171-Os suspensorium. / 1: Anterior ramus short, reaching second vertebra.

ch. 188-Number anal-fin rays. / 3: 200–299 rays. Taxa coded by modal number of anal-fin rays.

ch. 212-Caudal appendage. / 1: Caudal appendage elongate in sexually mature males.

Node 234: RHAMPHICHTHYIDAE clade

ch. 210-Tail length. / 2: Tail long, more than 45% total length.

ch. 218-Mental accessory EO. / 1: present.

Node 235: Steatogenae (*Steatogenys*, *Hypopygus*) clade

ch. 3-Body shape profile. / 0: Body relatively deep in profile, depth at pectoral girdle more than 11% total length.

ch. 4-Snout length short. / 1: Snout short, preorbital length less than one-third total head length.

ch. 8-Oral opening in adults. / 1: Lower jaw extends anterior to upper, oral aperture superior.

ch. 54-Dentary hook. / 1: Ventral margin of anterior portion of dentary bearing a posteriorly oriented process (“dentary hook”), a ventral extension of the medial surface of dentary where it covers the anterior portion of Meckel׳s cartilage.

ch. 55-Dentary teeth size. / 1: Teeth on posterior half of dentary twice the size of anterior teeth.

ch. 71-Ethmoid cartilage. / 1: Ethmoid cartilage deeper than long; antorbital region of snout about as deep as long.

ch. 73-Lateral ethmoid. / 1: Lateral ethmoid not ossified.

ch. 86-Parasphenoid dorsal margin. / 1: Dorsal margin narrow.

ch. 140-Branchiostegal ray morphology. / 0: Anterior 1–2 rays broad.

ch. 143-Anterior pharyngobranchial. / 1: Anterior pharyngobranchial unossified.

ch. 149-Epibranchial 5. / 1: Fifth epibranchial with posterior process.

ch. 153-Hypobranchial 1. / 2: First hypobranchial rounded or pentagonal in dorsal view; anterior margin interrupted by a sharp angle.

ch. 162-Urohyal blade hyperossified. / 1: Urohyal blade long, ossified to level of fourth basibranchial.

ch. 188-Number anal-fin rays. / 1: Anal fin long, extending along majority of ventral body margin; 100–159 rays.

ch. 197-Body cavity short. / 2: Body cavity very short; associated with 11 or fewer precaudal vertebrae.

ch. 208-Urogenital papilla. / 0: Urogenital pore sessile, opening flush with ventral margin of body wall in sexually mature specimens.

ch. 220-Humeral accessory EO. / 1: Humeral electric organ extending dorsally from pectoral fin base, and then posteriorly along horizontal myoseptum a distance less than length of pectoral fin.

Node 238: *Steatogenys* clade

ch. 18-Vertical pigment bars. “Saddle-shaped bars”. / 1–10 dark bars across mid-dorsal surface extending as vertical bands onto lateral surfaces.

ch. 165-Mesocoracoid. / 0: Mesocoracoid ossified within scapulocoracoid cartilage, forming a bridge between medial surface of coracoid and cleithrum.

ch. 166-Anterior coracoid process. / 1: Anterior coracoid process not extending to a vertical with contact of dorsomedial limb of coracoid with cleithrum.

ch. 167-Proximal pectoral radials. / 1: Proximal radials three and four co-ossified in adult specimens.

ch. 219-Mental accessory EO configuration. / 1: Mental accessory EO long, threadlike with many electrocytes.

Node 236: *Hypopygus* clade

ch. 10-Position of anterior nares. / 1: Anterior nares located very close to or within gape, narial opening oriented anteroventrally.

ch. 12-Posterior narial pore. / 1: Posterior narial pore absent.

ch. 17-Vertical pigment lines. / 1: Thin vertical pigment lines present along longitudinal body axis.

ch. 32-Lateral line. / 1: Lateral line incomplete.

ch. 70-Dermal vomer. / 1: Dermal vomer not ossified.

ch. 163-Posttemporal. / 0: Posttemporal independent from supracleithrum in mature specimens.

Node 240: Rhamphichthyinae clade

ch. 5-Snout long. / 1: Snout elongate, frontal, vomer and anterior portion of parasphenoid elongate; preorbital length longer than one-third total head length or greater in mature specimens.

ch. 9-Position of nasal capsule. / 1: Anterior position of nasal capsule; located closer to tip of snout than to eye; posterior nares closer to anterior nares than to anterior margin of eye.

ch. 30-Scales on middorsum. / 1: Scales absent from head, anterior portion of dorsal midline, and area dorsal to pectoral fins.

ch. 33-Lateral line pores. / 1: Posterior lateral line pores tubular; tube length more than three times pore diameter.

ch. 59-Posterior limb anguloarticular. / 1: Posterior limb of anguloarticular large; its ventral margin longer than that of retroarticular.

ch. 61-Mesethmoid length. / 1: Mesethmoid elongate, its length greater than antorbital region of frontal.

ch. 87-Parasphenoid process. / 0: Anteroventral margin of parasphenoid smooth.

ch. 116-Anterior extent of eminentia granularis. / 1: EG small, its anterior margin not extending to contact optic tectum.

ch. 124-Endopterygoid anterior process. / 1: Entire extent of ligament ossified, forming a bony strut anterior to orbit; process equally as wide along most of its length.

ch. 129-Size of symplectic. / 1: Length of symplectic greater than hyomandibula.

ch. 130-Orientation of hyomandibula. / 1: Main axis of hyomandibula oriented horizontally, parallel to main axis of neurocranium.

ch. 138-Opercular dorsal margin. / 1: Dorsal margin of opercle straight.

ch. 178-DHS anterior series. / 1: Anterior series of 8–14 DHSs in hypaxial musculature lateral to body cavity.

ch. 181-DHS 2 shape. / 0: Second posterior DHS straight.

ch. 187-Anal fin origin. / 2: Anal fin origin near branchial isthmus.

ch. 189-Anal-fin rays unbranched. / 2: 30–60 unbranched anal-fin rays.

ch. 190-Anal-fin pterygiophore (AFP) length. / 1: Anal-fin pterygiophores longer than hemal spines at midbody; more than one-third total body depth (less than 1.5 times into depth of axial musculature).

Node 241: *Gymnorhamphichthys* clade

ch. 30-Scales on middorsum. / 2: Scales absent along entire middorsum.

ch. 43-Anterior maxillary process. / 1: Anterior process of maxilla extends anterior to articulation of maxilla and autopalatine, forming a tapered process, its ventral margin continuous with descending blade of maxilla; maxilla forked in lateral view.

ch. 51-Dentary dorsal margin. / 1: Dorsal margin of dentary concave.

ch. 69-Ventral ethmoid-Vomer. / 1: Ventral ethmoid and vomer not fused in adults

ch. 82-Orbitosphenoid margin. “Sphenoid fenestra” / 1: Posterior margin of orbitosphenoid not contacting pterosphenoid, except between dorsal portion of their common margin; presence of an unmineralized fenestra between orbitosphenoid and pterosphenoid.

Node 249: Rhamphichthyini (*Iracema*, *Rhamphichthys*) clade

ch. 17-Vertical pigment lines. / 1: Thin vertical pigment lines present along longitudinal body axis.

ch. 34-Lateral line ventral rami. / 1: Numerous ventral rami extending parallel with lateral line.

ch. 56-M. Adductor mandibula. / 1: Belly of Adductor mandibulae muscle with ossified intermuscular bones, oriented parallel to main axis of muscle fibers.

ch. 122-Endopterygoid ascending process. / 1: Ascending process on lateral surface of endopterygoid; pterygocranial ligament ossified; base of ascending process situated approximately dorsal to articulation of quadrate with anguloarticular.

ch. 156-Basihyal dorsal ridge. / 1: Dorsal surface of basihyal convex along its long axis, forming a ridge.

ch. 165-Mesocoracoid. / 0: Mesocoracoid ossified within scapulocoracoid cartilage, forming a bridge between medial surface of coracoid and cleithrum.

ch. 188-Number anal-fin rays. / 3: 200–299 rays. Taxa coded by modal number of anal-fin rays.

Node 250: *Rhamphichthys* clade

ch. 138-Opercular dorsal margin. / 0: Dorsal margin of opercle convex. 1: Dorsal margin of opercle straight.

ch. 162-Urohyal blade hyperossified. / 1: Urohyal blade long, ossified to level of fourth basibranchial.

ch. 188-Number anal-fin rays. / 4: 300 or more rays. Taxa coded by modal number of anal-fin rays.

ch. 189-Anal-fin rays unbranched. / 0: 10–15 anal-fin rays branched into two rami about half distance to their tips.

ch. 205-Gas bladder. / 1: Anterior chamber of gas bladder encapsulated in a thick, opaque layer of tissue.

Node 259: SINUSOIDEA clade

ch. 43-Anterior maxillary process. / 1: Anterior process of maxilla extends anterior to articulation of maxilla and autopalatine, forming a tapered process, its ventral margin continuous with descending blade of maxilla; maxilla forked in lateral view (Albert, 2001-Figs. 6–7).

ch. 88-Posttemporal fossa. / 1: Epioccipital, pterotic, and parietal bones not contacting one another along their mutual margins, forming a fossa in posttemporal region; lateral surface of otic capsule exposed.

ch. 94-Antorbital. / 1: Infraorbital canal extending onto antorbital.

ch. 117-Posterior EG. / 1: Posterior lobe of EG well developed, wrapped around caudal lobe of cerebellum, its posterior margin extending to a vertical with posterior margin of ELL.

ch. 122-Endopterygoid ascending process. / 1: Ascending process on lateral surface of endopterygoid; pterygocranial ligament ossified; base of ascending process situated approximately dorsal to articulation of quadrate with anguloarticular.

ch. 146-Epibranchial 4. / 1: Fourth epibranchial with short posterior process.

ch. 173-Position of neural spines. / 1: Neural spine inserting on posterior margin of caudal vertebral centra.

ch. 174-Vertebral fenestrae. / 1: Lateral walls of neural arches with several small fenestrae; dorsal margin uneven, with several evaginations.

ch. 179-DHS 1. / 1: Anterior DHS large, two to three times as broad as other hemal spines, often exhibiting additional distal tips. In the derived state the anterior DHS is curved and scythe shaped.

ch. 180-DHS 1. / 1: Proximal surface of first DHS broad as blade.

ch. 188-Number anal-fin rays. / 3: 200–299 rays. Taxa coded by modal number of anal-fin rays.

ch. 189-Anal-fin rays unbranched. / 1: Anterior 15–25 rays anal-fin rays unbranched to their tips.

ch. 194-Distal AFP. / 0: Distal anal-fin pterygiophores present.

ch. 217-Hypaxial EO ontogeny. / 1: Hypaxial electric organ replaced during development, adult organ not derived from hypaxial musculature.

ch. 222-EOD form. / 1: EOD produced as a continual series of discharges to form a quasi-sinusoidal pattern of current emission; no capacity for EOD frequency modulations; relay and pacemaker cells mingled in a single medullary nucleus.

ch. 223-EOD monophasic in adults. / 1: Monophasic EOD of juveniles retained into maturity; EOD characterized exclusively by head-positive depolarizations.

Node 260: STERNOPYGIDAE clade

ch. 8-Oral opening in adults. / 0: Upper and lower jaws of equal length, oral aperture terminal.

ch. 13-Eye size. / 0: Eye and optic tract large; about two eye diameters into postorbital head length.

ch. 37-Tooth shape. / 1: Teeth in both jaws villiform, each tooth a long cylindrical shaft with a narrow base.

ch. 57-M. Adductor mandibulae. / 1: Additional insertion of A1 on first infraorbital.

ch. 72-Lateral ethmoid size. / 0: Lateral ethmoid a large endochondral ossification in the antorbital region, arching laterally over Profundus (V1) nerve, with four margins; anterolateral process contacting ventral ethmoid, posteromedial process contacting parasphenoid, dorsomedial margin contacting frontal, and anteromedial margin contacting mesethmoid.

ch. 75-Nasal. / 1: Nasal broad.

ch. 77-Antorbital process frontals. / 1: Lateroventral process of frontals anterior to orbit.

ch. 97-Infraorbital canal plates. / 1: Antorbital and infraorbitals 1–4 large, partial cylinders with slender osseous arches.

ch. 100-Mandibular canal. / 0: Canal bearing bones of preopercular–mandibular laterosensory canal long and slender ossifications embedded in dermis; diameter of canal slender.

ch. 106-Accessory optic system. / 0: Accessory optic tract large, easily visible in histological sections; neurons of tract organized into a distinct tegmental cell cluster (i.e., accessory optic nucleus).

ch. 133-Mandibular canal size. / 1: Mandibular canal ossicles dumbbell-shaped.

ch. 152-Ceratobranchial 4. “Lateral process of sixth ceratobranchial”. / 1: Lateral surface of fourth ceratobranchial with an anterolaterally oriented process.

ch. 172-Anterior vertebrae. / 1: Parapophyses of second vertebrae separated by distinct gap from the os suspensorium.

ch. 176-Caudal intermusculars. / 1: Capacity to regenerate ossified intermuscular bones.

ch. 197-Body cavity short. / 0: Body cavity associated with 16–19 vertebrae.

Node 261: *Sternopygus* clade

ch. 14-Position of eye. / 0: Surface of eye not covered by epidermis in adults; free orbital margin.

ch. 20-Longitudinal lines. / 2: A white narrow stripe extending parallel to the base of the anal-fin pterygiophores, and then posteriorly along the lateral midline.

ch. 27-Branchial opening. / 0: Branchial opening extends along entire posterior margin of opercle, from isthmus to pectoral fin insertion.

ch. 63-Mesethmoid, tip shape. / 0: Portion of mesethmoid anterior to ventral ethmoid horizontal; its dorsal surface anterior and posterior to ventral ethmoid approximately parallel; its ventral surface parallel with dorsal surface.

ch. 68-Median septum of ventral ethmoid. / 1: Ossified median septum of ventral ethmoid elongate in mature specimens, longer than deep, extending posterior to posterior margin of lateral process.

ch. 125-Mesopterygoid dentition. / 0: Numerous small teeth distributed in an irregular field on anterior portion of ventral surface of endopterygoid.

ch. 163-Posttemporal. / 0: Posttemporal independent from supracleithrum in mature specimens.

ch. 189-Anal-fin rays unbranched. / 3: all anal-fin rays unbranched.

ch. 216-Main EO electrocyte morphology. / 0: Electrocytes cigar shaped, elongate; longitudinal axis parallel with neuraxis.

Node 265: Eigenmanninae clade

ch. 4-Snout length short. / 1: Snout short, preorbital length less than one-third total head length.

ch. 26-Body translucence. / 1: Body translucent in living specimens, yellow or pink hue in living specimens, yellow or hyaline in formalin-fixed specimens, melanophores sparse or absent on lateral body surface.

ch. 83-Sphenotic process. / 1: Dorsolateral margin of sphenotic bearing a transversely oriented crest or process exposed on dorsolateral edge, anterior margin not underlying frontal.

ch. 146-Epibranchial 4. / 0: Posterior margin of fourth epibranchial flat.

ch. 148-Shape of 4th epibranchial. / 0: Fourth epibranchial with short ascending process.

ch. 164-Scapular foramen. / 1: Unossified region of scapulocoracoid cartilage included entirely within the scapula, forming a large foramen.

ch. 167-Proximal pectoral radials. / 1: Proximal radials three and four co-ossified in adult specimens.

ch. 175-Shape anterior intermusculars. / 1: Intermusculars highly branched.

ch. 180-DHS 1 proximal surface. / 0: Proximal surface of first DHS narrower than descending blade.

ch. 188-Number anal-fin rays. / 2: 160–199 rays.

ch. 197-Body cavity short. / 2: Body cavity very short; associated with 11 or fewer precaudal vertebrae.

ch. 199-Number of pleural ribs. / 1: Seven or fewer pairs of pleural ribs.

ch. 200-Length of anterior ribs. / 1: Length of anterior two ribs greater than 80% body depth at pectoral girdle.

Node 266: *Rhabdolichops* clade

ch. 30-Scales on middorsum. / 1: Scales absent from head, anterior portion of dorsal midline, and area dorsal to pectoral fins.

ch. 43-Anterior maxillary process. / 0: Anterior process of maxilla absent.

ch. 46-Maxilla descending blade. / 1: Descending blade of maxilla broad, connective tissue membrane along its anteroventral margin ossified to form a thin shelf; anterior portion of maxilla rhomboid in lateral view.

ch. 86-Parasphenoid dorsal margin. / 1: Dorsal margin narrow.

ch. 87-Parasphenoid process. / 1: Parasphenoid with anteroventral process.

ch. 182-Number posterior DHS. / 1: A single DHS posterior to large anterior spine.

ch. 190-Anal-fin pterygiophore (AFP) length. / 1: Anal-fin pterygiophores longer than hemal spines at midbody; more than one-third total body depth (less than 1.5 times into depth of axial musculature).

Node 270: Eigenmannini (*Distocyclus*, *Archolaemus*, *Japigny*, *Eigenmannia*) clade

ch. 88-Posttemporal fossa. / 0: Epioccipital, pterotic and parietal bones contact one another along their mutual margins, forming a bony surface in posttemporal region of neurocranium; lateral surface of otic capsule enclosed.

Node 271: *Distocyclus*+*Archolaemus* clade

ch. 4-Snout length short. / 0: Preorbital length about one-third total head length in mature specimens.

ch. 63-Mesethmoid, tip shape. / 0: Portion of mesethmoid anterior to ventral ethmoid horizontal; its dorsal surface anterior and posterior to ventral ethmoid approximately parallel; its ventral surface parallel with dorsal surface.

ch. 147-Epibranchial 3. / 1: Third epibranchial sinuous.

ch. 148-Shape of 4th epibranchial. / 1: Fourth epibranchial with an elongate ascending process.

ch. 180-DHS 1. / 1: Proximal surface of first DHS broad as blade.

ch. 188-Number anal-fin rays. / 3: 200–299 rays. Taxa coded by modal number of anal-fin rays.

Node 272: *Eigenmannia* clade

No known diagnostic character.

Node 273: *Japigny*+*Eigenmannia macrops* clade

ch. 8-Oral opening in adults. / 2: Upper jaw extends anterior to lower, oral aperture inferior.

Node 279: APTERONOTIDAE clade

ch. 90-Cranial skeleton texture. / 1: Surface of many cranial bones pitted and/or reticular in appearance, excavated during ontogeny to form small pits and vesicles.

ch. 91-Nasal loop. / 1: Commissure between infraorbital and supraorbital canals extended anteriorly, forming a loop ventrolateral to nasal capsule; antorbital and first infraorbital bones situated near anterior nares.

ch. 92-Infraorbital subnasal extension. / 1: Anterior extension of infraorbital canal shorter than width of canal pore; anterior canal pore of infraorbital canal situated near first infraorbital.

ch. 119-Pacemaker nucleus. / 1: Pacemaker nucleus large, visible as a median, ovoid eminence on ventral surface of medulla; its ventral margin extending to medullary surface.

ch. 131-Hyomandibular articulation. / 1: Proximal portion of hyomandibula narrow; articulating surface facing dorsally.

ch. 136-Anterior limb preoperculum. / 1: Preopercle narrow, curved; ventral margin of anterior limb not ossified.

ch. 138-Opercular dorsal margin. / 1: Dorsal margin of opercle straight.

ch. 139-Branchiostegal rays. / 0: 3–4 rays.

ch. 143-Anterior pharyngobranchial. / 1: Anterior pharyngobranchial unossified.

ch. 144-Pharyngobranchials. / 1: Pharyngobranchials of third and fourth arches ossified.

ch. 149-Epibranchial 5. / 1: Fifth epibranchial with posterior process.

ch. 151-Ceratobranchial 2. / 1: Posterior surface of second ceratobranchial with a medially oriented process.

ch. 156-Basihyal dorsal ridge. / 1: Dorsal surface of basihyal convex along its long axis, forming a ridge.

ch. 182-Number posterior DHS. / 1: A single DHS posterior to large anterior spine.

ch. 183-Dorsal organ. / 1: Posterodorsal margin of body with a median flap or bar of fleshy tissue, extending parallel to the dorsal margin of epaxial musculature.

ch. 190-Anal-fin pterygiophore (AFP) length. / 1: Anal-fin pterygiophores longer than hemal spines at midbody; more than one-third total body depth (less than 1.5 times into depth of axial musculature).

ch. 202-Posterior parapophyses. / 1: Parapophyses of posterior precaudal vertebra longer than wide, their ventral margins parallel with long axis of body, abutting at midline.

ch. 203-Shape last precaudal parapophyses. / 1: Parapophyses of last precaudal vertebra slender and sinuous, their tips pointed.

ch. 210-Tail length. / 1: Tail short, 0–16% total length.

ch. 211-Elongate caudal rod. / 0: Caudal fin present with hypural plate and segmented rays.

ch. 213-Caudal fin. / 0: Caudal fin present.

ch. 221-Neural EO. / 1: Main electric organ of mature specimens derived from electromotor neurons which innervate larval hypaxial organ.

Node 280: Sternarchorhamphinae (*Orthosternarchus*, *Sternarchorhamphus*) clade

ch. 5-Snout long. / 1: Snout elongate, frontal, vomer and anterior portion of parasphenoid elongate; preorbital length longer than one-third total head length or greater in mature specimens.

ch. 9-Position of nasal capsule. / 1: Anterior position of nasal capsule; located closer to tip of snout than to eye; posterior nares closer to anterior nares than to anterior margin of eye.

ch. 30-Scales on middorsum. / 1: Scales absent from head, anterior portion of dorsal midline, and area dorsal to pectoral fins.

ch. 45-Anterior maxillary shelf. / 1: Anterior process of maxilla large and broad, extending more than one half the length of the descending blade in mature specimens.

ch. 49-Rows of dentary teeth. / 1: Teeth on dentary arranged in two to three rows at its midlength.

ch. 61-Mesethmoid length. / 1: Mesethmoid elongate, its length greater than antorbital region of frontal.

ch. 63-Mesethmoid, tip shape. / 0: Portion of mesethmoid anterior to ventral ethmoid horizontal; its dorsal surface anterior and posterior to ventral ethmoid approximately parallel; its ventral surface parallel with dorsal surface.

ch. 68-Median septum of ventral ethmoid. / 1: Ossified median septum of ventral ethmoid elongate in mature specimens, longer than deep, extending posterior to posterior margin of lateral process.

ch. 74-Base lateral ethmoid. / 1: Lateral ethmoid narrow or tubular; length of its base less than one-third length of its anterior margin.

ch. 78-Dorsal margin of frontals. / 1: Portion of frontal anterior to orbit concave in lateral profile

ch. 105-Eyeball extrinsic muscles. / 1: Extrinsic eyeball muscles and innervating nerves small or absent, their diameters about the same as collagen fibers.

ch. 122-Endopterygoid ascending process. / 0: Lateral surface of endopterygoid smooth; no ascending process ossified in pterygocranial ligament (connecting endopterygoid with neurocranium).

ch. 123-Endopterygoid ascending process. / 1: Small ascending process of endopterygoid in juveniles obliterated by growth along dorsal margin of bone; no endopterygoid process in adults.

ch. 128-Metapterygoid posterior wing. / 1: Metapterygoid elongate and narrow, longer than wide at its midlength.

ch. 165-Mesocoracoid. / 0: Mesocoracoid ossified within scapulocoracoid cartilage, forming a bridge between medial surface of coracoid and cleithrum.

ch. 184-Dorsal organ length. / 1: Dorsal organ extends along entire dorsal margin of body, from nape to caudal peduncle.

ch. 187-Anal fin origin. / 2: Anal fin origin near branchial isthmus.

ch. 189-Anal-fin rays unbranched. / 3: all anal-fin rays unbranched.

ch. 191-Shape of AFP blades. / 1: Descending blades of anal-fin pterygiophores broad, anterior and posterior margins extending into ventral median septum in cross section.

ch. 192-Shape of AFP tips. / 1: Tips of pterygiophores shaped like an arrow-head; axial series of pterygiophores providing the ventral margin of the anal-fin base a scalloped appearance.

Node 281: Apteronotinae clade

ch. 67-Ventral ethmoid lateral process shape. / 1: Lateral process of ventral ethmoid robust, posterior surface forming articulation with lateral ethmoid cartilage broad and rounded, covered by a cartilage cap.

ch. 153-Hypobranchial 1. / 1: First hypobranchial triangular in dorsal view. 2: First hypobranchial rounded or pentagonal in dorsal view; anterior margin interrupted by a sharp angle.

ch. 188-Number anal-fin rays. / 1: Anal fin long, extending along majority of ventral body margin; 100–159 rays.

ch. 201-Size of anterior ribs. / 1: Anterior ribs broad, breadth two to three times width.

ch. 223-EOD monophasic in adults. / 0: EOD of mature specimens with two (sometimes three or four) phases; EOD characterized by both head-positive and head-negative depolarizations.

Node 282: *Adontosternarchus* clade

ch. 4-Snout length short. / 1: Snout short, preorbital length less than one-third total head length.

ch. 8-Oral opening in adults. / 0: Upper and lower jaws of equal length, oral aperture terminal.

ch. 36-Adult dentition. / 1: Oral teeth present in juveniles, lost and not replaced during development.

ch. 40-Snout length short. / 1: Snout short, preorbital length less than one-third total head length.

ch. 47-Maxilla descending blade. / 2: Anteroventral margin of descending blade not ossified; distal half of blade extending as a narrow process with a sharp point at its distal tip.

ch. 50-Dentary gracile. / 1: Dentary gracile, posterodorsal process tapering to a point (except in Adontosternarchus sachsi), ventral margin concave.

ch. 71-Ethmoid cartilage. / 1: Ethmoid cartilage deeper than long; antorbital region of snout about as deep as long.

ch. 81-Orbitosphenoid shape. / 1: Anterior margin of orbitosphenoid not ossified, orbitosphenoid narrow, its ventral margin about as long or shorter than its dorsal margin.

ch. 85-Parasphenoid ventral margin. / 1: Ventral margin of parasphenoid flexed sharply on either side of the basicranial region; ventral margin of sphenoid region oblique relative to long axis of neurocranium.

ch. 115-Lateral line afferents. / 0: Lateral line afferents from electrosensory periphery intermingled as they course into the electrosensory lateral line lobe (ELL); fibers from different lateral line nerves not segregated.

ch. 138-Opercular dorsal margin. / 0: Dorsal margin of opercle convex. 1: Dorsal margin of opercle straight.

ch. 139-Branchiostegal rays. / 1: 5–6 rays.

ch. 161-Urohyal blade. / 1: Posterior blade of urohyal unossified, anterior head of urohyal positioned ventral to second basibranchial.

ch. 202-Posterior parapophyses. / 0: Parapophyses of posterior precaudal vertebra small, their ventral margins oblique to long axis of body, not contacting one another along midline.

Node 286: Apteronotini+Sternarchorhynchini+Navajini clade

ch. 64-Mesethmoid, tip size. / 1: Portion of mesethmoid anterior to ventral ethmoid flexed ventrally in mature specimens; its dorsal surface anterior and posterior to ventral ethmoid at an oblique angle; its ventral surface oblique to dorsal surface. terminal.

ch. 92-Infraorbital subnasal extension. / 0: Anterior portion of infraorbital canal extending anterior from first infraorbital ventral to nasal capsule; anterior canal pore of infraorbital canal situated anterior to first infraorbital.

ch. 203-Shape last precaudal parapophyses. / 0: Parapophyses of last precaudal vertebra broad and triangular, their tips rounded.

Node 287: Apteronotini (*Parapteronotus*, *Megadontognathus*, *Apteronotus*) clade

ch. 6-Gape large. / 1: Rictus extends posterior to a vertical through eye, gape forming more than one-third total head length.

ch. 7-Gape short. / 0: Rictus extends ventral to nasal capsule, gape more than three times eye diameter, oriented parallel with long axis of head.

ch. 46-Maxilla descending blade. / 1: Descending blade of maxilla broad, connective tissue membrane along its anteroventral margin ossified to form a thin shelf; anterior portion of maxilla rhomboid in lateral view.

ch. 190-Anal-fin pterygiophore (AFP) length. / 0: Anal-fin pterygiophores shorter than hemal spines at midbody; less than one-third total body depth (more than 1.5 times into depth of axial musculature).

Node 289: *Megadontognathus*+*Apteronotus* clade

ch. 21-Pigment contrast. / 1: High contrast dark brown or black and white pigments on body surface.

ch. 58-Anterior limb anguloarticular. / 1: Anterior limb of anguloarticular shorter than posterior limb.

ch. 90-Cranial skeleton texture. / 0: Surface of endochondral and dermal ossifications of cranial skeleton composed of lamellar or cancellous bone.

ch. 197-Body cavity short. / 0: Body cavity associated with 16–19 vertebrae.

ch. 210-Tail length. / 0: Length of tail posterior to anal-fin 17–45% total length.

Node 290: *Apteronotus* clade

ch. 19-Caudal Peduncle Spot. / 1: Pale spot present at base of caudal region. Newly coded herein.

ch. 23-White mid-sagittal pigments. / 1: Mid-sagittal region of dorsal and mental surfaces bright white.

ch. 154-Hypobranchial 2. / 1: Anterior tip of second hypobranchial with a large medially oriented process, contacting contralateral third hypobranchial across midline by means of a cartilaginous bridge.

Node 291: *Apteronotus magdalenensis*+*A. leptorhynchus* clade

ch. 5-Snout long. / 1: Snout elongate, frontal, vomer and anterior portion of parasphenoid elongate; preorbital length longer than one-third total head length or greater in mature specimens.

ch. 80-Sphenoid region. / 1: Sphenoid region of neurocranium more than one-third total head length, combined axial length of the orbitosphenoid and pterosphenoid bones greater than preorbital region.

Node 292: *Apteronotus magdalenensis* clade

ch. 6-Gape large. / 0: Rictus of mouth extends ventral to nasal capsule, gape forming less than one-third total head length.

ch. 25-Pigment distribution. / 1: Black and white pigments distributed unevenly over body surface, darker and paler areas grading into one another; integument with a marbled or mottled appearance.

ch. 122-Endopterygoid ascending process. / 0: Lateral surface of endopterygoid smooth; no ascending process ossified in pterygocranial ligament (connecting endopterygoid with neurocranium).

ch. 123-Endopterygoid ascending process. / 1: Small ascending process of endopterygoid in juveniles obliterated by growth along dorsal margin of bone; no endopterygoid process in adults.

Node 293: *Apteronotus leptorhynchus* clade

ch. 55-Dentary teeth size. / 1: Teeth on posterior half of dentary twice the size of anterior teeth.

Node 294: *Apteronotus albifrons* clade

ch. 22-White posterior bars. / 1: White or pale bars present on caudal region as observed in members of the Apteronotus albifrons species group. Newly coded herein.

ch. 197-Body cavity short. / 1: Body cavity short; associated with 12–15 precaudal vertebrae.

Node 298: Sternarchorhynchini+Navajini clade

No known diagnostic character.

Node 299: Sternarchorhynchini (*Platyurosternarchus*, *Sternarchorhynchus*) clade

ch. 5-Snout long. / 1: Snout elongate, frontal, vomer and anterior portion of parasphenoid elongate; preorbital length longer than one-third total head length or greater in mature specimens.

ch. 40-Snout length short. / 1: Snout short, preorbital length less than one-third total head length.

ch. 43-Anterior maxillary process. / 0: Anterior process of maxilla absent.

ch. 59-Posterior limb anguloarticular. / 1: Posterior limb of anguloarticular large; its ventral margin longer than that of retroarticular.

ch. 61-Mesethmoid length. / 1: Mesethmoid elongate, its length greater than antorbital region of frontal.

ch. 64-Mesethmoid tip groove. / 0: Anterior surface of mesethmoid flat or convex.

ch. 67-Ventral ethmoid lateral process shape. / 0: Lateral process of ventral ethmoid narrow, flattened horizontally, posterior surface articulating with lateral ethmoid cartilage.

ch. 68-Median septum of ventral ethmoid. / 0: Portion of ventral ethmoid ossified within medial nasal septum approximately as long as deep; posterior margins of median septum and lateral process of ventral ethmoid approximately equal.

ch. 73-Lateral ethmoid. / 1: Lateral ethmoid not ossified.

ch. 84-Parasphenoid lateral process. / 0: Lateral margins of parasphenoid extending as broad dorsolateral processes anterior to prootic, extending to a horizontal with trigeminal foramen.

ch. 122-Endopterygoid ascending process. / 0: Lateral surface of endopterygoid smooth; no ascending process ossified in pterygocranial ligament (connecting endopterygoid with neurocranium).

ch. 123-Endopterygoid ascending process. / 1: Small ascending process of endopterygoid in juveniles obliterated by growth along dorsal margin of bone; no endopterygoid process in adults.

ch. 128-Metapterygoid posterior wing. / 1: Metapterygoid elongate and narrow, longer than wide at its midlength.

ch. 153-Hypobranchial 1. / 0: First hypobranchial rectangular in dorsal view; anterior margin straight.

ch. 187-Anal fin origin. / 2: Anal fin origin near branchial isthmus.

ch. 188-Number anal-fin rays. / 2: 160–199 rays.

ch. 191-Shape of AFP blades. / 1: Descending blades of anal-fin pterygiophores broad, anterior and posterior margins extending into ventral median septum in cross section.

ch. 202-Posterior parapophyses. / 0: Parapophyses of posterior precaudal vertebra small, their ventral margins oblique to long axis of body, not contacting one another along midline.

Node 300: *Platyurosternarchus* clade

ch. 6-Gape large. / 1: Rictus extends posterior to a vertical through eye, gape forming more than one-third total head length.

ch. 7-Gape short. / 0: Rictus extends ventral to nasal capsule, gape more than three times eye diameter, oriented parallel with long axis of head.

ch. 125-Mesopterygoid dentition. / 0: Numerous small teeth distributed in an irregular field on anterior portion of ventral surface of endopterygoid.

ch. 182-Number posterior DHS. / 0: Two or three DHSs posterior to large anterior spine.

ch. 188-Number anal-fin rays. / 3: 200–299 rays. Taxa coded by modal number of anal-fin rays.

Node 301: *Sternarchorhynchus* clade

ch. 8-Oral opening in adults. / 0: Upper and lower jaws of equal length, oral aperture terminal.

ch. 9-Position of nasal capsule. / 1: Anterior position of nasal capsule; located closer to tip of snout than to eye; posterior nares closer to anterior nares than to anterior margin of eye.

ch. 23-White mid-sagittal pigments. / 1: Mid-sagittal region of dorsal and mental surfaces bright white.

ch. 45-Anterior maxillary shelf. / 0: Anterior process of maxilla extending as a shelf of bone less than one-third the length of the descending blade.

ch. 53-Dentary filamentous. / 1: Dentary elongate and filamentous, more than four times as long as deep.

ch. 81-Orbitosphenoid shape. / 1: Anterior margin of orbitosphenoid not ossified, orbitosphenoid narrow, its ventral margin about as long or shorter than its dorsal margin.

ch. 94-Antorbital. / 0: Infraorbital canal not extending onto antorbital.

ch. 139-Branchiostegal rays. / 1: 5–6 rays.

ch. 146-Epibranchial 4. / 0: Posterior margin of fourth epibranchial flat.

Node 312: Navajini clade

ch. 26-Body translucence. / 1: Body translucent in living specimens, yellow or pink hue in living specimens, yellow or hyaline in formalin-fixed specimens, melanophores sparse or absent on lateral body surface.

ch. 30-Scales on middorsum. / 2: Scales absent along entire middorsum.

ch. 31-Scale shape. / 1: Scales dorsal to lateral line rhomboid, their long axis oriented oblique to long axis of body, their dorsoventral axes longer than their longitudinal axes.

ch. 49-Rows of dentary teeth. / 1: Teeth on dentary arranged in two to three rows at its midlength.

ch. 101-Mandibular canal ossicles. / 1: Canal bearing bones of mandibular laterosensory canal ossified as short, broad, dumbbell-shaped ossicles.

Node 313: Sternarchellini (*Pariosternarchus*, *Sternarchella*, *Magosternarchus*) clade

ch. 51-Dentary dorsal margin. / 1: Dorsal margin of dentary concave.

ch. 89-Supraoccipital crest. / 1: dorsal margin of supraoccipital crest exceed dorsal margin of parietals.

ch. 92-Infraorbital subnasal extension. / 1: Anterior extension of infraorbital canal shorter than width of canal pore; anterior canal pore of infraorbital canal situated near first infraorbital.

ch. 124-Endopterygoid anterior process. / 1: Entire extent of ligament ossified, forming a bony strut anterior to orbit; process equally as wide along most of its length.

ch. 141-Gill raker configuration. / 0: Gill rakers directly attached to gill arches.

ch. 142-Gill raker tips. / 1: Distal tips of gill rakers cartilaginous.

ch. 150-Epibranchial 5 post-med. process. / 1: Posterior surface of seventh epibranchial with a dorsoventrally oriented process.

Node 314: *Sternarchella terminalis*+*S. calhamazon*+*Magosternarchus* clade

ch. 4-Snout length short. / 1: Snout short, preorbital length less than one-third total head length.

ch. 8-Oral opening in adults. / 0: Upper and lower jaws of equal length, oral aperture terminal.

ch. 39-Premaxilla size. / 0: Large. Lateral margin of premaxilla longer than lateral margin of maxilla, premaxilla extends posterodorsal to articulation of maxilla with autopalatine; articular surface of maxilla with autopalatine oriented anterodorsally.

ch. 43-Anterior maxillary process. / 0: Anterior process of maxilla absent.

ch. 44-Maxillary articulation with palatine. / 1: Articular surface of maxilla on a stalk, articulation with autopalatine at end of a bony process; ethmopalatine cartilage a small block attached firmly to articular head of maxilla.

ch. 45-Anterior maxillary shelf. / 0: Anterior process of maxilla extending as a shelf of bone less than one-third the length of the descending blade.

ch. 63-Mesethmoid, tip shape. / 0: Portion of mesethmoid anterior to ventral ethmoid horizontal; its dorsal surface anterior and posterior to ventral ethmoid approximately parallel; its ventral surface parallel with dorsal surface.

ch. 65-Ventral ethmoid size. / 1: Ventral ethmoid robust.

ch. 67-Ventral ethmoid lateral process shape. / 2: Lateral process of ventral ethmoid large and fan-shaped.

ch. 72-Lateral ethmoid size. / 0: Lateral ethmoid a large endochondral ossification in the antorbital region, arching laterally over Profundus (V1) nerve, with four margins; anterolateral process contacting ventral ethmoid, posteromedial process contacting parasphenoid, dorsomedial margin contacting frontal, and anteromedial margin contacting mesethmoid.

ch. 101-Mandibular canal ossicles. / 0: Canal bearing bones of mandibular laterosensory canal long and slender tubes.

ch. 155-Hypobranchial teeth. / 1: Seven or fewer teeth present on sixth hypobranchial.

Node 316: clade comprised of *Sternarchogiton*, *Compsaraia*, *Porotergus*, *"Apteronotus" bonapartii*

ch. 19-Caudal Peduncle Spot. / 1: Pale spot present at base of caudal region. Newly coded herein.

ch. 46-Maxilla descending blade. / 1: Descending blade of maxilla broad, connective tissue membrane along its anteroventral margin ossified to form a thin shelf; anterior portion of maxilla rhomboid in lateral view.

ch. 161-Urohyal blade. / 1: Posterior blade of urohyal unossified, anterior head of urohyal positioned ventral to second basibranchial.

ch. 168-Pectoral fin. / 1: Pectoral fin, less than 43% head length.

ch. 210-Tail length. / 0: Length of tail posterior to anal-fin 17–45% total length.

Node 317: *Sternarchogiton* clade

ch. 4-Snout length short. / 1: Snout short, preorbital length less than one-third total head length (Albert, 2001-Fig. 13).

ch. 40-Snout length short. / 1: Snout short, preorbital length less than one-third total head length (Albert, 2001-Fig. 13).

ch. 47-Maxilla descending blade. / 1: Ventral margin of descending blade with a sharp angle about two-thirds distance to its tip; ventral margin posterior to this angle relatively straight.

ch. 49-Rows of dentary teeth. / 0: A single row of teeth on dentary.

ch. 50-Dentary gracile. / 1: Dentary gracile, posterodorsal process tapering to a point (except in Adontosternarchus sachsi), ventral margin concave.

ch. 74-Base lateral ethmoid. / 1: Lateral ethmoid narrow or tubular; length of its base less than one-third length of its anterior margin.

ch. 81-Orbitosphenoid shape. / 1: Anterior margin of orbitosphenoid not ossified, orbitosphenoid narrow, its ventral margin about as long or shorter than its dorsal margin.

ch. 85-Parasphenoid ventral margin. / 1: Ventral margin of parasphenoid flexed sharply on either side of the basicranial region; ventral margin of sphenoid region oblique relative to long axis of neurocranium.

ch. 93-Infraorbital–supraorbital prenasal commissure. / 1: Infraorbital–supraorbital prenasal commissure present.

ch. 163-Posttemporal. / 0: Posttemporal independent from supracleithrum in mature specimens.

ch. 191-Shape of AFP blades. / 1: Descending blades of anal-fin pterygiophores broad, anterior and posterior margins extending into ventral median septum in cross section.

ch. 197-Body cavity short. / 2: Body cavity very short; associated with 11 or fewer precaudal vertebrae.

Node 322: *Compsaraia*+*Porotergus gimbeli*+*"Apteronotus" bonapartii* species group clade

ch. 6-Gape large. / 1: Rictus extends posterior to a vertical through eye, gape forming more than one-third total head length.

ch. 7-Gape short. / 0: Rictus extends ventral to nasal capsule, gape more than three times eye diameter, oriented parallel with long axis of head.

ch. 9-Position of nasal capsule. / 1: Anterior position of nasal capsule; located closer to tip of snout than to eye; posterior nares closer to anterior nares than to anterior margin of eye.

ch. 154-Hypobranchial 2. / 1: Anterior tip of second hypobranchial with a large medially oriented process, contacting contralateral third hypobranchial across midline by means of a cartilaginous bridge.

ch. 159-Basibranchial one. / 1: First basibranchial foreshortened and broad, hourglass shaped, breadth at midlength narrower than at either end.

Node 323: *Compsaraia* clade

ch. 5-Snout long. / 1: Snout elongate, frontal, vomer and anterior portion of parasphenoid elongate; preorbital length longer than one-third total head length or greater in mature specimens.

ch. 24-Antorbital stripe. / 1: Melanophores absent from narrow band passing lateral to nares.

ch. 78-Dorsal margin of frontals. / 1: Portion of frontal anterior to orbit concave in lateral profile.

ch. 102-Supratemporal lateralis canal. / 1: Supratemporal laterosensory canal curved at a sharp angle on surface of parietal, extending posterior onto epaxial surface of body; terminal canal pore oriented posteriorly; epidermis overlying supratemporal canal depigmented, forming a pale inverted L shaped patch.

ch. 133-Mandibular canal size. / 1: Mandibular canal ossicles dumbbell-shaped.

Node 324: *Porotergus*+“*Apteronotus” bonapartii* clade

ch. 161-Urohyal blade. 0: Posterior blade of urohyal ossified, extending posterior to fourth basibranchial.

Node 325: “*Apteronotus” bonapartii* clade

ch. 30-Scales on middorsum. / 1: Scales absent from head, anterior portion of dorsal midline, and area dorsal to pectoral fins.

ch. 47-Maxilla descending blade. / 1: Ventral margin of descending blade with a sharp angle about two-thirds distance to its tip; ventral margin posterior to this angle relatively straight.

ch. 84-Parasphenoid lateral process. / 0: Lateral margins of parasphenoid extending as broad dorsolateral processes anterior to prootic, extending to a horizontal with trigeminal foramen.

ch. 168-Pectoral fin. / 0: Pectoral fin large, more than 43% head length.

## Figures and Tables

**Table 1:** Molecular vouchers and respective GenBank accession numbers.

**Species**	Voucher	16S	CYTB	COI	RAG1	RAG2	ZIC1
*Carassius auratus 040213*	JSA 040213	KR260091	KR491681	KR491521		KR491745	
*Cyphocharax festivus 33743*	MUSM 33743	KR260092	KR491715	KR491522		KR491746	
*Erythrinus erythrinus 33720*	MUSM 33720	KR260093	KR491714	KR491518			
*Serrasalmus rhombeus 33812*	MUSM 33812	KR260094	KR491716	KR491523			
*Charax tectifer 33862*	MUSM 33862	KR260095	KR491628	KR491519			
*Dianema longibarbis 39374*	MUSM 39374	KR260096	KR491626	KR491520			
*Pseudostegophilus nemurus 33774*	MUSM 33774	KR260098	KR491682	KR491524			
*Pterygoplichthys multiradiatus 39367*	MUSM 39367	KR260099	KR491678	KR491517		KR491748	
*Brachyplatystoma juruense 39376*	MUSM 39376	KR260097	KR491713	KR491564		KR491747	KR491846
*Electrophorus electricus 39371*	MUSM 39371	KR260100	KR491627	KR491624	KR491738	KR491749	KR491847
*Gymnotus pantherinus 11144*	LBP 11144	KR260101	KR491698	KR491598		KR491750	KR491848
*Gymnotus pantherinus 24536*	LBP 24536	KR260102	KR491699	KR491599		KR491751	KR491849
*Gymnotus pantherinus 31531*	MCP 31531	KR260103	KR491700	KR491600		KR491752	KR491850
*Gymnotus jonasi 34047*	LBP 34047	KR260104	KR491696	KR491597		KR491753	KR491851
*Gymnotus jonasi GQ*	GQ-2016	GQ862671	GQ862619			GQ862567	
*Gymnotus stenoleucus GQ*	GQ-2060	GQ862680	GQ862628			GQ862576	
*Gymnotus coropinae 43746*	LBP 43746	KR260105	KR491694	KR491595		KR491754	
*Gymnotus coropinae 7161*	LBP 7161	KR260106	KR491695	KR491596		KR491755	
*Gymnotus coatesi GQ*	GQ-2042	GQ862657	GQ862605			GQ862553	
*Gymnotus javari GQ*	GQ-2020	GQ862670	GQ862618			GQ862566	
*Gymnotus pedanopterus GQ*	GQ-2058	GQ862678	GQ862626			GQ862574	
*Gymnotus cf anguillaris GQ*	GQ-2091	GQ862646	GQ862594			GQ862542	
*Gymnotus cataniapo GQ*	GQ-2062	GQ862655	GQ862603			GQ862551	
*Gymnotus maculosus*	Brochu-8126						
*Gymnotus panamensis*	Brochu-8021						
*Gymnotus henni*	Brochu-8231						
*Gymnotus ardilai*	Brochu 8175						
*Gymnotus choco*	Brochu 8209						
*Gymnotus bahianus*	Brochu-7245						
*Gymnotus cylindricus 1201*	LSUMZ 1201	KR260107	KR491701	KR491601	KR491739	KR491756	KR491852
*Gymnotus cylindricus GQ*	GQ-2092	GQ862667	GQ862615			GQ862563	
*Gymnotus tigre 060406*	JSA 060406	KR260108	KR491697	KR491625		KR491757	KR491853
*Gymnotus curupira GQ*	GQ-2009	GQ862665	GQ862613			GQ862561	
*Gymnotus obscurus GQ*	GQ-2017	GQ862675	GQ862623			GQ862571	
*Gymnotus pantanal 31928*	LBP 31928	KR260109	KR491711	KR491603		KR491758	KR491854
*Gymnotus pantanal 32017*	LBP 32017	KR260110	KR491712	KR491604		KR491759	KR491855
*Gymnotus chaviro 39364*	MUSM 39364	KR260111	KR491705	KR491608		KR491760	KR491856
*Gymnotus varzea GQ*	GQ -2014	GQ862687	GQ862635			GQ862583	
*Gymnotus omarorum*	Brochu-7093						
*Gymnotus mamiraua GQ*	GQ-2012	GQ862673	GQ862621			GQ862569	
*Gymnotus sp. “ITAP” or “RS2” 25550*	LBP 25550	KR260112	KR491709	KR491610		KR491761	KR491857
*Gymnotus sylvius 36021*	LBP 36021	KR260113	KR491710	KR491602		KR491762	KR491858
*Gymnotus sp. “IGUA” or “RS1” 14044*	LBP 14044	KR260114	KR491702	KR491611		KR491763	
*Gymnotus sp. “ITAP” or “RS2” 37726*	LBP 37726	KR260115	KR491703	KR491612		KR491764	KR491860
*Gymnotus carapo 36951*	MUSM 36951	KR260116	KR491706	KR491609		KR491765	KR491861
*Gymnotus carapo 27325*	LBP 27325	KR260117	KR491707	KR491606		KR491766	KR491862
*Gymnotus carapo 32294*	LBP 32294	KR260118	KR491704	KR491607		KR491767	KR491863
*Gymnotus carapo 35859*	MUSM 35859	KR260119	KR491708	KR491605		KR491768	KR491864
*Gymnotus ucamara GQ*	GQ-1927	GQ862685	GQ862633			GQ862581	
*Gymnotus arapaima GQ*	GQ-2002	GQ862647	GQ862595			GQ862543	
*Akawaio penak GQ*	GQ-8796		KF533289	KF533336		KF533309	
*Hypopomus artedi GQ*	GQ-2232	GQ862689	GQ862637	KF533331		GQ862585	
*Microsternarchus bilineatus 34063*	LBP 34063	KR260120	KR491692	KR491622		KR491769	KR491865
*Microsternarchus bilineatus 50417*	LBP 50417	KR260121	KR491693	KR491623		KR491770	KR491866
*Racenisia fimbriipinna GQ*	GQ-2339		KF533292	KF533337		KF533311	
*Brachyhypopomus brevirostris GQ*	GQ-7019		KF533280	KF533325		KF533301	
*Brachyhypopomus sp. “PAL” GQ*	GQ-2432	GQ862643	GQ862591			GQ862539	
*Brachyhypopomus sp. “PAL” GQ*	GQ-8783		KF533284	KF533329		KF533305	
*Brachyhypopomus diazi GQ*	GQ-2408	GQ862642	GQ862590			GQ862538	
*Brachyhypopomus occidentalis 1849*	LSUMZ 1849	KR260122	KR491720	KR491565	KR491740	KR491771	KR491867
*Brachyhypopomus_*sp. "roy"*	Sullivan						
Brachyhypopomus bullocki*	Sullivan						
*Brachyhypopomus pinnicaudatus GQ*	GQ-2122		KF533283	KF533328		KF533304	
*Brachyhypopomus brevirostris 16705*	LBP 16705	KR260123	KR491719	KR491613		KR491772	KR491868
*Brachyhypopomus draco 16267*	UFRGS 16267	KR260124	KR491717	KR491614			KR491869
*Brachyhypopomus beebei 39375*	MUSM 39375	KR260125	KR491718	KR491615			KR491870
*Hypopygus lepturus 43739*	LBP 43739	KR260126	KR491679	KR491575		KR491773	KR491871
*Hypopygus neblinae 14841*	UFRGS 14841	KR260127	KR491680	KR491576			KR491872
*Steatogenys duidae 34068*	LBP 34068	KR260128	KR491689	KR491579		KR491774	KR491873
*Steatogenys elegans 182571*	ANSP T3958	KR260129	KR491690	KR491577		KR491775	KR491874
*Steatogenys elegans 19728*	LBP 19728	KR260130	KR491691	KR491578	KR491741	KR491776	KR491875
*Gymnorhamphichthys britskii 22012*	LBP 22012	KR260131	KR491629	KR491566		KR491777	KR491876
*Gymnorhamphichthys britskii 45898*	LBP 45898	KR260132	KR491630	KR491567		KR491778	KR491877
*Gymnorhamphichthys rosamariae 191142*	ANSP T 3926	KR260133	KR491635	KR491573		KR491779	
*Gymnorhamphichthys hypostomus 18063*	ANSP T 1185	KR260134	KR491634	KR491574		KR491780	KR491878
*Gymnorhamphichthys rondoni 191143*	ANSP 191143	KR260135	KR491632	KR491571		KR491781	KR491879
*Gymnorhamphichthys rondoni T09059*	ANSP 09059	KR260136	KR491633	KR491572		KR491782	
*Gymnorhamphichthys rondoni 179673*	ANSP T09059	KR260137	KR491631	KR491570		KR491783	KR491880
*Gymnorhamphichthys rondoni 179685*	ANSP 179673	KR260138	KR491735	KR491569		KR491784	KR491881
*Gymnorhamphichthys rondoni 11515*	LBP 11515	KR260139	KR491736	KR491568	KR491742	KR491785	KR491882
*Rhamphichthys drepanium*	Sullivan						
*Rhamphichthys rostratus GQ*	GQ-2632	GQ862690	GQ862638			GQ862586	
*Rhamphichthys rostratus 187120*	ANSP 187120	KR260140	KR491687	KR491621		KR491786	KR491883
*Rhamphichthys rostratus T3954*	ANSP T3954	KR260141	KR491688			KR491787	
*Rhamphichthys rostratus GQ*	GQ-8825		KF533295	KF533341		KF533317	
*Rhamphichthys apurensis 43111*	LBP 43111	KR260142	KR491684	KR491618		KR491788	KR491884
*Rhamphichthys apurensis T9915*	ANSP T09915	KR260143	KR491685	KR491619		KR491789	KR491885
*Rhamphichthys hahni 19226*	LBP 19226	KR260144	KR491683	KR491616		KR491790	KR491886
*Rhamphichthys lineatus 116566*	UF 116566	KR260145	KR491677	KR491617			KR491887
*Rhamphichthys marmoratus 42545*	LBP 42545	KR260146	KR491686	KR491620	KR491743	KR491792	KR491888
*Distocyclus conirostris 182573*	ANSP 182573	KR260147	KR491726	KR491586		KR491793	
*Archolaemus blax GQ*	GQ-77845	AF072163	GQ228029				
*Eigenmannia virescens 41404*	LBP 41404	KR260148	KR491733	KR491593		KR491794	KR491889
*Eigenmannia macrops 37145*	MUSM 37145	KR260149	KR491728	KR491588		KR491795	KR491890
*Eigenmannia macrops 44284*	LBP 44284	KR260150	KR491729	KR491589		KR491796	KR491891
*Eigenmannia cf. virescens 4254*	LBP 4254	KR260151	KR491732	KR491590		KR491797	
*Eigenmannia vicentespelaea 62040*	LBP 62040	KR260152	KR491731	KR491592		KR491798	KR491892
*Eigenmannia virescens 36963*	MUSM 36963	KR260153	KR491734	KR491591		KR491799	KR491893
*Eigenmannia virescens 45735*	LBP 45735	KR260154	KR491730	KR491594			KR491894
*Eigenmannia virescens 29571*	LBP 29571	KR260155	KR491727	KR491587		KR491800	KR491895
*Rhabdolichops cf. stewarti 41406*	LBP 41406	KR260156	KR491724	KR491585		KR491801	KR491896
*Rhabdolichops cf. stewarti 49295*	LBP 49295	KR260157	KR491725	KR491584		KR491802	KR491897
*Rhabdolichops jegui 189017*	ANSP 189017	KR260158	KR491737	KR491583		KR491803	KR491898
*Sternopygus macrurus 39502*	MUSM 39502	KR260159	KR491722	KR491580		KR491804	KR491899
*Sternopygus macrurus 37350*	LBP 37350	KR260160	KR491723	KR491581		KR491805	KR491900
*Sternopygus xingu 19643*	LBP 19643	KR260161	KR491721	KR491582		KR491806	KR491901
*Sternopygus dariensis QG*	GQ-14916			KJ135110			
*Orthosternarchus tamandua QG*	GQ-36682	U15235					
*Sternarchorhamphus muelleri 182579*	ANSP 182579	KR260162	KR491636	KR491526		KR491807	KR491902
*Adontosternarchus sachsi 188863*	LBP 19126	KR260163	KR491638	KR491531		KR491808	KR491903
*Adontosternarchus balaenops 182572*	UFRGS 14826	KR260164	KR491637	KR491528		KR491809	KR491904
*Adontosternarchus clarkae 182580*	ANSP 182580	KR260165	KR491640	KR491529		KR491810	KR491905
*Adontosternarchus devenanzii 19126*	LBP 19126	KR260166	KR491639	KR491532		KR491811	KR491906
*Adontosternarchus nebulosus 14826*	UFRGS 14826	KR260167	KR491641	KR491530		KR491812	KR491907
*Parapteronotus hasemani 12797*	LBP 12797	KR260168	KR491642	KR491535		KR491813	
*Parapteronotus hasemani 178360*	ANSP 178360	KR260169	KR491643	KR491534		KR491814	KR491908
*Apteronotus albifrons 16150*	LBP 16150	KR260170	KR491647	KR491536		KR491815	KR491909
*Apteronotus albifrons 36939*	MUSM 36939	KR260171	KR491646	KR491537	KR491744	KR491816	KR491910
*Apteronotus albifrons 44716*	LBP 44716	KR260172	KR491648	KR491538		KR491817	KR491911
*Apteronotus caudimaculosus 43246*	LBP 43246	KR260173	KR491645	KR491525			KR491912
*Apteronotus leptorhynchus 190772*	ANSP 190772	KR260174	KR491644	KR491533		KR491818	KR491913
*Platyurosternarchus crypticus 179153*	ANSP 179153	KR260175	KR491649	KR491539		KR491819	KR491914
*Platyurosternarchus macrostomus 182522*	ANSP 182522	KR260176	KR491650	KR491540		KR491820	KR491915
*Sternarchorhynchus mormyrus 182583*	ANSP 182583	KR260177	KR491652	KR491553		KR491821	KR491916
*Sternarchorhynchus galibi 35866*	MUSM 35866	KR260178	KR491655	KR491559		KR491822	KR491917
*Sternarchorhynchus galibi 187155*	ANSP 187155	KR260179	KR491656	KR491558		KR491823	KR491918
*Sternarchorhynchus hagedornae 180637*	ANSP 180637	KR260180	KR491653	KR491556		KR491824	KR491919
*Sternarchorhynchus hagedornae 36892*	MUSM 36892	KR260181	KR491654	KR491557		KR491825	KR491920
*Sternarchorhynchus sp. 36838*	MUSM 36838	KR260182	KR491658	KR491561		KR491826	KR491921
*Sternarchorhynchus sp. 37135*	MUSM 37135	KR260183	KR491659	KR491562		KR491827	KR491922
*Sternarchorhynchus sp. 39556*	MUSM 39556	KR260184	KR491657	KR491563		KR491828	KR491923
*Sternarchorhynchus sp. 4066*	LBP 4066	KR260185	KR491662	KR491560		KR491829	KR491924
*Sternarchorhynchus sp. 57516*	LBP 57516	KR260186	KR491660	KR491552		KR491830	
*Sternarchorhynchus sp. T533*	ANSP T 533	KR260187	KR491663	KR491554		KR491831	
*Sternarchorhynchus starksi 47080*	MCP 47080	KR260188	KR491661	KR491555		KR491832	
*Sternarchella calhamazon 46987*	MCP 46987	KR260189	KR491669	KR491541		KR491833	KR491925
*Sternarchella terminalis 182576*	ANSP 182576	KR260190	KR491670	KR491542		KR491834	KR491926
*Apteronotus bonapartii 182585*	ANSP T 182585	KR260191	KR491673	KR491551		KR491835	
*Apteronotus bonapartii 37171*	MUSM 37171	KR260192	KR491675	KR491549		KR491836	KR491927
*Apteronotus bonapartii 36837*	MUSM 36837	KR260193	KR491676	KR491547		KR491837	KR491928
*Apteronotus ellisi 24040*	LBP 24040	KR260194	KR491674	KR491550		KR491838	KR491929
*Compsaraia samueli 182210*	ANSP 182210	KR260195	KR491651	KR491527		KR491839	KR491930
*Sternarchogiton labiatus QG*	GQ 189003		KR491665				
*Sternarchogiton nattereri 182208*	ANSP 182208	KR260196	KR491667	KR491545		KR491840	KR491931
*Sternarchogiton nattereri 37136*	MUSM 37136	KR260197	KR491668	KR491546		KR491841	KR491932
*Sternarchogiton porcinum 182212*	ANSP 182212		KR491671			KR491842	KR491933
*Sternarchogiton preto 57528*	LBP 57528	KR260198	KR491666	KR491544		KR491843	KR491934
*Sternarchogiton sp. 28120*	LBP 28120	KR260199	KR491664	KR491543		KR491844	KR491935
*Porotergus gimbelli 178277*	ANSP 178277	KR260200	KR491672	KR491548		KR491845	

*Abbreviations:* JSA: James S Albert uncatalogued; MUSM: Museo de Historia Natural, Universidad Nacional Mayor de San Marcos, Lima, Peru; LBP: Laboratório de Biologia de Peixes, Universidade Estadual Paulista, Botucatu, Brazil; MCP: Museu de Ciências e Tecnologia, Pontifícia Universidade Católica do Rio Grande do Sul, Porto Alegre, Brazil; GQ: GenBank; Brochu: Brochu, K., 2011. Molecular phylogenetics of the neotropical electric knifefish genus Gymnotus (Gymnotidae, Teleostei): biogeography and signal evolution of the trans-Andean species. Department of Biology. University of Toronto, Toronto, ON; LSUMZ: Louisiana State University Museum of Zoology, Baton Rouge, Louisiana, United States of America; Sullivan: Sullivan, J.P., 1997. A phylogenetic study of the Neotropical hypopomid electric fishes (Gymnotiformes: Rhamphichthyoidea). Department of Biology. Duke University, Durham, NC; UFRGS: Universidade Federal do Rio Grande do Sul, Porto Alegre, Brazil; ANSP: Academic of Natural Science of Philadelphia, Philadelphia, United States of America; UF: University of Florida, Florida Museum of Natural History, Gainesville, Florida, United States of America.

**Table 2:** Primers used for amplification and gene sequencing.

**Primer name**				**Primer sequence**			**Source**
mit-CYTB		F	GLUDG.L	5′ -	CGAAGCTTGACTTGAARAACCAYCGTTG	3′	[67]
mit-CYTB		R	CytbR	5′ -	CTCCGATCTTCGGATTACAAG	3′	[67]
mit-16S		F	16Sar	5′ -	CGCCTGTTTATCAAAAACAT	3′	[68]
mit-16S		R	16Sbr	5′ -	CCGGTCTGAACTCAGATCACGT	3′	[68]
mit-COI		F	BOL-COIfishF1	5′ -	TCAACYAATCAYAAAGATATYGGCAC	3′	[69]
mit-COI		R	BOL-COIfishR1	5′ -	ACTTCYGGGTGRCCRAARAATCA	3′	[69]
nuc-RAG2	External	F	RAG2F1	5′ -	TTTGGRCARAAGGGCTGGCC	3′	[70]
nuc-RAG2	External	R	RAG2R6	5′ -	TGRTCCARGCAGAAGTACTTG	3′	[70]
nuc-RAG2	Internal	F	RAG2GY-F	5′ -	ACAGGCATCTTTGGKATTCG	3′	[5]
nuc-RAG2	Internal	R	RAG2-GY-R	5′ -	TCATCCTCCTCATCTTCCTC	3′	[5]
nuc-RAG1	External	F	RAG12510F	5′ -	TGGCCATCCGGGTMAACAC	3′	[71]
nuc-RAG1	External	R	RAG14090R	5′ -	CTGAGTCCTTGTGAGCTTCCATRAAYTT	3′	[71]
nuc-RAG1	Internal	F	RAG1b2535F	5′ -	AGCCAGTACCATAAGATGTA	3′	[71]
nuc-RAG1	Internal	R	RAG1b4078R	5′ -	TGAGCCTCCATGAACTTCTGAAGRTAYTT	3′	[71]
nuc-ZIC1		F	Zic 1 F9	5′ -	GGACGCAGGACCGCARTAYC	3′	[71]
nuc-ZIC1		R	Zic 1 R967	5′ -	CTGTGTGTGTCCTTTTGTGRATYTT	3′	[71]
