# Supplementary material for: Data supporting phylogenetic reconstructions of the Neotropical clade Gymnotiformes
Source: Data Brief. 2016 Feb 6;7:23–59. doi: 10.1016/j.dib.2016.01.069 (PMC4761620; doi:10.1016/j.dib.2016.01.069)
Supplement: Supplementary file 1 — Supplementary material [file mmc1.zip › Supplementary material/Material_examined .docx]

***Material examined (see references in the main text)***

Outgroups - *Carassius auratus*: Uncatalogued aquarium specimen, *Cyphocharax festivus*: MUSM 33743, *Erythrinus erythrinus*: MUSM 33720, *Serrasalmus rhombeus*: MUSM 40906, *Charax tectifer*: MUSM 33862, *Dianema longibarbis*: MUSM 39374, *Brachyplatystoma juruense*: MUSM 39376, *Pseudostegophilus nemurus*: MUSM 40386, *Pterygoplichthys multiradiatus*: Uncatalogued aquarium specimen.

Gymnotidae - *Electrophorus electricus*: MUSM 39371, *Gymnotus pantherinus*: MCP 18108, MCP 20666, LBP 1232 (tissue 11144), *Gymnotus jonasi*: INPA 13507, LBP 6997 (tissue 34047), *Gymnotus stenoleucus*: MZUSP 43356, AMNH 59047, *Gymnotus coropinae*: INPA 18186, MCP 30682, MZUSP 75186, LBP 9280 (tissue 43746), *Gymnotus coatesi*: UMMZ 205149, AMNH 12624, FMNH 103345, *Gymnotus javari*: FMNH 103342, MCZ 60005, MUSM 14481, *Gymnotus pedanopterus*: INPA 6398, INPA 9742, AMNH 58651, *Gymnotus anguillaris*: MCZ 31219, UMMZ 190413, *Gymnotus cataniapo*: INPA 9808, AMNH 58650, *Gymnotus cylindricus*: AMNH 1358, *Gymnotus maculosus*: UMMZ 158451, UMMZ 190783, *Gymnotus panamensis*: CAS 217109, *Gymnotus henni*: FMNH 56793, ICNMHN 96, *Gymnotus tigre*: UF 25552, *Gymnotus curupira*: INPA 60609, MZUSP 75146, *Gymnotus obscurus*: MZUSP 75153, *Gymnotus pantanal*: UF 82146, LBP 6654 (tissue 39371), *Gymnotus chaviro*: MUSM 33714, *Gymnotus varzea*: MZUSP 60601, *Gymnotus omarorum*: AMNH 239656, *Gymnotus mamiraua*: INPA 9962, *Gymnotus RS1*: LBP 4762 (tissue 25550), *Gymnotus sylvius*: UMMZ 234347, LBP 7626 (tissue 36021), *Gymnotus sp RS2*: University of Louisiana uncat. 2008.03.11.19, LBP 8026 (tissue 37726), *Gymnotus bahianus*: MNRJ 4188, *Gymmnotus carapo*: USNM 225286, UF 37030, INPA 6378, LBP 5600 (tissue 27325), *Gymnotus ucamara*: UF 126182, *Gymnotus arapaima*: INPA 6387, MZUSP 75165, MZUSP 75176, *Gymnotus ardilai*: [55], *Gymnotus choco*: CAS 72192, FMNH 56794.

Hypopomidae - *Akawaio penak*: [6], *Hypopomus artedi*: UMMZ 187500, UMMZ 215603, *Microsternarchus bilineatus*: MCP 41061, MCP 41064, LBP 7006 (tissue 34063); *Racenisia fimbriipinna*: WRGC 12.120334, *Brachyhypopomus beebei*: MUSM 39375 (tissue 39375), FMNH 102281, *Brachyhypopomus bombilla* UFRGS 9274, *Brachyhypopomus brevirostris*: WRGC 01260193, LBP 16705 (tissue 16705), *Brachyhypopomus bullocki*: AUM 40160; *Brachyhypopomus draco*: UFRGS 9776, UFRGS 16267 (tissue 16267), *Brachyhypopomus gauderio*: UFRGS 9579, *Brachyhypopomus occidentalis*: ANSP 163176, AUM 40160, USNM 225286, *Brachyhypopomus bennetti*: UF 128875, *Brachyhypopomus* sp. n. “ele”. MUSM 37095, *Brachyhypopomus* sp. n. “pal”: USNM 270692, *Brachyhypopomus diazi*: Paratypes MZUSP 43131, *Brachyhypopomus pinnicaudatus*: UMMZ 204297, UMMZ 204632, *Procerusternarchus pixuna*: [22], *Racenisia fimbriipinna*: Paratypes MBUCV 07127.

Rhamphichthyidae - *Hypopygus cryptogenes*: MZUSP 24961, INPA 29466, *Hypopygus lepturus*: MCP 41121, LBP bb9278 (tissue 43739), *Hypopygus neblinae*: ANSP 169660, UF 148540, *Steatogenys duidae*: MCP 41126, LBP 7007 (tissue 34068), *Steatogenys elegans*: MCP 24290, LBP 3086 (tissue 19728), *Iracema caiana*: MZUSP 49205, *Gymnorhamphichthys bogardusi*: ANSP 187340, *Gymnorhamphichthys bogardusi*: ANSP 180949, ANSP 180972, MCP 15161, MZUSP 88589, MZUSP 97147, *Gymnorhamphichthys britskii*: FMNH 108545, MZUSP 59739, LBP 9729 (tissue 45898), *Gymnorhamphichthys hypostomus*: MCP 26580, *Gymnorhamphichthys rondoni*: FMNH 116884, INPA 9384, INPA 11598, MCP 30373, MCP 39906, MCP 40189, MZUSP 53631, MZUSP 96755, SU 67918, UF 19895, UF 19898, UF 36602, *Gymnorhamphichthys rondoni*: ANSP 185046, *Gymnorhamphichthys rosamariae*: FMNH 114672, MCP 24359, MCP 24872, MCP 24877, *Rhamphichthys apurensis*: ANSP 162300, LBP 10226 (tissue 43111), *Rhamphichthys* drepanium: INPA 17682, MZUSP 59297. UF 78066, *Rhamphichthys hahni*: *Rhamphichthys lineatus*: FMNH 1146850, INPA 15827, MCP 33457, LBP 3096 (tissue 19226), *Rhamphichthys marmoratus*: MZUSP 44493, LBP 9069 (tissue 42545), *Rhamphichthys rostratus*: MCP 27750, MZUSP 17645.

Sternopygidae - *Distocyclus conirostris*: UF uncat. 21.020999, *Archolaemus blax*: INPA 18451, *Eigenmannia virescens*: NRM 23210, NRM 45210, UF 78205, *Eigenmannia macrops*: MUSM 36873, UF 78117, UF 80887, MZUSP 29989, LBP 8853 (tissue 44284), *Eigenmannia vicentespelaea*: LBP 15289, *Rhabdolichops stewarti*: Holotype ANSP 158678, MBUC 7541, *Rhabdolichops jegui*: [56], *Rhabdolichops caviceps*: [14], *Rhabdolichops eastwardi*: LBP 5755, FMNH 100748, *Sternopygus astrabes*: AMNH 58643, INPA 6390, *Sternopygus macrurus*: MCNG 3733, UF 80862, MUSM 39502, LBP 7977 (tissue 37350), *Sternopygus xingu*: UMMZ 228961, LBP 2986 (tissue 19643), *Sternopygus dariensis*: NRM 27745.

Apteronotidae - *Orthosternarchus tamandua*: UF 116562, *Sternarchorhamphus muelleri*: LBP 15245, *Adontosternarchus sachsi*: FMNH 100742, UF 131125, *Adontosternarchus balaenops*: UF 116559, UF123451, UF126099, *Adontosternarchus clarkae*: UF 128993, UF 131126, *Adontosternarchus devenanzii*: UMMZ 228972, *Adontosternarchus nebulosus*: [57], *Parapteronotus hasemani*: UF116563, UF 129334, *Apteronotus albifrons*: UF 29921, MUSM 35762, MUSM 36939, *Apteronotus cuchillejo*: UF 25559, UF 25446, UF 30736, *Apteronotus caudimaculosus*: AMNH 222453, *Apteronotus leptorhynchus*: UF 33905, *Apteronotus eschmeyeri*: [58]. Osteological characters for this species were coded from holotype CAS 72115 taken from the California Academy of Sciences Ichthyology Primary Types Imagebase, *Megadonognathus cuyuniense*: Paratypes MCNG 10956, *Apteronotus magdalenensis*: Paratype USNM 39749, *Apteronotus cuchillo*: UF 25458, *Platyurosternarchus crypticus*: [59], *Platyurosternarchus macrostomus*: UF 116564, *Sternarchorhynchus mormyrus*: MZUSP 50187, MBUCV 8994, *Sternarchorhynchus oxyrhynchus*: UF 116577, *Sternarchorhynchus galibi*: [60], *Sternarchorhynchus hagedornae*: MUSM 36838, MUSM uncat. Los Amigos 401, *Sternarchorhynchus starksi*: MUSM 33844, *Pariosternarchus amazonensis*: ANSP 192996, *Sternarchella calhamazon* USNM 373113, MUSM 45230, MUSM 45231, USNM 373093, USNM 375373, *Sternarchella orinoco* USNM 228727, USNM 228738, USNM 228739, USNM 228740, *Sternarchella orthos* FMNH 102104, USNM 228722, USNM 228725, *Sternarchella schotti*: FMNH 54565, FMNH 115219, UF 26079, UF 116570, *Sternarchella sima*: AMNH 3864, Paratype, ANSP 192107, ANSP 192108, USNM 373112, USNM 373318, *Sternarchella terminalis*: FMNH 115218, FMNH 115251, FMNH 115230, MUSM 45241, MUSM 45236, USNM 373009, USNM 373232, USNM 373316, *Magosternarchus duccis*: ANSP 192995, UF 116561, USNM 337449, *Magosternarchus raptor*: UF 116762, USNM 337448, *Apteronotus bonapartii*: AU 23713 (6, 1 C&S), MUSM uncat, *Apteronotus ellisi*: LBP 4333, *Compsaraia compsa*: ANSP 163033, ANSP 165223, ANSP 163033, *Sternarchogiton nattereri*: UMMZ 233253, UF 116571, MUSM uncat., MUSM 37136, *Compsaraia samueli*: UF 122826, *Sternarchogiton labiatus*: [61], *Sternarchogiton porcinum*: MUSM uncat. Los Amigos 423, *Sternarchogiton preto*: UF 122827, UF 122828, *Porotergus gimbeli*: UMMZ 233253, FMNH 54327.
