# Supplementary material for: Data supporting phylogenetic reconstructions of the Neotropical clade Gymnotiformes
Source: Data Brief. 2016 Feb 6;7:23–59. doi: 10.1016/j.dib.2016.01.069 (PMC4761620; doi:10.1016/j.dib.2016.01.069)
Supplement: Supplementary file 1 — Supplementary material [file mmc1.zip › Supplementary material/Morphological_Matrix.docx]

*Carassius auratus* 040213

100000020000000000000000000000000001?00010?0??0????????????000000000000000000000000000001000000000000000000000000001?000000000000000000000000000000000000000000000000000000000000?????0?0100000000000000000000000000000??0000??

*Cyphocharax festivus* 33743

100000000000000000000000000000000000000000000000100000000000000000000000000000000000000100000000000000000?0000000001?000000000000000000000000000000000000000000000000000000000000?????0?0000000000000000000000000000000??0000??

*Erythrinus erythrinus* 33720

100000000000000000000000000000000000000000000000100000000000000000000000000000000000000100000000000000000?0000000001?000000000000000000000000000000000000000000000000000000000000?????0?0000000000000000000000000000000??0000??

*Serrasalmus rhombeus* 33812

00000000000000000000000000000000000000000000000010000000000000000000000000000000000000011000000000000000000000000001?000000000000000000000000000000000000000000000000000000000000?????0?0000000000000000000000000000000??0000??

*Charax tectifer* 33862

000000000000000000000000000000000000000000000000100000000000000000000000000000000000000100000000000000000?0000000001?000000000000000000000000000000000000000000000000000000000000?????0?0000000000000000000000000000000??0000??

*Dianema longibarbis* 39374

010000020000100000000000000012?00000000011?00001000000000001000000000000000100000000000000000100000100100?0010001001?100000001000000000010210000100000000000000000100000000000000?????0?0000000000000000000000000000000??0000??

*Brachyplatystoma juruense* 39376

010000020000100000000000000012?00000000011000001000000000001000000000000000100000000000000000100000100110?0010001001?100000001000000000010210000100000000000000000100000000000000?????0?0000000000000000000000000000000??0000??

*Pseudostegophilus nemurus* 33774

100000020000100000000000000012?00000000011?00001000000000001000000000000000100000000000000000100000100110?0010001000?000000001000000000010210000100000000000000000100000000000000?????0?0000000000000000000000000000000??0000??

*Pterygoplichthys multiradiatus* 39367

010000020000100000000000000012?00000000011?0000100000000000100000000000000010000000000000000010000010011000010001000?000000001000000000010210000100000000000000000100000000000000?????0?0000000000000000000000000000000??0000??

*Electrophorus electricus* 39371

101001000000110000000000000012?00000000010100001000000000001000100000001010100100001000000000000000100100111111110?00101100011100000000011000000100100000000100000001100110000000?????0?110400001103010000?20000010001320000001

*Gymnotus pantherinus* 11144

1010001101101100000000000000100011001000110000210000000000010001011100110101001000010010001100000001001001111111???0???1110011100000000010001010100100000000010000001000110000000?????0?110200001112000010010000010011110000000

*Gymnotus pantherinus* 24536

1010001101101100000000000000100011001000110000210000000000010001011100110101001000010010001100000001001001111111???0???1110011100000000010001010100100000000010000001000110000000?????0?110200001112000010010000010011110000000

*Gymnotus pantherinus* 31531

1010001101101100000000000000100011001000110000210000000000010001011100110101001000010010001100000001001001111111???0???1110011100000000010001010100100000000010000001000110000000?????0?110200001112000010010000010011110000000

*Gymnotus jonasi* 34047

101000110110110100000000000?100011101000110000210000000000010001011100110101001000010010001100000001001001111111???0???1110011100000000010001010100100000000010000001000110000000?????0?110200001112000010010000010011110000000

*Gymnotus jonasi* GQ

101000110110110100000000000?100011101000110000210000000000010001011100110101001000010010001100000001001001111111???0???1110011100000000010001010100100000000010000001000110000000?????0?110200001112000010010000010011110000000

*Gymnotus stenoleucus* GQ

101000110110110100000000000?100011001000110000210000000000010001011100110101001000010010001100000001001001111111???0???1110011100000000010001010100100000000010000001000110000000?????0?110200001112000010010000010011110000000

*Gymnotus coropinae* 43746

1010001101101101000000000000100011101000110000210000000000010001011100110101001000010010001100000001001001111111???0???1110011100000000010001010100100000000010000001000110000000?????0?110200001112000010010000010011110000000

*Gymnotus coropinae* 7161

1010001101101101000000000000100011101000110000210000000000010001011100110101001000010010001100000001001001111111???0???1110011100000000010001010100100000000010000001000110000000?????0?110200001112000010010000010011110000000

*Gymnotus coatesi* GQ

101000110110110100000000000?100011101000110000210000000000010001011100110101001000010010001100000001001001111111???0???1110011100000000010001010100100000000010000001000110000000?????0?110200001113000010010000010011110000000

*Gymnotus javari* GQ

101000110110110100000000000?100011001000110000210000000000010001011100110101001000010010001100000001001001111111???0???1110011100000000010001010100100000000010000001000110000000?????0?110200001113000010010000010011110000000

*Gymnotus pedanopterus* GQ

101000110110110100000000000?100011001000110000210000000000010001011100110101001000010010001100000001001001111111?000???1110011100000000010001010100100000000010000001000110000000?????0?110300001113000010010000010011110000000

*Gymnotus cf anguillaris* GQ (coded as *G*. *anguillaris*)

101000110110110100000000000?100011001000110000210000000000010001011100110101001000010010001100000001001001111111?000???1110011100000000010001010100100000000010000001000110000000?????0?110300001112000010010000010011110000000

*Gymnotus anguillaris* GQ

101000110110110100000000000?100011101000110000210000000000010001011100110101001000010010001100000001001001111111?000???1110011100000000010001010100100000000010000001000110000000?????0?110300001113000010010000010011110000000

*Gymnotus cylindricus* 1201

1010001101101100000000000000100011000000110000210000000000010001011100110101001000010010001100000001001001111111?000???1100011100000000010001010100100000000010000000000110000000?????0?110200001112000000010000010011110000001

*Gymnotus cylindricus* GQ

1010001101101100000000000000100011000000110000210000000000010001011100110101001000010010001100000001001001111111?000???1100011100000000010001010100100000000010000000000110000000?????0?110200001112000000010000010011110000001

*Gymnotus maculosus* 8126 – “Brochu, 2011”

101000110110110000000000000?100011000000110000210000000000010001011100110101001000010010001100000001001001111111?000???1100011100000000010001010100100000000010000000000110000000?????0?110200001112000000010000010011110000001

*Gymnotus panamensis* 8021 – “Brochu, 2011”

101000110110110000000000000?100011000000110000210000000000010001011100110101001000010010001100000001001001111111?00????1110011100000000010001010100100000000010000000000110000000?????0?110200001112000000010000010011110000000

*Gymnotus henni* 8231 – “Brochu, 2011”

101000110110112200000000000?100011100100110000210000000000010001011100110101001000010010001100000001001001111111???????1110011100000001010001010100100000000010000000000110000000?????0?110301001113000000010000010011110000000

*Gymnotus tigre* 060406

101000110110112200000000000?100011100100110000210000000000010001011100110101001000010010001100000001001001111111???0???1110011100000001010001010100100000000010000000000110000000?????0?110301001113000000010000010011110000000

*Gymnotus curupira* GQ

101000110110111200000000000?100011002000110000210000000000010001011100110101001000010010001100000001001001111111???0???1110011100000000010001010100100000000010000000000110000000?????0?110301001112000000010000010011110000000

*Gymnotus obscurus* GQ

101000110110111200000000000?100011102000110000210000000000010001011100110101001000010010001100000001001001111111???0???1110011100000001010001010100100000000010000000000110000000?????0?110301001112000000010000010011110000000

*Gymnotus pantanal* 31928

101000110110111200000000000?100011002000110000210000000000010001011100110101001000010010001100000001001001111111???0???1110011100000001010001010100100000000010000000000110000000?????0?110301001112000000010000010011110000000

*Gymnotus pantanal* 32017

101000110110111200000000000?100011002000110000210000000000010001011100110101001000010010001100000001001001111111???0???1110011100000001010001010100100000000010000000000110000000?????0?110301001112000000010000010011110000000

*Gymnotus chaviro* 39364

101000110110111200000000000?100011002000110000210000000000010001011100110101001000010010001100000001001001111111???0???1110011100000001010001010100100000000010000000000110000000?????0?110301001112000000010000010011110000000

*Gymnotus varzea* GQ

101000110110111200000000000?100011102000110000210000000000010001011100110101001000010010001100000001001001111111???0???1110011100000001010001010100100000000010000000000110000000?????0?110301001112000000010000010011110000000

*Gymnotus omarorum* 7093

000000110110111200000000000?100011102000110000210000000000010001011100110101001000010010001100000001001001111111?000???1110011100000001010001010100100000000110000000100110000000?????0?110301001112000000010000010011110000000

*Gymnotus mamiraua* GQ

000000110110111200000000000?100011002000110000210000000000010001011100110101001000010010001100000001001001111111?000???11100111000000010100010101001000000001100000001001100000000????0?110301001112000000010000010011110000000

*Gymnotus* sp. “ITAP” or “RS2” new species 25550

000000110110111200000000000?100011102000110000210000000000010001011100110101001000010010001100000001001001111111?000???11100111000000010100010101001000000001100000001001100000000????0?110301001112000000010000010011110000000

*Gymnotus sylvius* 36021

000000110110111200000000000?100011102000110000210000000000010001011100110101001000010010001100000001001001111111?000???11100111000000010100010101001000000001100000001001100000000????0?110301001112000000010000010011110000000

*Gymnotus* sp. “IGUA” or “RS1” new species 14044

000000110110111200000000000?100011102000110000210000000000010001011100110101001000010010001100000001001001111111?000???11100111000000010100010101001000000001100000001001100000000????0?110301001112000000010000010011110000000

*Gymnotus* sp. “ITAP” or “RS2” new species 37726

000000110110111200000000000?100011102000110000210000000000010001011100110101001000010010001100000001001001111111?000???11100111000000010100010101001000000001100000001001100000000????0?110301001112000000010000010011110000000

*Gymnotus bahianus* 7245 – “Brochu, 2011”

000000110110111200000000000?100011102000110000210000000000010001011100110101001000010010001100000001001001111111?000???11100111000000010100010101001000000001100000001001100000000????0?110301001112000000010000010011110000000

*Gymnotus carapo* 36951

00000011011011120000000000001000110&102000110000210000000100010001011100110101001000010010001100000001001001111111?000???11100111000000010100010101001000000001100000001001100000000????0?110301001112000000010000010011110000000

*Gymnotus carapo* 27325

00000011011011120000000000001000110&1020001100002100000001000100010111001101010010000100100011000000010010011111111000???11100111000000010100010101001000000001100000001001100000000????0?110301001112000000010000010011110000000

*Gymnotus carapo* 32294

00000011011011120000000000001000110&1020001100002100000001000100010111001101010010000100100011000000010010011111111000???11100111000000010100010101001000000001100000001001100000000????0?110301001112000000010000010011110000000

*Gymnotus carapo* 35859

00000011011011120000000000001000110&102000110000210000000100010001011100110101001000010010001100000001001001111111?000???11100111000000010100010101001000000001100000001001100000000????0?110301001112000000010000010011110000000

*Gymnotus ucamara* GQ

000000110110111200000000000?100011002000110000210000000000010001011100110101001000010010001100000001001001111111?000???11100111000000010100010101001000000001100000001001100000000????0?110301001112000000010000010011110000000

*Gymnotus arapaima* GQ

000000110110111200000000000?10001110200011000021000000010001000?011100110101001000010010001100000001001001111111???0???111001110000000101000101010?1000000001100000001001100000000????0?11030100111200000001000?010011110000000

*Gymnotus ardilai* 8175 – “Brochu, 2011”

000000110110111200000000000?100011002000110000210000000000010001011100110101001000010010000000000001001001111111?00????111001110000000101000101010010000000011000000?1001100000000????0?110301001112000000010000010011110000000

*Gymnotus choco* 8209 – “Brochu, 2011”

000000110110111200000000000?100011002000110000210000000000010001011100110101001000010010000000000001001001111111?00????11100111000000010100010101001000000001100000001001100000000????0?110301001112000000010000010011110000000

*Akawaio penak* GQ

10101012?000110000000000001?10000??1?01?1????????010000??00?0????????????????0???????????????????????0??????1111???????01000?11100000100001??????00???00100??0??????1100110?????1000??0?11020000???00????????1?1?0101111000000?

*Hypopomus artedi* GQ

101010120000110000000000001?10000001?01?11000101?0000000000101100100?10100010000100?0010000000110111001?0111111111?????0110011110000010000111000100100000000100100000000110000001000100?110200001100100000000111101011110000000

*Microsternarchus bilineatus* 34063

101000120000110000000000001?11000001?01?11200101?0010000000101100100?1011?010000100?001000000011011100100111111111?00001100011110000010000001000100100000000100100001?10110000001000100?110200001100100000000111101011110000000

*Microsternarchus bilineatus* 50417

101000120000110000000000001?11000001?01?11200101?0010000000101100100?1011?010000100?001000000011011100100111111111?00001100011110000010000001000100100000000100100001?10110000001000100?110200001100100000000111101011110000000

*Racenisia fimbriipinna* GQ

101000120000110000000000001?11000001?01?11200101?0010000000101100100?10?1?010000100?0010000000110111001?0111111111?????1100011110000010000101000100100000000110100001?10110000001000100?110200001101000000000111111011110000000

*Procerusternarchus pixuna*

101000120000110000000000001?10000001?01?11200101?0010110?00101100100?1011?010000100?0010000000110111001?0??11111???????110001111000001000000100010010000200011010000011?110?0?0010001?0?1102000011?00&100000?001?1?01011110000000

*Brachyhypopomus brevirostris* GQ

101000100000110010000000001110000001?01?11000101?0000000000101100100?101010100001001001000000011011100100111111111?00001110011110000010000111000100100000000100100000110111000001000100?110300001100100000000111121111110000000

*Brachyhypopomus bullock* - “Sullivan, 1997”

101000100000110010000000001110000001?01?11000001?0000000000101100100?101010100001001001000000011011100100111111111100001110011110000010000111000100100000000100100000110111000001000100?110300001100100000000111101111110000000

*Brachyhypopomus* sp. “ROY” new species – “Sullivan, 1997”

101000100000110000000000001110000001?01?11000001?0000000000101100100?101010100001001001000000011011100100111111111100001110011110000010000111000100100000000100100000110111000001000100?110300001100100000000111101011110000000

*Brachyhypopomus* sp. “PAL” new species GQ

101000100000110000000000001110000001?01?11000001?0000000000101100100?101010100001001001000000011011100100111111111100001110011110000010000111000100100000000100100001110111000001000100?110300001100100000000111101011110000001

*Brachyhypopomus* sp. “PAL” new species GQ

101000100000110000000000001110000001?01?11000001?0000000000101100100?101010100001001001000000011011100100111111111100001110011110000010000111000100100000000100100001110111000001000100?110300001100100000000111101011110000001

*Brachyhypopomus diazi* GQ

101000100000110000000000001110000001?01?11000001?0000000000101100100?101010100001001001000000011011100100111111111100001110011110000010000111000100100000000100100001110111000001000100?11030000110010000000011110101111000000?

*Brachyhypopomus occidentalis* 1849

101000100000110000000000001110000001?01?11000001?0000000000101100100?101010100001001001000000011011100100111111111100001110011110000010000111000100100000000100100001110111000001000100?110300001100100000000111101011110000000

*Brachyhypopomus pinnicaudatus* GQ

101000100000110000000000001110000001?01?11000001?0000000000101100100?101010100001001001000000011011100100111111111100000110011110000010000111000100100000000100100001110111000001000100?110300001100000000000111121111110000000

*Brachyhypopomus brevirostris* 16705

101000100000110000000000001110000001?01?11000001?0000000000101100100?101010100001001001000000011011100100111111111100000110011110000010000111000100100000000100100001110111000001000100?110300001100000000000111121111110000000

*Brachyhypopomus draco* 16267

101000100000110000000000001110000001?01?11000001?0000000000101100100?101010100001001001000000011011100100111111111100000110011110000010000111000100100000000100100001110111000001000100?110300001100000000000111101111110000000

*Brachyhypopomus beebei* 39375

101000100000110000000000001110000001?01?11000001?0000000000101100100?101010100001001001000000011011100100111111111100000110011110000010000111000100100000000100100001110111000001000100?110300001100000000000111101011110000000

*Hypopygus lepturus* 43739

000100110101110010000000001110010001?01?11000001?0000110000101100100?1111?0100001001011000000010011100100111111110?00001100011110000010000001010110110002000000101001000110000001000100?111100001100200000000100021011110101000

*Hypopygus neblinae* 14841

000100110101110010000000001?10010001?01?11000001?000???????101100100?1111?010000100101100000001001110010011111?1?0?0???1100011110000010000001010110110002000000101001000110?000010?01?0?11110000110020000000?100021011110101000

*Hypopygus cryptogenys*

000100110101110010000000001?10010001?01?11000001?0100110000101100100?1111?0100001001011000000010011100100111111110?00001100011110000010000101000100110002000000101001?00110000001000100?111100001100200000000100021011110101000

*Steatogenys duidae* 34068

000100110000110001000000001?10000001?01?11000001?0000110000101100100?0111?010000100101100000001001110010011111?1?0?0???1100011110000010000101010100110002000000101100110110?000010?01?0?111100001100200000000100021011110111000

*Steatogenys elegans* 182571

000100110000110001000000001110000001?01?11000001?0000110000101100100?0111?010000100101100000001001110010011111?1?0?0???1100011110000010000101010100110002000000101100110110?000010?01?0?111100001100200000000100021011110111000

*Steatogenys elegans* 19728

000100110000110001000000001110000001?01?11000001?0000110000101100100?0111?010000100101100000001001110010011111?1?0?0???1100011110000010000101010100110002000000101100110110?000010?01?0?111100001100200000000100021011110111000

*Gymnorhamphichthys britskii* 22012

001010121000110001000000001?12001001?01?11100001?01000000011111001001001000100001101000000000010011100100111111110?10001100111111100010001111000100100000000000100101000110000001100000?112221001100100000000101021011110100000

*Gymnorhamphichthys britskii* 45898

001010121000110001000000001?12001001?01?11100001?01000000011111001001001000100001101000000000010011100100111111110?10001100111111100010001111000100100000000000100101000110000001100000?112221001100100000000101021011110100000

*Gymnorhamphichthys rosamariae* 191142

001010121000110000000000001112001001?01?11100001?01000000011111001001001000100001101000000000010011100100111111110?10001100111111100010001111000100100000000000100101000110000001100000?112221001100100000000101021011110100000

*Gymnorhamphichthys hypostomus* 18063

001010121000110001000000001112001001?01?11100001?01000000011111001001001000100001101000000000010011100100111111110?10001100111111101010001111000100100000000000100101000110000001100000?112221001100100000000101021011110100000

*Gymnorhamphichthys bogardusi* 191143

001010121000110000000000001?12001001?01?11100001?01000000011111001001001000100001101000000000010011100100111111110?10001100111111101010001111000100100000000000100101000110000001100000?112221001100100000000101021011110100000

*Gymnorhamphichthys bogardusi* T09059

001010121000110000000000001?12001001?01?11100001?01000000011111001001001000100001101000000000010011100100111111110?10001100111111101010001111000100100000000000100101000110000001100000?112221001100100000000101021011110100000

*Gymnorhamphichthys rondoni* 179673

001010121000110000000000001?12001001?01?11100001?01000000011111001001001000100001101000000000010011100100111111110?10001100111111101010001111000100100000000000100101000110000001100000?112221001100100000000101021011110100000

*Gymnorhamphichthys petiti* 179685

001010121000110000000000001?12001001?01?11100001?01000000011111001001001000100001101000000000010011100100111111110?10001100111111101010001111000100100000000000100101000110000001100000?112221001100100000000101021011110100000

*Gymnorhamphichthys petiti* 11515

001010121000110000000000001?12001001?01?11100001?01000000011111001001001000100001101000000000010011100100111111110?10001100?11111101010001111000100100000000000100101000110000001100000?112221001100100000000101021011110100000

*Iracema caiana*

001010121000110010000000001?11001101?01?11000001?000000100111110010000011?0100001001000000000010011100100111111110?1000111011111110001000101100010010000???100010010000011000000110???0?1123210011?000000000010102101111010000?

*Rhamphichthys rostratus* GQ

001010121000110010000000001?11001101?01?11000001?00000010011111001000001000100001001000000000010011100100111111110?10001110111111100010000111000100100000001000101100000110000001100000?112401001100100000001101021011110100000

*Rhamphichthys rostratus* 187120

001010121000110010000000001?11001101?01?11000001?00000010011111001000001000100001001000000000010011100100111111110?10001110111111100010000111000100100000001000101100000110000001100000?112401001100100000001101021011110100000

*Rhamphichthys rostratus* T3954

001010121000110010000000001?11001101?01?11000001?00000010011111001000001000100001001000000000010011100100111111110?10001110111111100010000111000100100000001000101100000110000001100000?112401001100100000001101021011110100000

*Rhamphichthys rostratus* GQ

001010121000110010000000001?11001101?01?11000001?00000010011111001000001000100001001000000000010011100100111111110?10001110111111100010000111000100100000001000101100000110000001100000?112401001100100000001101021011110100000

*Rhamphichthys apurensis* 43111

001010121000110010000000001?11001101?01?11000001?00000010011111001000001000100001001000000000010011100100111111110?10001110111111100010000111000100100000001000101100000110000001100000?112401001100100000001101021011110100000

*Rhamphichthys apurensis* T9915

001010121000110010000000001?11001101?01?11000001?00000010011111001000001000100001001000000000010011100100111111110?10001110111111100010000111000100100000001000101100000110000001100000?112401001100100000001101021011110100000

*Rhamphichthys drepanium* – “Sullivan, 1997”

001010121000110010000000001111001101?01?11000001?00000010011111001000001000100001001000000000010011100100111111110?10001110111111100010000111000100100000001000101100000110000001100000?112401001100100000001101021011110100000

*Rhamphichthys hahni* 19226

001010121000110010000000001?11001101?01?11000001?00000010011111001000001000100001001000000000010011100100111111110?10001110111111100010000111000100100000001000101100000110000001100000?112401001100100000001101021011110100000

*Rhamphichthys lineatus* 116566

001010121000110000000000001?11001101?01?11000001?00000010011111001000001000100001001000000000010011100100111111110?10001110111111100010000111000100100000001000101100000110000001100000?112401001100100000001101021011110100000

*Rhamphichthys marmoratus* 42545

001010121000110010000000001?11001101?01?11000001?00000010011111001000001000100001001000000000010011100100111111110?10001110111111100010000111000100100000001000101100000110000001100000?112401001100100000001101021011110100000

*Japigny kirschbaum*

001100120000110001000000001?1000000010???????0?100000000100101???????????????000?????????0???????????0?????????1???????1110?1??????0??????1??????????????00???????11??10110?????10????0?1111100??????0???????10000101111100001?

*Distocyclus conirostris* 182573

0010001000000100000000000111100000001010111000010000000010010100000000000011100000110000000001001000001?0011111110?01101110011100000100010111000101100012000000100111010110111111011000?111310001000201100000100001011111000011

*Archolaemus blax* GQ

001000101000000000000000001?10000000101011100001000000001001010?00??00000011100000110000000001001000001?0011111110?????1110001??0000100?10111000101100010000000100111000110111111011000?111310001000100100000100001011111000011

*Eigenmannia virescens* 41404

001100100000010000010000011110000000101011100001000000001001011000000000001110000011000000000100100000100011111110101101110011100000100010111000100000010000000100111010110111111010000?111210001000201100000100001011111000011

*Eigenmannia macrops* 37145

001100120000010000000000011?100000001010111000010000000010010110000000000011100000110000000001001????0??00?????1???0???11100111000001000101110001000000100000001001????0110111??10????0?111210001000201100000100001011111000011

*Eigenmannia macrops* 44284

001100120000010000000000011?100000001010111000010000000010010110000000000011100000110000000001001????0??00?????1???0???11100111000001000101110001000000100000001001????0110111??10????0?111210001000201100000100001011111000011

*Eigenmannia cf. virescens* 4254

001100100000010000010000011110000000101011100001000000001001011000000000001110000011000000000100100000100011111110101101110011100000100010111000100000010000000100111010110111111010000?111210001000201100000100001011111000011

*Eigenmannia vicentespelaea* 62040

001100100000010000010000011?10000000101011100001000000001001011000000000001110000011000000000100100000100011111110101101110011100000100010111000100000010000000100111010110111111010000?111210001000201100000100001011111000011

*Eigenmannia virescens* 36963

001100100000010000010000011110000000101011100001000000001001011000000000001110000011000000000100100000100011111110101101110011100000100010111000100000010000000100111010110111111010000?111210001000201100000100001011111000011

*Eigenmannia virescens* 45735

001100100000010000010000011110000000101011100001000000001001011000000000001110000011000000000100100000100011111110101101110011100000100010111000100000010000000100111010110111111010000?111210001000201100000100001011111000011

*Eigenmannia virescens* 29571

001100100000010000010000011110000000101011100001000000001001011000000000001110000011000000000100100000100011111110101101110011100000100010111000100000010000000100111010110111111010000?111210001000201100000100001011111000011

*Rhabdolichops cf. stewarti* 41406

001100100000010000000000011?110000001010110001010000000010010110000000000011100000110111000001001000001?00?11?11???0???1110011100000100010111100100000010000000100111010110111111010010?111211001000201100000100001011111000011

*Rhabdolichops cf. stewarti* 49295

001100100000010000000000011?110000001010110001010000000010010110000000000011100000110111000001001000001?00?11?11???0???1110011100000100010111100100000010000000100111010110111111010010?111211001000201100000100001011111000011

*Rhabdolichops jegui* 189017

001100100000010000000000011?1100000010101100010?0000???????10110000000000011100000110111000001001000001?00?11?11???????1110011100000100010111100100000010000000100111010110111111010010?111211001000201100000100001011111000011

*Rhabdolichops caviceps*

001100110000010000000000011?110000001010110001010000000010010110000000000011100000110111000001001000001?00?11?11???0???1110011100000100010110000100000010000000100111010110111111010010?111211001000201100000100001011111000011

*Rhabdolichops eastwardi*

001100100000010000000000011111000000101011000101000000001001011000000000001110000011011100000100100000100011111110?01101110011100000100010110000100000010000000100111010110111111010010?111211001000201100000100001011111000011

*Sternopygus astrabes*

001000100000000001020000000?100000001010111000010000000010010100000100000011100000010001000001001000001000111111101?110111000110000010001011?000110100010000000100001000110111011010&10&100?111330001000000000000100001011101000011

*Sternopygus macrurus* 39502

0000001010000000000200000001100000001010111000010000000010010100000100000011100000010001000001001000001?00111111???0???111000110000010001011??001??1000100000001000010011101110110110&100?11133000100100000000&10100001011101000011

*Sternopygus macrurus* 37350

000000101000000000020000000?100000001010111000010000000010010100000100000011100000010001000001001000001?00111111???????111000110000010001011??001??1000100000001000010011101110110110&100?111330001001000000000100001011101000011

*Sternopygus xingu* 19643

000000101000000000020000000?100????01010111000010000000010010100000100000011100000010?0100000100100000??00111111???????1110?011000?01?0??????????????????00???????001??11101110?1011??0?111330001001000000000100001011101000011

*Sternopygus dariensis* QG

0000001010000000000?0000000?100????01010111000000000000010010100000?000?0011100000010?0100000100100000??001?1111???????1110?011000?01?0??????????????????00???????0?1??11101110?1011??0?11133000100?000000000100001011101000011

*Orthosternarchus tamandua* QG

001010121000110000000000001?11000000001011101101100000000001110000010001010101000001?00101110100000100101111111110??1111101?1111001000011101101111011010000100010000000011001100101101111123311110000000011001000100011?1000111

*Sternarchorhamphus muelleri* 182579

001010121000110000000000001111000000001011101001100000000001110000010001010101000001000101110100000100101111111110?01111101?1111001000011101101111011010000100010010000011001100101101111123311110001000011001000100011?1000111

*Adontosternarchus sachsi* 188863

001100100000110000000000001?10000001?01111100021?100000000010110001000110?0100001001100101110100000100100111111110?0111111001110001000011011101111011010100100011010100011001100101101101111110010002000100001000100011?1000110

*Adontosternarchus balaenops* 182572

001100100000110000000000001?10000001?01111100021?100000000010110001000110?01000010011001011101000001001001111111100?1111110011100010000110111011110110101001000110101000110?1100101101101111110010001000101001000100011?1000110

*Adontosternarchus clarkae* 182580

001100100000110000000000001110000001?01111100021?100000000010110001000110?01000010011001011101000001001001111111100?1111110011100010000110111011110110101001000110101000110?1100101101101111110010001000101001000100011?1000110

*Adontosternarchus devananzii* 19126

001100100000110000000000001?10000001?01111100021?100000000010110001000110?01000010011001011101000001001001111111100?1111110011100010000110111011110110101001000110101000110?1100101101101111110010001000101001000100011?1000110

*Adontosternarchus nebulosus* 14826

001100100000110000000000001?10000001?01111100021?100000000010110001000110?01000010011001011101000001001001111111100?1111110011100010000110111011110110101001000110101000110?1100101101101111110010001000101001000100011?1000110

*Parapteronotus hasemani* 12797

001011000000110000000000001?100000000010111001010000000000010111001000010001000000010001011101000001001?0111111110??11?111001110001000011101101111011010100100010010100011001100101001101111100010001000110001000100011?1000110

*Parapteronotus hasemani* 178360

001011000000110000000000001?100000000010111001010000000000010111001000010001000000010001011101000001001?0111111110?0???111001110001000011101101111011010100100010010100011001100101001101111100010001000110001000100011?1000110

*Apteronotus albifrons* 16150

00100102000011000010111000111000000000101110010110000000010101110010000100010000000100010010010000010010011111111010111111001110001000011101101111011010110100010010100011001100101101101111100010001000110001000000011?1000110

*Apteronotus albifrons* 36939

00100102000011000010111000111000000000101110010110000000010101110010000100010000000100010010010000010010011111111010111111001110001000011101101111011010110100010010100011001100101101101111100010001000110001000000011?1000110

*Apteronotus albifrons* 44716

00100102000011000010111000111000000000101110010110000000010101110010000100010000000100010010010000010010011111111010111111001110001000011101101111011010110100010010100011001100101101101111100010001000110001000000011?1000110

*Apteronotus cuchillejo*

001000020000110000101110001?100000000011111001010100000001010111001000011001000000010001001001000001001?0111111110?????111001110001000011101101111011010110100011?10110011001100101101101111100010001000110001000000011?100011?

*Apteronotus caudimaculosus* 43246

001001020000110000101110001?100000000010111001011000000001010111001000010001000000010001001001000001001001111111101?111111001110001000011101101111011010110100010010100011001100101101101111100010001000110001000000011?1000110

*Apteronotus leptorhynchus* 190772

001011020000110000101010001?1000000000101110010100000010010101110010000100010001000100010010010000010010011111111010111111001110001000011101101111011010110100010010100011001100101101101111100010000000110001000000011?1000110

*Apteronotus eschmeyeri*

001011020000110000101010001?10000000001011100101000000100101011100100001000100010001000100100100000100100111111110??111111001110001000011101101111011010110100010010100011001100101101101111100010000000110001000000011?1000110

*Megadontognathus cuyuniense*

001001020000110000001000001?10000000001011?00101000000100101011100100001000100000001000100100???0001001?0111111110?????1?10011??001000011101????11???????001000100?01000110011001011001011111000100000001?0001?00000011?1000110

*Apteronotus magdalenensis*

001010020000110000101010101?10?0000000101110010100000000?101011100100001000100010001000100?????????1001?0111111110?????110101110001000011101?????1?????????????????01?00110?1100101?0?10111110001000000?1?0001?00000011?100011?

*Apteronotus cuchillo*

001010020000110000101010101?1000000000101110010100000000?101011100100001000100010001000100?????????1001?0111111110?????1101?11100010000111???????1?????????????????01?00110011001011?1101111100010000000110001?00000011?100011?

*Platyurosternarchus crypticus* 179153

001011020000110000000000001?100000000011110010010000000000111110000100011?0100000000000101100100000100100111111110?01111101?01110010000111011?1111011010000100010010100011001100101100101123111010001000100001000100011?1000110

*Platyurosternarchus macrostomus* 182522

001011020000110000000000001?100000000011110010010000000000111110000100011?0100000000000101100100000100100111111110?01111101001110010000111011?1111011010000100010010100011001100101100101123111010001000100001000100011?1000110

*Sternarchorhynchus mormyrus* 182583

0010101010001100000000100011100000000011110000010000100000111110000100011?0100001000000101100000000100100111111110?01111101?1111001?00011111101110011010000100010010?000110011001011011011221110100010001?0001000100011?100011?

*Sternarchorhynchus oxyrhynchus*

001010101000110000000010001?100000000011110000010000100000111110000100011?0100001000000101100000000100100111111110?01111101?11110010000111111011100110100001000100101000110011001011011011221110100010001?0001000100011?1000110

*Sternarchorhynchus galibi* 35866

001010101?00110000000010001?100????0001111?0??0?????1??????1111??????????????????????????????????????0??01?????1???????11???????????0?0????????????????????????????????0110??1??10????10112211???????0???????1?00100011?100011?

*Sternarchorhynchus galibi* 187155

001010101?00110000000010001?100????0001111????0?????1??????1111??????????????????????????????????????0??01?????1???????11???????????0?0??????????????????00????????????0110??1??10????10112211???????0???????1?00100011?100011?

*Sternarchorhynchus hagedornae* 180637

0010101010001100000000100011100000000011110000010000100000111110000100011?01000010000001011000000001001?011?1?11???????110??111100100001111110111001101?0001000100101000110011001011011011221110100010001??001000100011?1000110

*Sternarchorhynchus hagedornae* 36892

0010101010001100000000100011100000000011110000010000100000111110000100011001000010000001011000000001001?011?1?11???????110??111100100001111110111001101?0001000100101000110011001011011011221110100010001??001000100011?1000110

*Sternarchorhynchus* sp. 36838

001010101000110000000010001110000000001111??0001000010000011111000010001100100001000000101100?000001001?01?????1???????11???????????0?0??????????????????00????????????0110??1??10????10112211???????0???????1000100011?100011?

*Sternarchorhynchus* sp. 37135

001010101000110000000010001110000000001111??00010000100000111110000100011?0100001000000101100?000001001?01?????1???????11???????????0?0??????????????????00????????????0110??1??10????10112211???????0???????1000100011?100011?

*Sternarchorhynchus* sp. 39556

001010101000110000000010001110000000001111??00010000100000111110000100011?0100001000000101100?000001001?01?????1???????11???????????0?0??????????????????00????????????0110??1??10????10112211???????0???????1000100011?100011?

*Sternarchorhynchus* sp. 4066

001010101000110000000010001110000000001111??00010000100000111110000100011?0100001000000101100?000001001?01?????1???????11???????????0?0??????????????????00????????????0110??1??10????10112211???????0???????1000100011?100011?

*Sternarchorhynchus* sp. 57516

001010101000110000000010001110000000001111??00010000100000111110000100011?0100001000000101100?000001001?01?????1???????11???????????0?0??????????????????00????????????0110??1??10????10112211???????0???????1000100011?100011?

*Sternarchorhynchus* sp. T533

001010101000110000000010001110000000001111??00010000100000111110000100011?0100001000000101100?000001001?01?????1???????11???????????0?0??????????????????00????????????0110??1??10????10112211???????0???????1000100011?100011?

*Sternarchorhynchus starksi* 47080

001010101000110000000010001110000000001111??00010000100000111110000100011?0100001000000101100?000001001?01?????1???????11???????????0?0??????????????????00????????????0110??1??10????10112211???????0???????1000100011?100011?

*Pariosternarchus amazonensis*

0010001200001100000000000111121000?00010111010011010000000010111001000010001000000010001111101000001101?01??1??1???????1110111100010100111?101111101111010010&100100?0100011001100101101101111110010001000110001000100011?100011?

*Sternarchella calhamazon* 46987

001100110000110000000000011?121000000000110100011010000000010101102000000001000000010001111101000001001?01?????1???0???111011110001001011101?1111101111010110001001010001100110010110110111111001000100011?001000100011?1000110

*Sternarchella terminalis* 182576

0011001000001100000000000111121000000000110100011010000000010101102000000001000000010001111101000001001?011?11111??0???11101111000100001110101111101111010110&10010010100011001100101101101111110010001000110001000100011?1000110

*Magosternarchus duccis*

001100010000110000000000011?111000000000110100011010000000010101102000000001000010000001111101000001001?0111111110?????111011110001000011101101111?11110101100011010100011001100101101101111110010001000110001000100011?1000110

*Magosternarchus raptor*

001011000000110000000000011?111000000000111010111010000000010101102000000001000010000001111101000001001?0111111110?????111011110001000011101101111?11110101100011010100011001100101101101111110010001000110001000100011?1000110

*Apteronotus bonapartii* 182585

0010010210001100001000000111111000000010111011111000000000010111001000010001000000000001011??100000110?0011?1111???0???111001110001000011101101111011010110100110010100011001100101101101111110010001000110001000000011?1000110

*Apteronotus bonapartii* 37171

0010010210001100001000000111111000000010111011111000000000010111001000010001000000000001011??100000110?0011?1111???0???111001110001000011101101111011010110100110010100011001100101101101111110010001000110001000000011?1000110

*Apteronotus* *bonapartii* 36837

001001021000110000100000011?111000000010111011111000000000010111001000010001000000000001011??100000110?0011?1111???????111001110001000011101101111011010110100110010100011001100101101101111110010001000110001000000011?1000110

*Apteronotus ellisi* 24040

0010010210001100001000000111111000000010111011?11000000000010111001000010001000000000001011??100000110?0011?1111???????111001110001000011101101111011010110100110010100011001100101101101111110010001000110001000000011?1000110

*Compsaraia compsa*

001011021000110000100001011?12100000001011101101100000000001011100100001000101000001000101100100000111100111111110?01111110011100010100111??101111011010110100111010100111001100101101101111110010001000110001000000011?1000110

*Compsaraia samueli* 182210

001011021000110000100001011?1210000000101110110110000000000101110010000100010100000?0001011001000001111?0111111110?????1110011100010100111??10?111011010110100111010100111001100101101101111110010001000110001000000011?1000110

*Sternarchogiton labiatus* 189003

00110012??00110000100000011?121????000111110111???00???????1011?????????????????????????????1????????0??01?????1???????1????????????0?0??1????????????????0?????????1???110?11?010????101111111??000200????001?00000011?1000110

*Sternarchogiton nattereri* 182208

001100120000110000100000011?12100000001111101111010000000001011100100001010100001001100101101100000110100111111110?0???111001110001000011101101111011010100100011000100111001100101100&1101111111010002000110001000000011?1000110

*Sternarchogiton nattereri* 37136

001100120000110000100000011?12100000001111101111010000000001011100100001010100001001100101101100000110100111111110?0???111001110001000011101101111011010100100011000100111001100101100&1101111111010002000110001000000011?1000110

*Sternarchogiton porcinum* 182212

001100120000110000100000011?1210000000111110111101000000???10111001000010101000010011001011011000001101?01?????1???????1????????????0?0??1????????????????0?????????1???110?110010????1011111110?00020001??001000000011?1000110

*Sternarchogiton preto* 57528

00110012000011000010000001111210000000111110111101000000???10111001000010101000010011001011011000001101?01?????1???????1????????????0?0??1????????????????0?????????1???110?110010????1011111110?00020001??001000000011?1000110

*Sternarchogiton* sp. 28120

001100120000110000100000011?1210000000111110111101000000???10111001000010101000010011001011011000001101?01?????1???????1????????????0?0??1????????????????0?????????1???110?110010????1011111110?00020001??001000000011?1000110

*Porotergus gimbeli* 178277

00100102000011000010000001111210000000111110110100000000?001011100100001010100001001100101??0?00???1101?0111111110?????111001110001000011101?????1????????0????????0100111001100101100101111111010001000110001000000011?1000110
