# Supplementary material for: Data supporting phylogenetic reconstructions of the Neotropical clade Gymnotiformes
Source: Data Brief. 2016 Feb 6;7:23–59. doi: 10.1016/j.dib.2016.01.069 (PMC4761620; doi:10.1016/j.dib.2016.01.069)
Supplement: Supplementary file 1 — Supplementary material [file mmc1.zip › Supplementary material/Scripts.docx]

######################################################

##PARTITIONFINDER – GARLI (ML) – GYMNOTIFORMES##

######################################################

## ALIGNMENT FILE ##

alignment = GYMN_matrixMolecular.phy;

## BRANCHLENGTHS: linked | unlinked ##

branch lengths = linked;

## MODELS OF EVOLUTION for PartitionFinder: all | RAxML | MrBayes | beast | <list> ##

## for PartitionFinderProtein: all protein | <list> ##

models = F81, K80, HKY, Tefé, TrN, K81, K81uf, TIMef, TIM, Tefé, TVM, SYM, GTR, F81+G, K80+G, HKY+G, TrNef+G, TrN+G, K81+G, K81uf+G, TIMef+G, TIM+G, TVMef+G, TVM+G, SYM+G, GTR+G;

# MODEL SELECCTION: AIC | AICc | BIC #

model selection = AICc;

## DATA BLOCKS: see manual for how to define ##

[data blocks]

Gene16S = 1-543;

GeneCOI_pos1 = 544-1202\3;

GeneCOI_pos2 = 545-1202\3;

GeneCOI_pos3 = 546-1202\3;

GeneCYTB_pos1 = 1203-2232\3;

GeneCYTB_pos2 = 1204-2232\3;

GeneCYTB_pos3 = 1205-2232\3;

GeneRag2_pos1 = 2233-3202\3;

GeneRag2_pos2 = 2234-3202\3;

GeneRag2_pos3 = 2235-3202\3;

GeneRag1_pos1 = 3203-4539\3;

GeneRag1_pos2 = 3204-4539\3;

GeneRag1_pos3 = 3205-4539\3;

GeneZic1_pos1 = 4540-5277\3;

GeneZic1_pos2 = 4541-5277\3;

GeneZic1_pos3 = 4542-5277\3;

## SCHEMES, search: all | greedy | cluster | cluster | user ##

[schemes]

search = greedy;

#user schemes go here if search=user. See manual for how to define.#

######################################################

##PARTITIONFINDER – MrBayes (BI) – GYMNOTIFORMES##

######################################################

## ALIGNMENT FILE ##

alignment = GYMN_matrixMolecular.phy;

## BRANCHLENGTHS: linked | unlinked ##

branch lengths = linked;

## MODELS OF EVOLUTION for PartitionFinder: all | RAxML | MrBayes | beast | <list> ##

## for PartitionFinderProtein: all protein | <list> ##

models = F81, K80, HKY, SYM, GTR, F81+G, K80+G, HKY+G, SYM+G, GTR+G;

# MODEL SELECCTION: AIC | AICc | BIC #

model selection = AICc;

## DATA BLOCKS: see manual for how to define ##

[data blocks]

Gene16S = 1-543;

GeneCOI_pos1 = 544-1202\3;

GeneCOI_pos2 = 545-1202\3;

GeneCOI_pos3 = 546-1202\3;

GeneCYTB_pos1 = 1203-2232\3;

GeneCYTB_pos2 = 1204-2232\3;

GeneCYTB_pos3 = 1205-2232\3;

GeneRag2_pos1 = 2233-3202\3;

GeneRag2_pos2 = 2234-3202\3;

GeneRag2_pos3 = 2235-3202\3;

GeneRag1_pos1 = 3203-4539\3;

GeneRag1_pos2 = 3204-4539\3;

GeneRag1_pos3 = 3205-4539\3;

GeneZic1_pos1 = 4540-5277\3;

GeneZic1_pos2 = 4541-5277\3;

GeneZic1_pos3 = 4542-5277\3;

## SCHEMES, search: all | greedy | cluster | cluster | user ##

[schemes]

search = greedy;

#user schemes go here if search=user. See manual for how to define#

################################################

##NEXUS FILE – GARLI (ML) – GYMNOTIFORMES##

################################################

#NEXUS

BEGIN TAXA;

TITLE Gymn_TAXA_Block_1;

DIMENSIONS NTAX=166;

TAXLABELS

Electrophorus_electricus_39371

Gymnotus_pantherinus_11144

.

.

.

Porotergus_gimbeli_178277

;

END;

BEGIN CHARACTERS;

TITLE Molecular_DATA_Block_1;

LINK TAXA = Gymn_TAXA_Block_1;

dimensions ntax=166 nchar=5277;

format data type=dna missing=? gap=-;

MATRIX

Electrophorus_electricus_39371 TTTAACGGCCGCGGT...

Gymnotus_pantherinus_11144 TTTAACGGCCGCGGT...

.

.

.

Porotergus_gimbeli_178277 TTTAACGGCCGCGGT...

;

END;

BEGIN SETS;

charset p1 = 1-543;

charset p2 = 544-1202\3;

charset p3 = 545-1202\3;

charset p4 = 546-1202\3;

charset p5 = 1203-2232\3;

charset p6 = 1204-2232\3;

charset p7 = 1205-2232\3;

charset p8 = 2233-3202\3;

charset p9 = 2234-3202\3;

charset p10 = 2235-3202\3;

charset p11 = 3203-4539\3;

charset p12 = 3204-4539\3;

charset p13 = 3205-4539\3;

charset p14 = 4540-5277\3;

charset p15 = 4541-5277\3;

charset p16 = 4542-5277\3;

charpartition byPos = part1:p1, part2:p2, part3:p3, part4:p4, part5:p5, part6:p6, part7:p7, part8:p8, part9:p9, part10:p10, part11:p11, part12:p12, part13:p13, part14:p14, part15:p15, part16:p16

;

END;

BEGIN CHARACTERS;

TITLE Morphology_DATA_Block_1;

LINK TAXA = Gymn_TAXA_Block_1;

DIMENSIONS NTAX=166 NCHAR=223;

FORMAT SYMBOLS= "0 1 2 3 4" MISSING=? GAP=- ;

CHARSTATELABELS

1 Body shape / Laterally compressed 'Sub-cylindrical',

2 Body_shape_2 / Laterally compressed Dorsovent_flattened,

.

.

.

223 EOD_monophasic_in_adults / absent present

;

MATRIX

Electrophorus_electricus_39371 101001000000...

Gymnotus_pantherinus_11144 101000110110...

.

.

.

Porotergus_gimbeli_178277 001001020000...

;

END;

#################################################

##CONFIG FILE – GARLI (ML) – GYMNOTIFORMES##

#################################################

[general]

datafname = GYMN_MolMol_GARLI.nex

constraint file = none

streefname = random

attachmentspertaxon = 100

of prefix = mixed_GYMN_MolMol_02

randseed = -1

availablememory = 512

logevery = 10

saveevery = 100

refinestart = 1/Users/... /Garli-2.01-IntelOSX_MorMol_166/bin/GYMNgarli.conf

outputeachbettertopology = 0

outputcurrentbesttopology = 0

enforcetermconditions = 1

genthreshfortopoterm = 10000

scorethreshforterm = 0.001

significanttopochange = 0.01

outputphyliptree = 0

outputmostlyuselessfiles = 0

writecheckpoints = 0

restart = 0

outgroup = Carassius_auratus_040213

resampleproportion = 1.0

inferinternalstateprobs = 0

outputsitelikelihoods = 0

optimizeinputonly = 0

collapsebranches = 1

searchreps = 2

bootstrapreps = 50

linkmodels = 0

subsetspecificrates = 1

[model1]

data type = nucleotide

ratematrix = 6rate

statefrequencies = estimate

ratehetmodel = gamma

numratecats = 4

invariantsites = none

[model2]

data type = nucleotide

ratematrix = 6rate

statefrequencies = estimate

ratehetmodel = gamma

numratecats = 4

invariantsites = none

[model3]

data type = nucleotide

ratematrix = (0 1 2 3 1 4)

statefrequencies = estimate

ratehetmodel = gamma

numratecats = 4

invariantsites = estimate

[model4]

data type = nucleotide

ratematrix = ( 0 1 2 2 3 0)

statefrequencies = estimate

ratehetmodel = gamma

numratecats = 4

invariantsites = estimate

[model5]

data type = nucleotide

ratematrix = 6rate

statefrequencies = estimate

ratehetmodel = gamma

numratecats = 4

invariantsites = estimate

[model6]

data type = nucleotide

ratematrix = 6rate

statefrequencies = estimate

ratehetmodel = gamma

numratecats = 4

invariantsites = estimate

[model7]

data type = nucleotide

ratematrix = 6rate

statefrequencies = equal

ratehetmodel = gamma

numratecats = 4

invariantsites = estimate

[model8]

data type = nucleotide

ratematrix = (0 1 2 3 1 4)

statefrequencies = equal

ratehetmodel = gamma

numratecats = 4

invariantsites = estimate

[model9]

data type = nucleotide

ratematrix = (0 1 2 3 1 4)

statefrequencies = estimate

ratehetmodel = gamma

numratecats = 4

invariantsites = estimate

[model10]

data type = nucleotide

ratematrix = 6rate

statefrequencies = equal

ratehetmodel = gamma

numratecats = 4

invariantsites = estimate

[model11]

data type = nucleotide

ratematrix = (0 1 2 3 1 4)

statefrequencies = estimate

ratehetmodel = gamma

numratecats = 4

invariantsites = none

[model12]

data type = nucleotide

ratematrix = (0 1 2 3 1 4)

statefrequencies = estimate

ratehetmodel = gamma

numratecats = 4

invariantsites = none

[model13]

data type = nucleotide

ratematrix = 6rate

statefrequencies = estimate

ratehetmodel = gamma

numratecats = 4

invariantsites = none

[model14]

data type = nucleotide

ratematrix = (0 1 2 3 1 4)

statefrequencies = estimate

ratehetmodel = gamma

numratecats = 4

invariantsites = none

[model15]

data type = nucleotide

ratematrix = 2rate

statefrequencies = estimate

ratehetmodel = gamma

numratecats = 4

invariantsites = none

[model16]

data type = nucleotide

ratematrix = (0 1 0 0 2 0)

statefrequencies = estimate

ratehetmodel = gamma

numratecats = 4

invariantsites = none

[model17]

data type = standardvariable

ratematrix = 1rate

statefrequencies = equal

ratehetmodel = none

numratecats = 1

invariantsites = none

[master]

nindivs = 4

holdover = 1

selectionintensity = 0.5

holdoverpenalty = 0

stopgen = 5000000

stoptime = 5000000

startoptprec = 0.5

minoptprec = 0.01

numberofprecreductions = 10

treerejectionthreshold = 50.0

topoweight = 0.01

modweight = 0.002

brlenweight = 0.002

randnniweight = 0.1

randsprweight = 0.3

limsprweight = 0.6

intervallength = 100

intervalstostore = 5

limsprrange = 6

meanbrlenmuts = 5

gammashapebrlen = 1000

gammashapemodel = 1000

uniqueswapbias = 0.1

distanceswapbias = 1.0

#############################################

##NEXUS FILE – MRBAYES (BI) – GYMNOTIFORMES##

#############################################

#NEXUS

BEGIN DATA;

DIMENSIONS NTAX=166 NCHAR=5500;

FORMAT DATATYPE = mixed(dna: 1-5277, standard: 5278-5500) gap = - missing =?;

MATRIX

Electrophorus_electricus_39371 TTTAACGGCCC... 101001000...

Gymnotus_pantherinus_11144 TTTAACGGCCG... 101000110...

.

.

.

Porotergus_gimbeli_178277 TTTAACGGCCG... 001001020...

;

END;

BEGIN SETS;

CHARPARTITION * matrices = Character_Matrix : 1-5277 , Character_Matrix2 : 5278-5500;

charset Character_Matrix = 1-5277;

charset Character_Matrix2 = 5278-5500;

END;

BEGIN MRBAYES;

charset Character_Matrix_p1 = 1-543;

charset Character_Matrix_p2 = 544-1202\3;

charset Character_Matrix_p3 = 545-1202\3;

charset Character_Matrix_p4 = 546-1202\3;

charset Character_Matrix_p5 = 1203-2232\3;

charset Character_Matrix_p6 = 1204-2232\3;

charset Character_Matrix_p7 = 1205-2232\3;

charset Character_Matrix_p8 = 2233-3202\3;

charset Character_Matrix_p9 = 2234-3202\3;

charset Character_Matrix_p10 = 2235-3202\3;

charset Character_Matrix_p11 = 3203-4539\3;

charset Character_Matrix_p12 = 3204-4539\3;

charset Character_Matrix_p13 = 3205-4539\3;

charset Character_Matrix_p14 = 4540-5277\3;

charset Character_Matrix_p15 = 4541-5277\3;

charset Character_Matrix_p16 = 4542-5277\3;

charset Character_Matrix2 = 5278-5500;

partition matrices = 17: Character_Matrix_p1, Character_Matrix_p2, Character_Matrix_p3, Character_Matrix_p4, Character_Matrix_p5, Character_Matrix_p6, Character_Matrix_p7, Character_Matrix_p8, Character_Matrix_p9, Character_Matrix_p10, Character_Matrix_p11, Character_Matrix_p12, Character_Matrix_p13, Character_Matrix_p14, Character_Matrix_p15, Character_Matrix_p16, Character_Matrix2;

set partition = matrices;

unlink statefreq=(all) revmat=(all) shape=(all) pinvar=(all);

prset applyto=(all) ratepr=variable;

lset applyto=(1) nst=6 rates=gamma;

lset applyto=(2) nst=6 rates=gamma;

lset applyto=(3) nst=6 rates=gamma;

lset applyto=(4) nst=6 rates=gamma;

lset applyto=(5) nst=6 rates=gamma;

lset applyto=(6) nst=2 rates=gamma;

lset applyto=(7) nst=6 rates=gamma; prset applyto=(7) statefreqpr=fixed(equal);

lset applyto=(8) nst=6 rates=gamma; prset applyto=(8) statefreqpr=fixed(equal);

lset applyto=(9) nst=6 rates=gamma;

lset applyto=(10) nst=6 rates=gamma; prset applyto=(10) statefreqpr=fixed(equal);

lset applyto=(11) nst=6 rates=gamma; prset applyto=(11) statefreqpr=fixed(equal);

lset applyto=(12) nst=2 rates=gamma;

lset applyto=(13) nst=6 rates=gamma;

lset applyto=(14) nst=6 rates=gamma;

lset applyto=(15) nst=2 rates=gamma;

lset applyto=(16) nst=2 rates=gamma;

lset applyto=(17) nst=1 rates=gamma coding=variable;

outgroup Carassius_auratus_040213;

mcmcp

ngen= 50000000

relburnin=yes

burninfrac=0.25

printfreq=5000

samplefreq=5000

nchains=4

nruns=2

savebrlens=yes;

mcmc;

sump;

sumt;
