# Supplementary material for: Data supporting phylogenetic reconstructions of the Neotropical clade Gymnotiformes
Source: Data Brief. 2016 Feb 6;7:23–59. doi: 10.1016/j.dib.2016.01.069 (PMC4761620; doi:10.1016/j.dib.2016.01.069)
Supplement: Supplementary file 4 — Supplementary material [file mmc4.doc]

'Conflicts of interest: none'
